# Shape and amplitude decoupling in pulsatile physiological signal synthesis and its evaluation

**DOI:** 10.1038/s41467-026-72299-7

**Published:** 2026-04-29

**Authors:** Junetae Kim, Kyoungsuk Park, Lei Chen, Kyunglim Kim

**Affiliations:** 1 Graduate School of Cancer Science and Policy, National Cancer Center, 323 Ilsan-ro, Ilsandong-gu, Goyang-si, Gyeonggi-do, Republic of Korea; 2 Healthcare AI Team, National Cancer Center, 323 Ilsan-ro, Ilsandong-gu, Goyang-si, Gyeonggi-do, Republic of Korea; 3 Samsung Research, 56 Seongchon-gil, Seocho-gu, Seoul, Republic of Korea

**Keywords:** Computational models, Computer science, Biomedical engineering, Statistics, Machine learning

## Abstract

Pulsatile physiological signals, such as arterial blood pressure and electrocardiograms, encode cardiovascular dynamics through rhythmic variations in waveform shape and amplitude. Controlled synthesis of such signals is critical for advancing physiological understanding and clinical applications. However, most existing generative methods represent waveform shape and amplitude in a single, mixed form. This coupling constrains the ability to adjust one without affecting the other, thereby limiting controllability and interpretability in signal generation. We present VABAM, a generative framework that operates on a single physiological signal to decouple waveform shape and amplitude through cascaded filtering. This decoupling enables targeted amplitude modulation while preserving waveform shape. To assess the synthesis quality, we introduce four metrics that quantify waveform shape factorization, shape preservation, amplitude modulation controllability, and spectral similarity, alongside conventional reconstruction accuracy. Across multiple benchmark datasets, VABAM outperforms existing methods, demonstrating the significance of waveform shape-amplitude decoupling for controlled physiological signal generation. This may enable amplitude-targeted augmentation, uncertainty-quantified prediction, and enhanced real-time anomaly monitoring, thereby advancing clinical decision-making in physiological signal analysis.

## Abstract (web summary)

VABAM decouples waveform shape and amplitude in physiological signals via cascaded filtering, enabling controlled generation. It improves synthesis quality and interpretability, supporting targeted augmentation and more reliable clinical analysis.

## Introduction

Pulsatile physiological signals are time-varying waveforms that originate from periodic cardiovascular and respiratory activity^1,2^. These include arterial blood pressure (ABP) waveforms and photoplethysmogram (PPG), which convey rich information about underlying hemodynamic states, and electrocardiogram (ECG), which reflects the electrical activity of the cardiac effector^3,4^. Such signals form the basis of dynamic cardiovascular assessment and are widely used in clinical monitoring, wearable health technologies, and intensive care systems^5–8^.

Unlike images or natural language, which exhibit flexible spatial or semantic structures, physiological signals encode intrinsic rhythmic and temporal patterns that manifest as waveform shape and amplitude dynamics^1,2^. Waveform shape typically refers to the temporal arrangement of onset and offset points, peaks, and valleys within a cycle, while amplitude variation denotes changes in signal magnitude independent of shape^3,9^. Physiologically, waveform shape and amplitude represent distinct information sources: shape reflects the dynamic processes and system properties governing signal generation and propagation, while amplitude encodes the quantitative state of the underlying variable^3,9^. This distinction calls for generative frameworks specifically designed to capture domain-specific signal properties. Recent advances in deep generative modeling have enabled a range of applications, including waveform synthesis, modality translation, and signal restoration^4^. Among these, diffusion-based models have demonstrated strong capabilities in synthesizing physiologically realistic signals by learning to reverse stochastic forward processes^10–12^.

However, diffusion-based models synthesize physiological signals through denoising processes that couple waveform shape and amplitude without distinguishing their different origins. This prevents independent control over shape and amplitude variations during synthesis, thereby limiting the ability to simulate physiological diversity^13^, model sensor variability^4^, and mitigate signal artifacts^14^. For example, generating patient-specific ECG signals requires preserving diagnostic waveform shapes while varying amplitude across individuals or devices, which coupled representations may not achieve.

Alternatively, latent-variable models that promote disentangled representations may offer more precise control over waveform shape and amplitude variations. Extensions of variational autoencoders (VAEs) achieve this through conditional inputs^15^, statistical independence enforced through regularization^16,17^, and the adoption of deep hierarchical structures^18^. When applied to physiological signals, these models have been used for ECG beat embedding^19^, ventricular tachycardia localization^20^, and heart rate anomaly detection^21^. However, while these approaches promote disentangled latent spaces, they operate within a shared representation and lack explicit mechanisms to decouple waveform shape from amplitude variation.

**Fig. 1:**
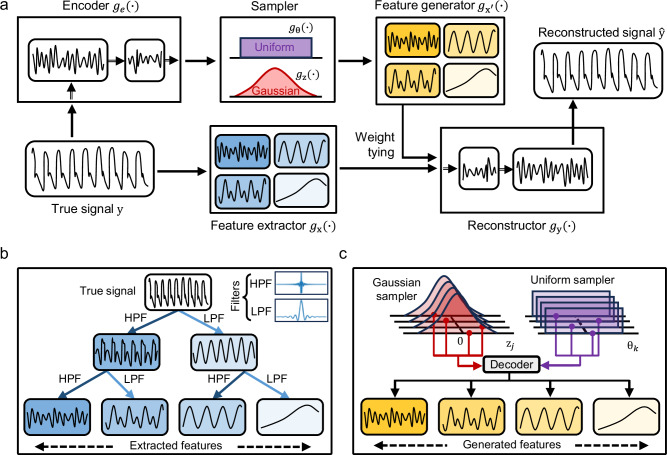
Schematic overview of the proposed VABAM framework for shape-amplitude decoupling. **a** Overall architecture of the framework illustrating the encoder-reconstructor pipeline, in which raw signals are decomposed into latent representations, sampled using uniform and Gaussian distributions, and reconstructed through amplitude-wise synthesis with shared reconstruction weights between the feature extractor (training) and feature generator (generation) modules. **b** Detailed structure of the feature extractor employing a cascaded filtering mechanism with high-pass filters (HPF) and low-pass filters (LPF) to hierarchically decompose raw signals into frequency-specific feature representations across multiple scales (illustrated for depth *ζ* = 2, generalizable to arbitrary *ζ* = *λ*). **c** Architecture of the feature generator with samplers in which latent codes **z** (waveform shape) and **θ** (amplitude) are drawn from Gaussian and uniform distributions, respectively, and then passed through decoder layers to synthesize frequency-decomposed features.

To address these limitations, we decouple amplitude from waveform shape in pulsatile physiological signals (Fig. 1). Unlike disentanglement, which promotes statistical independence among latent factors within a shared latent space, we structurally partition the generative process to establish dedicated pathways for each component. Drawing inspiration from cascaded filtering–a hierarchical signal processing technique that applies frequency-selective operations to progressively isolate distinct spectral subcomponents of temporal data^22^–we separate amplitude-sensitive components from shape patterns, enabling controllable and physiologically grounded synthesis.

To implement this structural partitioning, we propose VABAM (Variational Autoencoder for Amplitude-based Biosignal Augmentation within Morphological Shape), which extends variational inference through a cascaded filtering architecture. In this design, learnable cutoff frequencies (i.e., cascaded filter coefficients) define spectral retention at each hierarchical filtering stage (Fig. 1b and Equation (1)), optimized via backpropagation under a uniform prior constraint. This probabilistic constraint encourages diverse frequency filtering across stages, promoting broad spectral coverage and stable morphological representations. The resulting filtered subsets are encoded as latent representations that provide waveform shape grounding for signal synthesis, while the cascaded filter coefficients fuse with these representations to enable amplitude modulation.

**Fig. 2:**
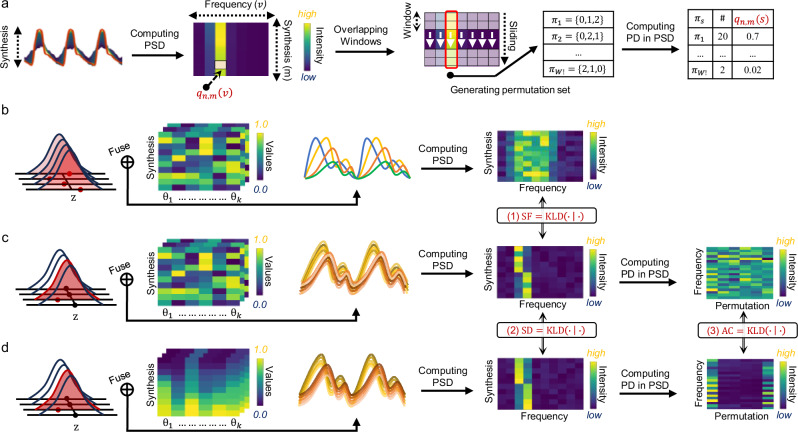
Schematic overview for assessing waveform shape-amplitude decoupling quality. **a** Procedure for computing power spectral density (PSD) representations, *q*(*v*), followed by construction of permutation distributions (PD) in the PSD domain, *q*(*s*). Here, *π* and *#* denote permutation and its number of observations, respectively. **b** Signal synthesis using all activated shape codes **z**, combined with multiple amplitude codes **θ**, to generate waveform sets, followed by PSD computation. **c** Signal synthesis using a single activated (nonzero) shape dimension with varying amplitude codes, followed by PSD computation and PD analysis across frequency space. **d** Signal synthesis using a single activated shape dimension while systematically varying amplitude codes, followed by PSD and PD computation across frequency-permutation space. Three metrics are derived through Kullback-Leibler divergence (KLD) analysis: (1) waveform shape factorization (SF), (2) shape distortion (SD), and (3) amplitude controllability (AC). The SD metric is subsequently inverse-transformed to yield a waveform shape preservation (SP)-based measure. Color maps indicate **θ** values or intensity levels, respectively. This schematic illustrates the conceptual workflow; detailed methodology is provided in the supplementary information.

To evaluate the decoupling, we introduce five metrics. (i) shape factorization: evaluates whether the latent space disentangles waveform shapes along specific dimensions, which is essential for representing distinct shape features while allowing independent amplitude control (Fig. 2b and c)^23^. (ii) shape preservation: evaluates the model’s ability under conditional inputs to maintain waveform shape while adjusting amplitude, which is critical for consistent synthesis (Fig. 2c and d). (iii) amplitude modulation controllability: assesses whether amplitude can be modulated accurately within a fixed waveform shape based on the intended input, which is crucial for precise and reliable control (Fig. 2c and d). (iv) spectral similarity: measures the agreement between the frequency characteristics of real and generated signals, which is particularly relevant in physiological contexts given their clinical significance^24^. (v) reconstruction accuracy: quantifies generative precision using the coefficient of determination (*R*^2^), a standard measure of reconstruction fidelity. The first four metrics are derived from conditional mutual information (CMI)^25^, reflecting the probabilistic structure of our model and other variational approaches. All the five metrics are integrated into a single composite score (ISCORE), which provides a unified evaluation of generative performance. ISCORE captures balanced performance across trade-offs that often arise among individual metrics, enabling consistent model comparisons and offering a holistic reflection of both structural quality and generative fidelity^26,27^.

Empirical results demonstrate that VABAM enables decoupled synthesis of pulsatile physiological signals, allowing controllable amplitude modulation while maintaining waveform shape consistency across both ABP and ECG signals from multisource datasets. Furthermore, the proposed CMI-based evaluation effectively captures structural synthesis quality and aligns closely with qualitative observations, providing a principled basis for model selection and hyperparameter tuning.

## Results

### Model architectures and training strategies

VABAM, its ablation variants, and benchmark baselines were trained on 10 s single-channel waveforms of ABP and ECG signals obtained from the MIMIC-III^28^ and VitalDB^29^ databases. These waveforms underwent multi-stage preprocessing–including missing value removal, physiological outlier filtering, peak-based anomaly detection^30^, and temporal resampling from 125 Hz to 100 Hz^31^, to ensure high-quality inputs for training. After preprocessing, each signal type and database yielded 400,000 training samples, 50,000 validation samples, and 20,000 test samples.

**Fig. 3:**
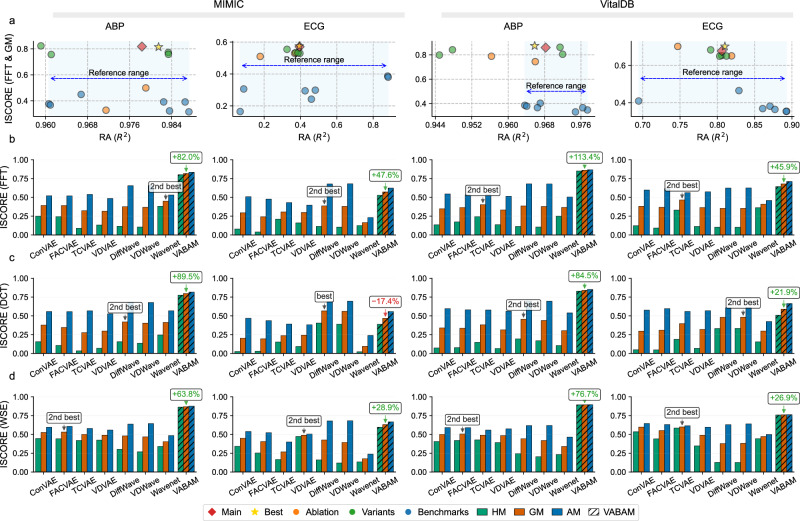
Integration-level evaluation of generative models for physiological signals. **a** Analysis of generative quality relative to reconstruction accuracy (RA). Scatter plots show ISCORE, aggregated using the geometric mean with the Fast Fourier Transform method, plotted against RA (*R*^2^) for arterial blood pressure (ABP) and electrocardiogram (ECG) signals across the MIMIC and VitalDB datasets. Shaded regions indicate the reference RA ranges within which our main model was optimized. **b** ISCORE-based evaluation using Fast Fourier Transform (FFT). **c** ISCORE-based evaluation using Discrete Cosine Transform (DCT). **d** ISCORE-based evaluation using Welch-based spectral evolution (WSE). In **b**–**d**, bars represent the harmonic mean (HM), geometric mean (GM), and arithmetic mean (AM) of the integrated score (ISCORE). Percentage annotations indicate geometric mean improvements of ISCORE relative to the second-best model when VABAM ranked first, and relative to the best model otherwise, across datasets, signal types, and method domains. Benchmark models are as follows: ConVAE (Conditional VAE), FACVAE (Factor VAE), TCVAE (Total Correlation VAE), VDVAE (Very Deep VAE), DiffWave (Diffusion WaveNet), VDWave (Variational Diffusion WaveNet), and WaveNet.

VABAM and its variants employ stacked bidirectional gated recurrent unit (GRU) layers as their core architecture (Supplementary Figs. S1 and S2). As a training constraint, we first established a reference *R*^2^ range by training benchmark models. The main model was then optimized to maintain its *R*^2^ within this range (Fig. 3a). This constraint prevents overfitting to reconstruction accuracy, which can obscure decoupling properties such as shape factorization and amplitude modulation controllability owing to inherent trade-offs between reconstructive accuracy and generative decoupling^17^.

For the main model, we defined three architectural hyperparameters. The cascaded filter depth (*ζ* = 1) controls the number of filtering stages used to extract frequency-specific features from the input waveform. The latent dimension size (*J* = 30) determines the capacity of the latent space used to encode waveform shape. The compressed feature length (*C* = 800, 8 s at 100 Hz) specifies the length of the compressed temporal representation obtained after the filtering module. These three parameters were varied to assess the sensitivity of model performance to changes in each configuration. Based on the best-performing setup, we conducted ablation studies by removing prior constraints, specifically the normal prior on the latent shape space **z** and the uniform prior on the amplitude modulation parameter **θ**, to evaluate their individual contributions to the model’s synthesis behavior.

### Strategies for quantitative evaluation

We quantitatively evaluated the performance of VABAM against seven benchmark models using a shared set of evaluation metrics. Each model was trained on one dataset (VitalDB or MIMIC) and evaluated on the other to assess generalization under domain shift. All approaches incorporate conditional inputs, **θ** during signal synthesis but differ in how these inputs are constructed. In our model, the conditional input is defined as a frequency cutoff value used to configure a series of cascaded filters. These values are uniformly sampled and sorted by synthesis index to form structured inputs, $\widehat{{{{\mathbf{\uptheta }}}}}$, for generation. Benchmark models, by contrast, compute the normalized power spectral density (PSD) of the true signal and sort the resulting values by synthesis index. This distinction reflects a fundamental design difference: our model uses externally defined control parameters, while the baselines derive conditioning information directly from the true data.

The benchmark models fall into three primary architectural families. VAE-based models, including Conditional VAE (ConVAE)^15^, Factor VAE (FACVAE)^17^, Total Correlation VAE (TCVAE)^16^, and Very Deep VAE (VDVAE)^18^, employ latent-variable frameworks that promote disentangled representations. Autoregressive models are represented by Conditional WaveNet^32^, which uses causal convolutions to enforce local continuity during generation. Diffusion-based models include Diffusion WaveNet (DiffWave)^10^, which generates signals through iterative denoising conditioned on spectral information^33^, and Variational Diffusion WaveNet (VDWave), which extends this approach by incorporating a variational diffusion process for enhanced latent expressivity and regularization^34^. These models were adapted for the waveform shape-amplitude decoupling task while preserving their core mechanisms.

CMI-based metrics were computed using three PSD estimation methods: Fast Fourier Transform (FFT)^24^, Welch-based spectral evolution (WSE)^35^, and Discrete Cosine Transform type-II (DCT)^36^ to capture complementary aspects of spectral characteristics. All five metrics were normalized to the range 0-1, with higher values indicating superior generative quality. ISCORE was computed by combining the normalized metrics using arithmetic mean (AM), geometric mean (GM), and harmonic mean (HM), enabling assessment of performance balance across metrics and robustness to uneven metric behavior^26,27^.

Unlike VABAM and VAE-based architectures, diffusion-based and autoregressive models do not employ explicitly disentangled representations^10,32,34^. Their outputs are instead governed by holistic latent trajectories or global conditioning mechanisms. Because the waveform shape factorization metric depends on factorized latent manipulations, it was not directly applicable to these models and was therefore excluded from ISCORE computation. Accordingly, these models were evaluated using four metrics. To ensure fair comparison, our model—although primarily assessed using all five metrics—was additionally evaluated with the same four metrics as the diffusion-based and autoregressive models.

### Strategies for qualitative evaluation

We conducted a qualitative evaluation using two strategies applied to VABAM, as well as to VDVAE and VDWave, which represent recent high-performing VAE- and diffusion-based models. The first strategy evaluated each model’s ability to modulate amplitude in response to structured conditional inputs while preserving the waveform shape of a given ABP or ECG signal across 600 synthesized samples. In VABAM, **θ** was uniformly sampled from [0, 1] and sorted to form a monotonically ordered conditioning sequence $\widehat{{{{\mathbf{\uptheta }}}}}$. For the benchmark models, normalized PSDs obtained using the FFT method were randomly sampled from ground-truth signals, and **θ** was defined as the index of the frequency band with maximum power in each case. These indices were then sorted to construct a comparable conditioning sequence $\widehat{{{{\mathbf{\uptheta }}}}}$.

The second strategy evaluated spectral responsiveness across heterogeneous data sources. For each model, **θ** was discretized into five bins, and conditional synthesis was performed for each bin using a set of 10,000 signals. The resulting PSDs were compared with those of the original signals to assess responsiveness.

The definition of **θ** varied across models. In VABAM, **θ** ∈ [0, 1] represents the cutoff frequency in the cascaded filters and was uniformly partitioned into five bins. In the benchmark models, **θ** ∈ {1, …, 50} denotes the frequency index with maximal spectral power, reflecting dominant spectral dynamics and amplitude features^24^. These indices were subsequently grouped into the same five-bin structure to enable direct comparison across models.

### Quantitative results on benchmark tasks

Benchmark comparisons across FFT, DCT, and WSE methods demonstrated consistent advantages for the proposed model (Fig. 3; Supplementary Tables S1–S3). VABAM achieved the highest ISCORE across datasets and signal types, substantially outperforming benchmark models under all averaging schemes, except for ECG under DCT, where diffusion-based models performed better. When all five metrics were included, VABAM ranked first in 11 of the 12 configurations based on the GM, with relative differences from the best-performing alternative ranging from −17.4% to +113.4% (Fig. 3). When four metrics were considered—excluding waveform shape factorization—performance remained competitive, again ranking first in 11 of the 12 configurations (Supplementary Tables S1–S3).

Averaging schemes revealed clear differences in model balance. HM and GM values were consistently lower than AM across all models, with diffusion-based models exhibiting the largest HM-AM gaps, ranging from 0.276 to 0.575 (Fig. 3; Supplementary Tables S1–S3). VAE-based models exhibited moderate gaps of 0.03 to 0.549, whereas VABAM maintained minimal dispersion of 0.002 to 0.169, indicating balanced performance.

PSD methods influenced the magnitude of performance differences. The WSE method yielded the highest overall ISCORE values and revealed larger contrasts between models (GM: 0.175 to 0.599 for benchmark models vs. 0.631 to 0.894 for VABAM), particularly for shape preservation (0.077 to 0.954 for benchmark models vs. 0.599 to 0.887 for VABAM) (Fig. 3; Supplementary Table S3). Diffusion-based models benefited from the DCT method on MIMIC-ECG (GM: 0.561 to 0.566 for diffusion models vs. 0.387 to 0.468 for VABAM) (Fig. 3; Supplementary Table S2), whereas VABAM remained stable across all PSD estimation methods, showing no dependence on a single spectral representation.

**Fig. 4:**
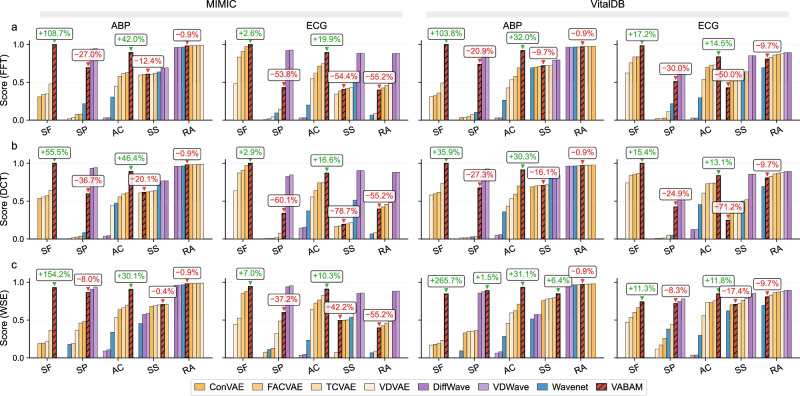
Metric-level evaluation of generative models for physiological signals. **a** Metric-based evaluation using Fast Fourier Transform (FFT). **b** Metric-based evaluation using Discrete Cosine Transform (DCT). **c** Metric-based evaluation using Welch-based spectral evolution (WSE). Performance was evaluated across five metrics: waveform shape factorization (SF), waveform shape preservation (SP), amplitude controllability (AC), spectral similarity (SS), and reconstruction accuracy (RA). Results are reported for arterial blood pressure (ABP) and electrocardiogram (ECG) signals from the MIMIC and VitalDB datasets. Percentage annotations indicate relative improvements within each metric, calculated against the second-best model when VABAM performed best and against the best model otherwise. Benchmark models are as follows: ConVAE (Conditional VAE), FACVAE (Factor VAE), TCVAE (Total Correlation VAE), VDVAE (Very Deep VAE), DiffWave (Diffusion WaveNet), VDWave (Variational Diffusion WaveNet), and WaveNet.

Individual metrics revealed distinct model specializations (Fig. 4; Supplementary Tables S1–S3). VABAM achieved near-perfect waveform shape factorization (0.740 to 1.0) and demonstrated the strongest amplitude controllability (0.836 to 0.931). Diffusion-based models excelled in shape preservation (0.557 to 0.954) and performed well in spectral similarity (0.572 to 0.906) but exhibited markedly limited amplitude controllability (0.028 to 0.156) and lacked an explicit mechanism for shape factorization. VAE-based models achieved moderate reconstruction accuracy (0.425 to 0.987, with one outlier at 0.088) but consistently poor shape preservation (0.006 to 0.486). WaveNet showed balanced yet suboptimal performance across all metrics, without excelling in any specific dimension. No benchmark model demonstrated competitive performance across all criteria, whereas VABAM maintained consistently robust results, exhibiting no major deficiencies and a stable performance across the complete metric set.

### Quantitative results of ablation studies

**Fig. 5:**
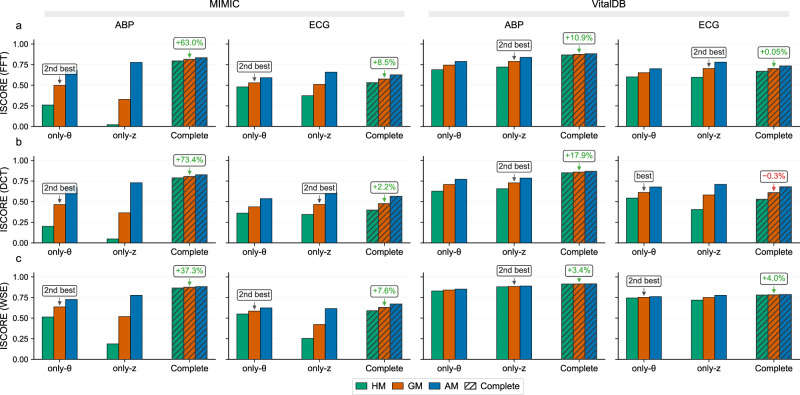
Ablation studies of generative models for physiological signals. **a** ISCORE-based evaluation using Fast Fourier Transform (FFT). **b** ISCORE-based evaluation using Discrete Cosine Transform (DCT). **c** ISCORE-based evaluation using Welch-based spectral evolution (WSE). Results are shown for ablation variants (only-**θ**, only-**z**) and the complete model across arterial blood pressure (ABP) and electrocardiogram (ECG) signals from the MIMIC and VitalDB datasets. Bars represent the harmonic mean (HM), geometric mean (GM), and arithmetic mean (AM) of the integrated score (ISCORE). Percentage annotations indicate geometric mean improvements relative to the second-best model when VABAM performed best, and relative to the best model otherwise, across datasets and power spectral density estimation methods.

Ablation studies on the role of prior constraints were performed under optimal hyperparameter settings (*C* = 800, *J* = 50, *ζ* = 1). These settings were determined by averaging ISCORE values across the three PSD estimation methods (FFT, DCT, and WSE) and selecting the configuration that maximized the GM across all dataset-signal combinations (Fig. 5; Supplementary Tables S4–S6).

Across all PSD methods and datasets, the complete model consistently achieved the highest ISCORE values. Under FFT and WSE, it outperformed both ablation variants in every configuration (Fig. 5a, c; Supplementary Tables S4 and S6). Comparable results were obtained with DCT (Fig. 5b; Supplementary Table S5), relative differences from the best-performing alternative ranging from −0.3% to +73.4%, depending on the dataset. WSE yielded the highest ISCORE values for the complete model, with GM ranging from 0.630 to 0.915 (Supplementary Table S6).

**Fig. 6:**
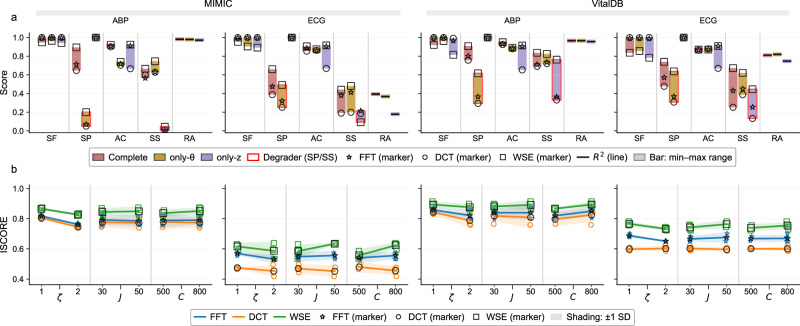
Performance range analysis of ablation models and sensitivity analysis of variant models. **a** Metric-level performance ranges for arterial blood pressure (ABP) and electrocardiogram (ECG) signals from MIMIC and VitalDB. Bars span the min–max range across power spectral density (PSD) estimation methods for five metrics: waveform shape factorization (SF), waveform shape preservation (SP), amplitude controllability (AC), spectral similarity (SS), and reconstruction accuracy (RA). Results are shown for the complete model, ablation variants (only-**θ**, only-**z**), and degraders exhibiting pronounced deficiencies in SP and SS, thereby contributing to overall ISCORE reduction. **b** Sensitivity analysis with respect to cascaded filter depth (*ζ*), latent dimension size (*J*), and compressed feature length (*C*). Lines indicate mean integrated score (ISCORE) across experimental runs; shading represents  ± 1 standard deviation (SD). Individual scores are overlaid as markers for each PSD estimation method.

Analysis of individual metrics revealed distinct trade-offs among the ablation variants (Fig. 6a; Supplementary Tables S4–S6). Shape preservation was near-perfect for the only-**z**-prior model (0.999 to 1.0) but substantially lower for the only-**θ**-prior model (0.051 to 0.638), while the complete model achieved intermediate values (0.388 to 0.908). On the other hand, spectral similarity was strong for the only-**θ**-prior (0.199 to 0.825) with the complete model exhibiting comparable performance (0.19 to 0.836), but markedly reduced for the only-**z**-prior (0.004 to 0.762). Shape factorization (0.783 to 1.0) and amplitude controllability (0.653 to 0.95) remained consistently high across all models. Reconstruction accuracy was lower for the only-**z**-prior (0.179 to 0.971) but remained high and stable for both the complete model (0.393 to 0.981) and the only-**θ**-prior (0.368 to 0.979) across datasets.

### Quantitative results of hyperparameter sensitivity

Sensitivity analysis, evaluated by averaging the three mean schemes across all experimental runs and hyperparameter settings, revealed overall consistency across PSD estimation methods, with only minor deviations, indicating the general robustness of these approaches (Fig. 6b; Supplementary Tables S7–S9). For both ABP and ECG signals, increasing the cascaded depth *ζ* from 1 to 2 consistently reduced ISCORE, driven primarily by declines in amplitude controllability (−0.033 to −0.16), with shape preservation showing mixed contributions (−0.173 to +0.12), while reconstruction-related metrics such as spectral similarity and accuracy remained high. Increasing the latent dimension *J* from 30 to 50 produced mixed results, with ISCORE improving in some configurations but declining in others. Extending the compressed feature length *C* from 500 to 800 generally improved ISCORE, primarily through gains in reconstruction accuracy (+0.009 to +0.026), though spectral similarity (−0.047 to +0.224) and amplitude controllability (−0.036 to  +0.034) showed mixed effects.

Overall patterns for FFT and WSE remained consistent across parameter changes, whereas DCT exhibited minor deviations in a few cases (Fig. 6b). In MIMIC-ECG, gains from increasing *J* or *C* were consistently reflected in FFT and WSE results, while DCT showed little change or slight declines. However, these exceptions did not affect the overall conclusions. Per-metric results and ISCORE under three averaging schemes are reported in Supplementary Tables S7–S9.

### Qualitative assessment of amplitude controllability under fixed waveform shape

**Fig. 7:**
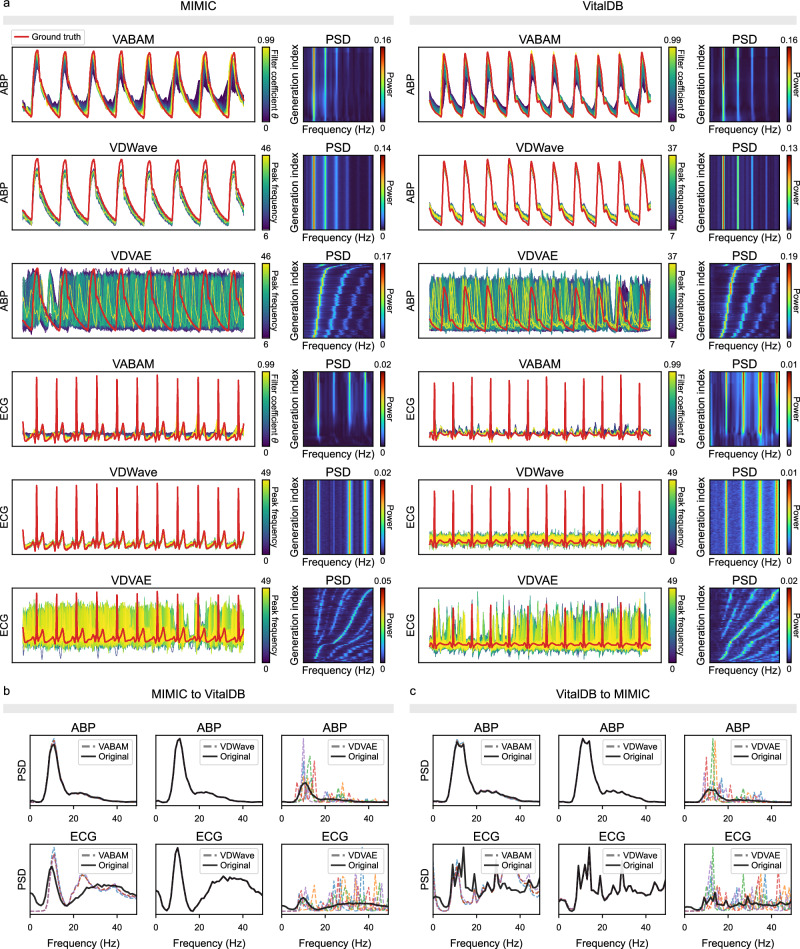
Qualitative analysis of amplitude controllability under preserved waveform shape and spectral responsiveness across three models: VABAM, VDWave (Variational Diffusion WaveNet), and VDVAE (Very Deep VAE). **a** Waveforms and power spectral density (PSD) heatmaps generated from structurally sorted input values were used to assess amplitude controllability. For VABAM, generation is conditioned on the cascaded filter coefficients **θ**, which directly modulate the signal synthesis process, whereas VDWave and VDVAE are conditioned on the input signal’s power spectrum summarized by its peak frequency bin. The conditioning variables are represented by their magnitudes in the colorbars shown within the waveform panels. **b** PSD distributions estimated using the Fast Fourier Transform (FFT) from MIMIC-trained models evaluated on VitalDB, illustrating spectral responsiveness to amplitude conditioning. **c** PSD distributions (FFT) from VitalDB-trained models evaluated on MIMIC, illustrating spectral responsiveness to amplitude conditioning.

Visual analysis highlights differences in the ability of models to modulate amplitude while preserving waveform shape, revealing varying levels of controllability across architectures (Fig. 7a). VDVAE demonstrates limited controllability, as the conditional input alters the overall waveform shape rather than modulating amplitude under a fixed shape, indicating insufficient decoupling. VDWave achieves high reconstruction fidelity but exhibits minimal responsiveness to conditioning: its PSD matrices display static vertical bands without progressive spectral changes, reflecting limited waveform variation and weak amplitude modulation with respect to **θ**.

VABAM consistently preserves waveform shape across the full range of **θ**. The synthesized signals exhibit smooth, monotonic amplitude modulation while maintaining waveform shape. The corresponding PSD matrices display clearly separated, vertically extended spectral bands that persist across synthesis conditions, indicating effective and stable integration of waveform shape and amplitude. Consequently, the model enables controllable and coherent signal synthesis across both the shape and amplitude domains.

### Qualitative assessment of spectral responsiveness

Spectral responsiveness analysis highlights clear differences in responsiveness to amplitude conditioning, ranging from smooth modulation to inconsistent or unresponsive patterns, as observed across datasets from VitalDB to MIMIC (Fig. 7b) and vice versa (Fig. 7c). VDWave produced PSDs that closely resembled those of the original signals but exhibited minimal variation across bins, indicating limited sensitivity to amplitude conditioning. In contrast, VDVAE exhibited significant spectral variability with unstable and spiky PSDs, failing to capture consistent global patterns or controlled modulation.

VABAM preserved the spectral pattern markedly better than VDVAE but slightly less well than VDWave, while enabling smooth, progressive changes in PSD intensity across conditioning bins. Spectral alignment with the original PSD was notably stronger for ABP signals, likely reflecting more consistent spectral distributions across domains, whereas alignment was weaker for ECG data, suggesting greater sensitivity to distributional shifts. These results confirm VABAM’s superior ability to achieve effective waveform shape-amplitude decoupling, while also indicating that generalization across heterogeneous data sources remains sensitive to spectral discrepancies.

## Discussion

The central contribution of this work is a principled framework for synthesizing pulsatile physiological signals through the explicit decoupling of waveform shape and amplitude dynamics. This separation, achieved via structured latent priors and a cascaded filtering mechanism, enables physiologically coherent and controllable waveform generation. Furthermore, this study advances the conceptual understanding of pulsatile signal synthesis by formalizing spectral characteristics as PSD-derived random variables within a probabilistic framework^24,37^, thereby facilitating principled evaluation based on conditional mutual information^25,38^. Collectively, this work establishes a unified and theoretically grounded framework encompassing both generative modeling and evaluation.

Empirical evaluations on ABP and ECG signals from MIMIC-III^28^ and VitalDB^29^ demonstrate that VABAM consistently outperformed competing approaches in waveform shape factorization and amplitude controllability, achieving higher ISCORE values and smaller discrepancies among the AM, GM, and HM than other models. This advantage arises from conditioning on the cascaded filtering mechanism, which explicitly partitions waveform shape and amplitude into separate generative pathways. This separation allows each component to be modeled independently, yielding clearer shape representations and finer amplitude control. In contrast, the VAE-, diffusion-, and autoregressive-based models in our experimental setting rely on PSD conditioning, which provides only statistical summaries of frequency-domain power and does not encode phase or time-localized shape information, thereby limiting effective shape-amplitude decoupling. Furthermore, the stochastic or autoregressive nature of the diffusion and WaveNet architectures appears to reduce the controllability by attenuating the conditioning effect.

VABAM maintained consistent rankings across PSD estimation methods, confirming its robustness to different spectral representations. Among these, WSE yielded the highest ISCORE values and sharper inter-model contrasts, likely due to its ability to capture non-stationary spectral dynamics^35^, whereas FFT and DCT produced slightly attenuated but comparable trends. Minor deviations observed in the diffusion models under DCT, which occasionally outperformed VABAM, did not alter the overall conclusions. These results provide consistent and reliable evidence that VABAM offers a robust foundation for controllable waveform synthesis.

This work also introduces a set of CMI-based metrics for the principled evaluation of synthesized signals. Unlike conventional measures focused solely on reconstruction fidelity or latent disentanglement^16,17^, the proposed information-theoretic metrics quantify independent control over waveform shape and amplitude, capturing shape factorization, shape preservation, and amplitude controllability. These metrics further support model selection and hyperparameter tuning by linking structural characteristics to generative performance, thereby advancing systematic assessment in physiological signal modeling.

**Fig. 8:**
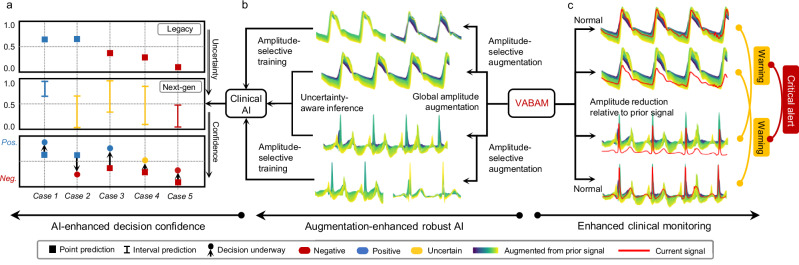
VABAM framework integrating uncertainty-quantified prediction, amplitude-targeted augmentation, and real-time clinical monitoring. **a** AI-enhanced decision confidence across five clinical cases. Legacy models generate single-point estimates, while next-generation models provide expanded uncertainty bounds. These uncertainty bounds enhance decision confidence and may influence clinical judgments that would otherwise rely solely on deterministic predictions. **b** Augmentation-enhanced robust AI. A bidirectional framework supports both amplitude-selective training and uncertainty-aware inference, each reinforced through amplitude augmentation. The system synthesizes physiologically plausible waveform variants to create scenario-specific ensembles, which can be leveraged by other clinical AI systems or applied directly in real-time physiological monitoring. **c** Enhanced clinical monitoring for real-time anomaly detection. Augmented reference bands derived from prior signals define expected variability ranges. Current waveforms are continuously evaluated against these probabilistic envelopes: conformity indicates normal status, a single-signal amplitude reduction suggests a mild warning, and sustained deviations across multiple signals indicate a critical physiological disturbance.

In practice, VABAM has the potential to unify predictive modeling, uncertainty quantification, and clinical interpretation. It generates physiologically plausible waveform variants tailored to specific clinical scenarios, such as lower amplitude-targeted augmentations to more sensitively assess hypotensive risk during surgery (Fig. 8b)^8^. These augmented ensembles capture both physiological variability and predictive uncertainty, providing a richer and more informative basis for precise and reliable clinical decision-making (Fig. 8a)^39^. Within this uncertainty-aware framework, VABAM supports real-time monitoring by generating reference bands (Fig. 8c), against which incoming waveforms are continuously compared. Departures in a single signal often reflect transient artifacts, while consistent deviations across multiple consecutive signals, rare under stable physiology, suggest genuine physiological disturbances^40–42^. Unlike conventional methods that aggregate multiple waveform cycles to establish the reference bands^43^, VABAM constructs them from a single preceding observation by generating multiple amplitude-varied augmentations, thereby improving both computational efficiency and early-warning sensitivity.

Despite these strengths, several challenges remain. VABAM’s spectral responsiveness may degrade under distributional shifts, particularly in ECG signals, which exhibit high inter-subject variability in our experiments. Future work could address this limitation by incorporating domain-adaptive priors or spectrum-aware regularization to improve generalization^4^. Additionally, VABAM assumes that shape and amplitude variations are separable—a simplification that may not fully capture higher-order coupling effects, particularly physiological shape-amplitude coupling^44,45^. This assumption may limit physiological realism in modeling complex waveform dynamics, underscoring the need for future architectures that better represent their interdependence. Moreover, CMI estimation in low-sample or high-dimensional settings remains a challenge^46^, potentially affecting evaluation reliability. This motivates the development of more scalable and statistically unbiased estimators.

Beyond addressing these limitations, several additional directions could further extend VABAM’s capabilities. These include expanding the current amplitude-based modulation to incorporate phase-wise control, enabling finer temporal manipulation and improved modeling of physiological variability, along with the integration of phase-aware metrics into the assessment framework. Extending VABAM to additional signal modalities and domain-transfer settings may also enhance its clinical applicability. Furthermore, integration with diffusion-based^33^ or Transformer-based architectures^47^ could advance spectrotemporal modeling and improve synthesis quality under complex physiological conditions.

In conclusion, this framework advances biomedical signal modeling and evaluation by bridging methodological innovation with translational potential. It establishes a foundation for controllable waveform synthesis and extends generative modeling principles across diverse physiological and time-series domains.

## Methods

### Cascaded filtering architecture

The cascaded filtering mechanism in VABAM decomposes the input signal into amplitude-modulated components across progressively narrower frequency bands^22^. At each stage of the cascade, a pair of low-pass and high-pass filters is applied to separate the low- and high-frequency components. The low-pass filter (LPF) is defined as

1 $${{{\rm{LPF}}}}[t]=\frac{\xi [t]w[t]}{{\sum }_{i=0}^{T-1}\xi [i]w[i]}\,with\,\xi [t]=sinc\left(2\theta \left(t-\frac{T-1}{2}\right)\right),$$

and the corresponding high-pass filter (HPF) is computed as HPF[*t*] = *δ*[*t*] − LPF[*t*]. Here, *θ* ∈ [0, 1] denotes a learnable cutoff frequency, *w*[*t*] is the Hamming window, which aims to attenuate sidelobes in the frequency domain and yield a smoother signal spectrum^37^, and *δ*[*t*] is the discrete-time impulse function that equals 1 at *t* = 0 and 0 elsewhere.

This filtering process is applied recursively over *λ* stages to form a binary decomposition tree (Fig. 1b). The cascade depth *ζ* = *λ*, defined as a model hyperparameter, determines the number of leaf-level outputs, yielding 2^*λ*^ subsets at the terminal nodes. The output subsets, ${{{\bf{x}}}}\in \{{{{{\bf{x}}}}}_{{2}^{\lambda }-1},{{{{\bf{x}}}}}_{{2}^{\lambda }},\ldots,{{{{\bf{x}}}}}_{{2}^{\lambda+1}-2}\}$, are frequency-localized representations of the original signal and serve as both input and reconstruction target within the model. This filtering hierarchy introduces $K={\sum }_{i=1}^{\lambda }{2}^{i}$ filter coefficients **θ**_*k*_, each optimized via backpropagation to enable data-driven spectral decomposition.

### Model components

VABAM comprises five trainable modules (Fig. 1): (1) the feature extractor *g*_**x**_( ⋅ ) applies cascaded filters to the observed true signal **y**, producing a set of filtered feature subsets; (2) the encoder *g*_*e*_( ⋅ ) infers variational distributions over two types of latent variables: the waveform shape-related vector **z** and the cutoff parameters **θ**, both of which are constrained by distinct prior assumptions. Each component **z**_*j*_ is modeled as a Gaussian distribution, ${{{{\bf{z}}}}}_{j} \sim {{{\mathcal{N}}}}(\,{\mu }_{{{{{\bf{z}}}}}_{j}},{\sigma }_{{{{{\bf{z}}}}}_{j}}^{2})$, while each cutoff parameter **θ**_*k*_ follows a uniform distribution, ${{{{\mathbf{\uptheta }}}}}_{k} \sim {{{\mathcal{U}}}}(0,1)$; (3) the sampler modules *g*_**z**_( ⋅ ) and *g*_**θ**_( ⋅ ) employ the reparameterization trick to draw differentiable samples from the respective latent spaces; (4) the feature generator ${g}_{{{{{\bf{x}}}}}^{{\prime} }}(\cdot )$ maps the sampled codes (**z**, **θ**) to generate feature subsets; and (5) the signal reconstructor *g*_**y**_( ⋅ ) combines these subsets to produce the reconstructed waveform $\widehat{{{{\bf{y}}}}}$.

### Variational learning objective

VABAM is trained by maximizing a variational lower bound on the marginal log-likelihood of the observed signal^48^. Given the generative structure of VABAM, the joint distribution over all variables can be factorized as

2 $$p({{{\bf{y}}}},{{{\bf{x}}}},{{{\bf{z}}}},{{{\mathbf{\uptheta }}}})=p({{{\bf{y}}}}| {{{\bf{x}}}})\,p({{{\bf{x}}}}| {{{\bf{z}}}},{{{\mathbf{\uptheta }}}})\,p({{{\bf{z}}}})\,p({{{\mathbf{\uptheta }}}}),$$

where *p*(**y**∣**x**) is associated with the signal reconstructor, *p*(**x**∣**z**, **θ**) with the feature generator, *p*(**z**) with the waveform shape prior, and *p*(**θ**) with the prior over the cutoff parameters. The variational approximation is defined as

3 $$q({{{\bf{x}}}},{{{\bf{z}}}},{{{\mathbf{\uptheta }}}}| {{{\bf{y}}}})=q({{{\bf{x}}}}| {{{\bf{y}}}},{{{\mathbf{\uptheta }}}})\,q({{{\bf{z}}}},{{{\mathbf{\uptheta }}}}| {{{\bf{y}}}}),$$

where *q*(**x**∣**y**, **θ**) is linked to the feature extractor, and *q*(**z**, **θ**∣**y**) to the encoder and sampler modules. By approximating the intractable true posterior *p*(**x**, **z**, **θ**∣**y**) with the variational distribution *q*(**x**, **z**, **θ**∣**y**), which is parameterized by neural networks and differentiable, this formulation enables gradient-based optimization^48^.

The final training objective, derived from the evidence lower bound (ELBO), comprises four terms:

4 $$J(\Phi ;{{{\bf{y}}}},{{{\bf{x}}}},{{{\bf{z}}}},{{{\mathbf{\uptheta }}}})=	{{\mathbb{E}}}_{q({{{\bf{x}}}},{{{\bf{z}}}},{{{\mathbf{\uptheta }}}}| {{{\bf{y}}}})}\Big[-\log p({{{\bf{y}}}}| {{{\bf{x}}}})-\log \frac{p({{{\bf{x}}}}| {{{\bf{z}}}},{{{\mathbf{\uptheta }}}})}{q({{{\bf{x}}}}| {{{\bf{y}}}},{{{\mathbf{\uptheta }}}})}\\ 	+\log \frac{q({{{\bf{z}}}}| {{{\bf{y}}}})}{p({{{\bf{z}}}})}+\log \frac{q({{{\mathbf{\uptheta }}}}| {{{\bf{y}}}})}{p({{{\mathbf{\uptheta }}}})}\Big],$$

where Φ represents all trainable network weights to be optimized.

Each term in the ELBO corresponds to a distinct modeling objective. The first term, ${{\mathbb{E}}}_{q({{{\bf{x}}}}| {{{\bf{y}}}},{{{\mathbf{\uptheta }}}})}[\log p({{{\bf{y}}}}| {{{\bf{x}}}})]$, promotes accurate signal reconstruction by encouraging the generated waveform to match the observed signal.

The second term, ${{\mathbb{E}}}_{q({{{\bf{z}}}},{{{\mathbf{\uptheta }}}}| {{{\bf{y}}}})q({{{\bf{x}}}}| {{{\bf{y}}}},{{{\mathbf{\uptheta }}}})}[\log p({{{\bf{x}}}}| {{{\bf{z}}}},{{{\mathbf{\uptheta }}}})]$, enforces faithful reconstruction of the extracted feature subsets by minimizing the discrepancy between **x**, obtained through the feature extractor, and $\widehat{{{{\bf{x}}}}}$, generated by the feature generator.

The third term,  − KL(*q*(**z**∣**y**)∥*p*(**z**)), employs the KL divergence to regularize the waveform shape latent variable by penalizing deviations from the standard Gaussian prior^48^.

The fourth term,  − KL(*q*(**θ**∣**y**)∥*p*(**θ**)), constrains the cascaded filter coefficients under a uniform prior, relaxed using a reparameterized Bernoulli distribution to enable gradient-based optimization^49,50^.

### Evaluation metrics

#### Information-theoretic foundations for evaluation

In our setting, CMI^25^ is used to evaluate how latent representations of waveform shape and amplitude-conditioning inputs influence the structure of the synthesized signal. Conceptually, it quantifies the conditional dependence between two variables given a third and is formally defined as

5 $$I(A;B| C)={{\mathbb{E}}}_{p(c)}[KL(p(a,b| c)\,\parallel \,p(a| c)p(b| c))],$$

where the expectation is taken with respect to the marginal distribution *p*(*c*). By definition, CMI is non-negative and equals zero if and only if *A* and *B* are conditionally independent given *C*^25^, thereby serving as an indicator of conditional dependence strength in probabilistic frameworks such as VABAM and VAE-based models.

#### Foundation of waveform shape factorization

To assess whether individual latent dimensions encode distinct waveform shapes, we modify the latent vector by selectively activating one component while suppressing the rest. Given a latent vector ${{{\bf{z}}}}={\{{{{{\bf{z}}}}}_{j}\}}_{j=1}^{J}$, we define a new vector $\widehat{{{{\bf{z}}}}}=\{{\widehat{{{{\bf{z}}}}}}_{j}\}$, randomly selecting a single index j^*^ and retaining ${\widehat{{{{\bf{z}}}}}}_{{j}^{*}}={{{{\bf{z}}}}}_{{j}^{*}}$, while setting all other elements to their posterior expectations, ${{\mathbb{E}}}_{q}[{{{{\bf{z}}}}}_{j}| {{{\bf{y}}}}]$. These expectations typically remain near zero owing to KL divergence regularization, which aligns the posterior *q*(**z**_*j*_∣**y**) with the standard normal prior $p({{{{\bf{z}}}}}_{j})={{{\mathcal{N}}}}(0,I)$^48^. This construction enables post hoc interpretation of individual latent dimensions, as inactive components contribute minimally to the output unless the conditional input drives their posterior away from the prior mean.

#### Foundation of waveform characteristics

To quantify waveform characteristics, we analyze synthesized signals in the frequency domain via their PSD, which captures key structural properties of waveforms (Fig. 2a)^24^. The PSD at frequency *v* is normalized to form a probability distribution,

6 $${q}_{n,m}(v)=\frac{{{{{\mathcal{P}}}}}_{n,m}(v)}{{\sum }_{{v}^{{\prime} }\in {{{\mathcal{V}}}}}{{{{\mathcal{P}}}}}_{n,m}({v}^{{\prime} })},$$

where ${{{{\mathcal{P}}}}}_{n,m}(v)$ is the method-specific spectral estimate for the *m*-th generation of sample *n*, and ${{{\mathcal{V}}}}=\{{v}_{\min },\ldots,{v}_{\max }\}$ denotes the analysis band defined by the lower and upper frequency limits. The resulting distribution *q*_*n*,*m*_(*v*) captures the relative spectral composition of each signal and provides a principled representation of waveform identity. In this approach, phase information is not considered, as the objective, which is to quantify the fidelity of amplitude separation from waveform shape, requires only amplitude-wise random variables for probabilistic modeling.

We employed three complementary estimators for ${{{{\mathcal{P}}}}}_{n,m}(v)$ to avoid reliance on a single representation and to provide multiple perspectives on waveform structure: the FFT, which provides a canonical and computationally efficient estimate of global spectral power by decomposing the waveform into stationary frequency components^24^; the WSE, which averages overlapping short-time spectra to reduce variance and capture non-stationary spectral dynamics^35^; and the DCT, which projects the waveform onto energy-compacting cosine bases, retaining only the most informative coefficients to yield a concise yet shape-preserving spectral representation^36^.

#### Foundation of amplitude controllability

A principled assessment of amplitude controllability requires quantifying structured spectral transitions induced by ordered modulation inputs. To this end, we introduce a permutation-based representation that captures localized regularities in PSD sequences, revealing how the ordering of the cascaded filter coefficients (i.e., conditional inputs) reflects the frequency-domain organization of synthesized signals.

For each sample *n*, we compute PSD_*n*,*v*,*m*_ across generations *m* = 1, …, *M* at each frequency bin *v*. These sequences are divided into overlapping windows of length *W*, each capturing a short-term spectral trajectory (Fig. 2a). The ordinal pattern of each window is determined by the relative ordering of its elements, taking values in the set of all possible permutations ${{{\mathcal{S}}}}=\{{\pi }_{1},{\pi }_{2},\ldots,{\pi }_{W!}\}$.

Let Π_*n*_(*s*, *v*) denote the number of occurrences of ordinal pattern $s\in {{{\mathcal{S}}}}$ at frequency *v* for sample *n*. Aggregating across frequencies, the permutation density defines a probability mass function over *s*:

7 $${q}_{n}(s)=\frac{{\sum }_{v}{\Pi }_{n}(s,v)}{{\sum }_{{s}^{{\prime} }\in {{{\mathcal{S}}}}}{\sum }_{v}{\Pi }_{n}({s}^{{\prime} },v)}.$$

This distribution *q*_*n*_(*s*) characterizes localized spectral orderings and enables evaluation of amplitude modulation precision induced by conditional inputs.

#### Metrics

We formalize five metrics, four of which are designed on the basis of CMI.

First, waveform shape factorization is defined as $I(V;\widehat{{{{\bf{Z}}}}}| {{{\bf{Z}}}})$, which quantifies the extent to which an activated latent variable $\widehat{{{{\bf{Z}}}}}$ captures shape variation not explained by the full latent vector **Z**. Higher values indicate that individual latent dimensions contribute to generating distinct shape patterns, while lower values suggest that multiple dimensions encode overlapping or entangled features, resulting in less differentiated shape generation. Formally,

8 $$I(V;\widehat{{{{\bf{Z}}}}}| {{{\bf{Z}}}})={{\mathbb{E}}}_{q(\widehat{{{{\bf{z}}}}},{{{\bf{z}}}})}[{{\rm{KL}}}(\mathit{q}(\mathit{v}| \widehat{{{{\bf{z}}}}})\,\parallel \,\mathit{q}(\mathit{v}| {{{\bf{z}}}}))].$$

Second, shape preservation quantifies the extent to which the spectral structure *V* manifested by $\widehat{{{{\bf{Z}}}}}$ is preserved under value-ordered conditional inputs $\widehat{{{{\boldsymbol{\Theta }}}}}$. Quantitatively, this metric reflects the degree of shape distortion induced by conditioning, denoted as $I(V;\widehat{{{{\boldsymbol{\Theta }}}}}| \widehat{{{{\bf{Z}}}}})$. Low values indicate that the conditioning input preserves the shape representation encoded in $\widehat{{{{\bf{Z}}}}}$, whereas high values suggest that conditioning disrupts or overrides this shape consistency. Formally,

9 $$I(V;\widehat{{{{\boldsymbol{\Theta }}}}}| \widehat{{{{\bf{Z}}}}})={{\mathbb{E}}}_{q(\widehat{{{{\mathbf{\uptheta }}}}},\widehat{{{{\bf{z}}}}})}\left[{{\rm{KL}}}(\mathit{q}(\mathit{v}| \widehat{{{{\mathbf{\uptheta }}}}},\widehat{{{{\bf{z}}}}})\,\parallel \,\mathit{q}(\mathit{v}| \widehat{{{{\bf{z}}}}}))\right].$$

Third, amplitude controllability is quantified by $I(S;\underline{\widehat{{{{\boldsymbol{\Theta }}}}}}| \underline{\widehat{{{{\bf{Z}}}}}})$, which measures the extent to which the conditional input drives systematic variations in the spectral pattern. Here, *S* denotes the ordinal permutation pattern over PSD windows. Higher values indicate that the conditional input exerts precise and consistent control over spectral structure, whereas lower values suggest weak or stochastic responses, reflecting limited influence of the conditioning input. Mathematically,

10 $$I(S;\underline{\widehat{{{{\boldsymbol{\Theta }}}}}}| \underline{\widehat{{{{\bf{Z}}}}}})={{\mathbb{E}}}_{q(\underline{\widehat{{{{\mathbf{\uptheta }}}}}},\underline{\widehat{{{{\bf{z}}}}}})}\left[{{\rm{KL}}}(\mathit{q}(\mathit{s}| \underline{\widehat{{{{\mathbf{\uptheta }}}}}},\underline{\widehat{{{{\bf{z}}}}}})\,\parallel \,\mathit{q}(\mathit{s}| \underline{\widehat{{{{\bf{z}}}}}}))\right],$$

where $\underline{\widehat{{{{\bf{z}}}}}}={\{{\widehat{{{{\bf{z}}}}}}_{m}\}}_{m=1}^{M}$ and $\underline{\widehat{{{{\mathbf{\uptheta }}}}}}={\{{\widehat{{{{\mathbf{\uptheta }}}}}}_{m}\}}_{m=1}^{M}$ denote sets of distinct conditional realizations. Additional details are provided in the supplementary information.

Fourth, spectral similarity is characterized by the filtering quality index (FQI), which identifies a subset of synthesized signals whose spectral profiles most closely resemble those of the ground-truth data. For each generated signal, its normalized PSD is compared with a reference batch of real signals using the KL divergence, and the minimum divergence value, corresponding to the best spectral match, is used to represent fidelity. Generated signals with divergence values below a threshold (*τ* = 1) are retained for evaluation. This process yields a structurally informative set of latent and conditioning configurations $(\vec{{{{\bf{z}}}}},\vec{{{{\mathbf{\uptheta }}}}})$. The final FQI score is then computed as the symmetric KL divergence between the PSD of real signals, *P*(*v*∣**y**), and that of the filtered synthetic set, $Q(v| \vec{{{{\bf{z}}}}},\vec{{{{\mathbf{\uptheta }}}}})$:

11 $${{{\rm{FQI}}}}=\frac{1}{2}[{{{\rm{KL}}}}({P}\,\parallel \,Q)+{{{\rm{KL}}}}({Q}\,\parallel \,P)].$$

A full mathematical description of the filtering procedure, along with implementation details, is provided in the supplementary information.

Fifth, reconstruction accuracy is determined by the coefficient of determination (*R*^2^) between each synthesized signal and its corresponding ground-truth reference. It is defined as

12 $${R}^{2}=1-\frac{\frac{1}{NT}{\sum }_{n=1}^{N}{\sum }_{t=1}^{T}{({\widehat{{{{\bf{y}}}}}}_{n,t}-{{{{\bf{y}}}}}_{n,t})}^{2}}{\frac{1}{NT}{\sum }_{n=1}^{N}{\sum }_{t=1}^{T}{({{{{\bf{y}}}}}_{n,t}-\overline{{{{\bf{y}}}}})}^{2}},$$

where ${\widehat{{{{\bf{y}}}}}}_{n,t}$ and **y**_*n*,*t*_ denote the synthesized and true signal values for sample *n* at time *t*, respectively, and $\overline{{{{\bf{y}}}}}$ is the mean of the true signals.

#### Metric normalization

To enable direct comparison across heterogeneous evaluation criteria, all metrics were normalized to the unit interval [0, 1], except for reconstruction accuracy (*R*^2^), which is inherently bounded within this range. Specifically, the CMI-based metrics were normalized using Linfoot’s informational correlation^51^, defined as ${{{\mathcal{T}}}}(I)=\sqrt{1-\exp (-2I)}$. For interpretational consistency, shape distortion and FQI were inverted as $1-{{{\mathcal{T}}}}(I)$, expressed as shape preservation and spectral similarity, respectively, ensuring that higher values uniformly indicate superior performance across all metrics.

#### Integrated evaluation: ISCORE

Generative models require evaluation across multiple criteria, as individual metrics capture only partial and sometimes conflicting aspects of performance. Accordingly, we introduce ISCORE, which consolidates the five normalized metrics using AM, GM, and HM^26^. These complementary averaging schemes emphasize distinct perspectives: the AM reflects overall magnitude, the GM rewards balanced performance, and the HM penalizes weaknesses in any single criterion^26,27^. This multi-perspective formulation prevents any single metric from dominating and provides a robust, balanced assessment of both decoupling quality and generative fidelity.

Metric inclusion within ISCORE is tailored to each model architecture. For models with factorized latent representations (e.g., VABAM and VAE-based models), all five metrics are included, while for models with structural limitations in latent factorization (e.g., WaveNet and diffusion models), the shape factorization metric is excluded.

## Supplementary information

Supplementary Information

Transparent Peer Review file

## Data Availability

The raw MIMIC-III Waveform Database used in this study is publicly available from PhysioNet under (10.13026/c2607m) in accordance with the Open Database License (ODbL). The processed MIMIC-III Waveform dataset generated in this study has been deposited in Zenodo under 10.5281/zenodo.19354579^52^. The VitalDB dataset used in this study is publicly available via PhysioNet under 10.13026/czw8-9p62. The data were accessed directly from the official VitalDB website (https://vitaldb.net) using the provider’s Python library. Under the original provider’s terms of use, the data are not redistributed by the authors. Access requires registration and acceptance of the provider’s terms (https://vitaldb.net/registration-agreement/). Data use is restricted to research and development purposes and prohibits disclosure to anyone not employed by the user’s organization without the provider’s consent. The agreement remains valid for five years unless otherwise specified and may be automatically extended. To support reproducibility and testing of the VABAM pipeline, a small synthetic demo dataset generated using the NeuroKit2 library^53^ and the script for generating a larger synthetic dataset are publicly available on GitHub (https://github.com/JunetaeKim/VABAM-official) and archived on Zenodo under 10.5281/zenodo.19351273^54^.

## Code availability

The source code is publicly available at: (https://github.com/JunetaeKim/VABAM-official) and archived at (10.5281/zenodo.19351272^54^).
