# Supplementary material for: Shape and amplitude decoupling in pulsatile physiological signal synthesis and its evaluation
Source: Nat Commun. 2026 Apr 29;17:5876. doi: 10.1038/s41467-026-72299-7 (PMC13333840; doi:10.1038/s41467-026-72299-7)
Supplement: Supplementary file 1 — Supplementary Information [file 41467_2026_72299_MOESM1_ESM.pdf]

Supplementary Information

Shape and Amplitude Decoupling in Pulsatile Physiological Signal Synthesis and Its Evaluation

Junetae Kim[1,2,*†], Kyoungsuk Park[1], Lei Chen[1], and Kyunglim Kim[3,*†]

[1]Graduate School of Cancer Science and Policy, National Cancer Center,
323 Ilsan-ro, Ilsandong-gu, Goyang-si, 10408, Gyeonggi-do, Republic of Korea

[2]Healthcare AI Team, National Cancer Center,
323 Ilsan-ro, Ilsandong-gu, Goyang-si, 10408, Gyeonggi-do, Republic of Korea

[3]Samsung Research, 56 Seongchon-gil, Seocho-gu, Seoul, 06765, Republic of Korea

Emails: lyjune0070@gmail.com (J.K.), bluemk00@gmail.com (K.P.),
cherishleon01@gmail.com (L.C.), eugeniakkim@gmail.com (K.K.)

[*]Corresponding authors
[†]These authors contributed equally to this work.

Contents

I Derivation Details of Evidence Lower Bound (ELBO) 3

II Details in Evidence Lower Bound Terms 3
II-A Reconstruction of Raw Signals . . . 3
II-B Reconstruction of Feature Signals . . . 4
II-C Variational Inference for $p(\mathbf{z})$ . . . 4
II-D Variational Inference for $p(\boldsymbol{\theta})$ . . . 4

III Mutual Information-Based Structural Metrics 6
III-A Preliminary . . . 6
III-A1 Dimensional isolation in $\mathbf{z}$ . . . 6
III-A2 Power Spectral Density Characterization . . . 6
III-A3 Permutation Density in Power Spectral Density . . . 7
III-B Assumptions . . . 7
III-C Waveform Shape Factorization $I(V;\hat{\mathbf{Z}}|\mathbf{Z})$ . . . 8
III-D Waveform Shape Preservation $I(V;\hat{\boldsymbol{\Theta}}|\hat{\mathbf{Z}})$ . . . 9
III-E Amplitude Modulation Controllability $I(S;\underline{\hat{\boldsymbol{\Theta}}} \mid \underline{\hat{\mathbf{Z}}})$ . . . 10

IV Fidelity Metrics 12
IV-A Operational Details of Filtering Quality Index . . . 12

V Realization of the VABAM Architecture 13
V-A Training Architecture . . . 13
V-B Synthesis Architecture . . . 14

VI Comprehensive Benchmark Comparison Results 15

VII Comprehensive Ablation Study Results 18

VIII Comprehensive Sensitivity Analysis Results 21

Supplementary References 24

I. Derivation Details of Evidence Lower Bound (ELBO)

This supplementary material provides a step-by-step derivation of the Evidence Lower Bound (ELBO) presented in the main article. Based on the assumptions underlying the generative process in VABAM, the joint probability distribution can be expressed as:

$$p(\mathbf{y}, \mathbf{x}, \mathbf{z}, \boldsymbol{\theta}) = p(\mathbf{y} \mid \mathbf{x})\, p(\mathbf{x} \mid \mathbf{z}, \boldsymbol{\theta})\, p(\mathbf{z})\, p(\boldsymbol{\theta}). \tag{S1}$$

The marginal likelihood $p(\mathbf{y})$ is derived by integration over variables $\mathbf{x}$, $\mathbf{z}$, and $\boldsymbol{\theta}$:

$$p(\mathbf{y}) = \iiint_D p(\mathbf{y}, \mathbf{x}, \mathbf{z}, \boldsymbol{\theta})\, d\mathbf{x}\, d\mathbf{z}\, d\boldsymbol{\theta}. \tag{S2}$$

To enable variational inference, we introduce a distribution $q(\mathbf{x}, \mathbf{z}, \boldsymbol{\theta} \mid \mathbf{y})$ that approximates the true posterior distribution $p(\mathbf{x}, \mathbf{z}, \boldsymbol{\theta} \mid \mathbf{y})$. Consequently, we get

$$p(\mathbf{y}) = \iiint_D \frac{p(\mathbf{y}, \mathbf{x}, \mathbf{z}, \boldsymbol{\theta})\, q(\mathbf{x}, \mathbf{z}, \boldsymbol{\theta} \mid \mathbf{y})}{q(\mathbf{x}, \mathbf{z}, \boldsymbol{\theta} \mid \mathbf{y})}\, d\mathbf{x}\, d\mathbf{z}\, d\boldsymbol{\theta}. \tag{S3}$$

Applying Jensen's inequality to the integral, we derive the evidence lower bound (ELBO) as:

$$\log p(\mathbf{y}) \geq \mathbb{E}_{q(\mathbf{x}, \mathbf{z}, \boldsymbol{\theta} \mid \mathbf{y})}\left[\log \frac{p(\mathbf{y}, \mathbf{x}, \mathbf{z}, \boldsymbol{\theta})}{q(\mathbf{x}, \mathbf{z}, \boldsymbol{\theta} \mid \mathbf{y})}\right] = \mathrm{ELBO}(\mathbf{y}). \tag{S4}$$

Based on our generative process, we assume that the distribution $q(\mathbf{x}, \mathbf{z}, \boldsymbol{\theta} \mid \mathbf{y})$ can be factorized as:

$$q(\mathbf{x}, \mathbf{z}, \boldsymbol{\theta} \mid \mathbf{y}) = q(\mathbf{x} \mid \mathbf{y}, \boldsymbol{\theta})\, q(\mathbf{z}, \boldsymbol{\theta} \mid \mathbf{y}), \tag{S5}$$

where $q(\mathbf{x} \mid \mathbf{y}, \boldsymbol{\theta})$ is related to the feature extractor and $q(\mathbf{z}, \boldsymbol{\theta} \mid \mathbf{y})$ is associated with both the encoder and sampler, indicating that feature signals $\mathbf{x}$ are conditioned on both the cascaded filter coefficients $\boldsymbol{\theta}$ and the raw signal $\mathbf{y}$.

The ELBO is then expressed as:

$$\begin{aligned}\mathrm{ELBO}(\mathbf{y}) = \mathbb{E}_{q(\mathbf{x}, \mathbf{z}, \boldsymbol{\theta} \mid \mathbf{y})}\big[&\log p(\mathbf{y} \mid \mathbf{x}) + \log p(\mathbf{x} \mid \mathbf{z}, \boldsymbol{\theta}) + \log p(\mathbf{z}) \\ &+ \log p(\boldsymbol{\theta}) - \log q(\mathbf{x} \mid \mathbf{y}, \boldsymbol{\theta}) - \log q(\mathbf{z} \mid \mathbf{y}) - \log q(\boldsymbol{\theta} \mid \mathbf{y})\big].\end{aligned}$$

The final objective function is defined as:

$$J(\Phi; \mathbf{y}, \mathbf{x}, \mathbf{z}, \boldsymbol{\theta}) = \mathbb{E}_{q(\mathbf{x}, \mathbf{z}, \boldsymbol{\theta} | \mathbf{y})}\left[-\log p(\mathbf{y}|\mathbf{x}) - \log \frac{p(\mathbf{x}|\mathbf{z}, \boldsymbol{\theta})}{q(\mathbf{x}|\mathbf{y}, \boldsymbol{\theta})} + \log \frac{q(\mathbf{z}|\mathbf{y})}{p(\mathbf{z})} + \log \frac{q(\boldsymbol{\theta}|\mathbf{y})}{p(\boldsymbol{\theta})}\right], \tag{S6}$$

where Φ represents all trainable network weights to be optimized.

II. Details in Evidence Lower Bound Terms

A. Reconstruction of Raw Signals

$$\mathbb{E}_{q(\mathbf{x}, \mathbf{z}, \boldsymbol{\theta} | \mathbf{y})}[\log p(\mathbf{y}|\mathbf{x})] = \int_{\mathbf{x}} \int_{\mathbf{z}} \int_{\boldsymbol{\theta}} \log p(\mathbf{y}|\mathbf{x})\, q(\mathbf{x}, \mathbf{z}, \boldsymbol{\theta}|\mathbf{y})\, d\boldsymbol{\theta}\, d\mathbf{z}\, d\mathbf{x} \tag{S7}$$

$$\approx \frac{1}{L} \sum_{l=1}^{L} \log p(\mathbf{y}|\mathbf{x}^{(l)}), \tag{S8}$$

where $\mathbf{x} \in \{\mathbf{x}_{2^\lambda - 1}, \mathbf{x}_{2^\lambda}, \ldots, \mathbf{x}_{2^{\lambda+1} - 3}, \mathbf{x}_{2^{\lambda+1} - 2}\}$.

Empirically, the following equation is applied during batch training, incorporating a weighting factor denoted as $\psi_{\mathbf{y}}$ for the loss term:

$$J(\Phi_{\mathbf{y}}; \mathbf{y}) = \psi_{\mathbf{y}} \left(\frac{1}{|B|} \sum_{b \in B} \left(\frac{1}{|D|} \sum_{d \in D} (\mathbf{y}_{b,d} - g_{\mathbf{y}}(\mathbf{x}_{b,d}))^2\right)\right), \tag{S9}$$

where b and d represent the batch index and a sample index within the batch, respectively.

B. Reconstruction of Feature Signals

$$\mathbb{E}_{q(\mathbf{z},\theta|\mathbf{y})q(\mathbf{x}|\mathbf{y},\theta)}\left[\log\frac{p(\mathbf{x}|\mathbf{z},\theta)}{q(\mathbf{x}|\mathbf{y},\theta)}\right]=\int_{\mathbf{z}}\int_{\theta}\left(\int_{\mathbf{x}}\log\frac{p(\mathbf{x}|\mathbf{z},\theta)}{q(\mathbf{x}|\mathbf{y},\theta)}q(\mathbf{x}|\mathbf{y},\theta)\,d\mathbf{x}\right)q(\mathbf{z},\theta|\mathbf{y})\,d\theta\,d\mathbf{z} \tag{S10}$$

$$=\int_{\mathbf{z}}\int_{\theta}-\mathrm{KL}\Big(q(\mathbf{x}|\mathbf{y},\theta)\,||\,p(\mathbf{x}|\mathbf{z},\theta)\Big)q(\mathbf{z},\theta|\mathbf{y})\,d\theta\,d\mathbf{z} \tag{S11}$$

$$\approx-\frac{1}{L}\sum_{l=1}^{L}\mathrm{KL}\left(q(\mathbf{x}|\mathbf{y},\theta^{(l)})\,||\,p(\mathbf{x}|\mathbf{z}^{(l)},\theta^{(l)})\right), \tag{S12}$$

where $\mathbf{z}^{(l)}\sim\mathcal{N}(0,1)$ and $\theta^{(l)}\sim\mathrm{Bernoulli}(0.5)$. $\mathrm{KL}(\cdot\,\|\,\cdot)$ denotes the Kullback–Leibler divergence.

In practice, the following equation is used during batch training, incorporating a weighting factor $\psi_{\mathbf{x}}$ for the loss term:

$$J(\Phi_{\mathbf{x}};\mathbf{x})=\psi_{\mathbf{x}}\left(\frac{1}{|B|}\sum_{b\in B}\left(\frac{1}{|D|}\sum_{d\in D}\left(g_{\mathbf{x}}(\mathbf{y}_{b,d},\theta_{b,d})-g_{\mathbf{x}'}(\mathbf{z}_{b,d},\theta_{b,d})\right)^2\right)\right), \tag{S13}$$

where $g_{\mathbf{x}}(\cdot)$ and $g_{\mathbf{x}'}(\cdot)$ denote the feature extractor and feature generator, respectively.

C. Variational Inference for $p(\mathbf{z})$

$$\mathbb{E}_{q(\mathbf{z}|\mathbf{y})q(\theta|\mathbf{y})q(\mathbf{x}|\mathbf{y},\theta)}\left[\log\frac{q(\mathbf{z}|\mathbf{y})}{p(\mathbf{z})}\right]=\int_{\mathbf{z}}\log\frac{q(\mathbf{z}|\mathbf{y})}{p(\mathbf{z})}q(\mathbf{z}|\mathbf{y})\,d\mathbf{z} \tag{S14}$$

$$=\mathrm{KL}(q(\mathbf{z}|\mathbf{y})\,||\,p(\mathbf{z})). \tag{S15}$$

We employed the KL divergence to regularize the latent variable $\mathbf{z}$ within the standard VAE framework [1], as shown below:

$$\mathrm{KL}(q(\mathbf{z}|\mathbf{y})\,||\,p(\mathbf{z}))=\frac{1}{N}\sum_{n=1}^{N}\left(\frac{1}{2}\sum_{j=1}^{J}\left(\mu^2_{\mathbf{z}_{n,j}}+\sigma^2_{\mathbf{z}_{n,j}}-1-\log\sigma^2_{\mathbf{z}_{n,j}}\right)\right). \tag{S16}$$

Here, $q(\mathbf{z}|\mathbf{y})$ represents the approximate posterior distribution of the latent variables, regularized to follow a Gaussian distribution. The z-sampler $g_{\mathbf{z}}(\cdot)$ in VABAM generates samples from $q(\mathbf{z}|\mathbf{y})\approx\mathcal{N}(0,1)$. The prior distribution $p(\mathbf{z})$ is assumed to be a standard normal distribution, $p(\mathbf{z})=\mathcal{N}(0,1)$.

For batch-level implementation, the subsequent equation is employed:

$$J(\Phi_{\mathbf{z}};\mathbf{z})=\psi_{\mathbf{z}}\left(\left|\frac{1}{|B|}\sum_{b\in B}\left(\frac{1}{2|D|}\sum_{d\in D}\sum_{j=1}^{J}\left(\mu^2_{\mathbf{z}_{b,d,j}}+\sigma^2_{\mathbf{z}_{b,d,j}}-1-\log\sigma^2_{\mathbf{z}_{b,d,j}}\right)\right)-\xi_{\mathbf{z}}\right|\right), \tag{S17}$$

where $\psi_{\mathbf{z}}$ denotes the weight assigned to this loss term and $\xi_{\mathbf{z}}$ signifies the capacity term [2].

D. Variational Inference for $p(\theta)$

$$\mathbb{E}_{q(\mathbf{z}|\mathbf{y})q(\theta|\mathbf{y})q(\mathbf{x}|\mathbf{y},\theta)}\left[\log\frac{q(\theta|\mathbf{y})}{p(\theta)}\right]=\int_{\theta}\log\frac{q(\theta|\mathbf{y})}{p(\theta)}q(\theta|\mathbf{y})\,d\theta \tag{S18}$$

$$=\mathrm{KL}(q(\theta|\mathbf{y})\,||\,p(\theta)). \tag{S19}$$

Here, $q(\theta|\mathbf{y})$ represents the approximate posterior distribution of θ, while $p(\theta)$ denotes its prior distribution, which is assumed to be uniform. To enable the reparameterization trick, this uniform distribution is approximated using a Bernoulli distribution with a mean of 0.5 [3], [4]. The corresponding KL divergence is expressed as:

$$\mathrm{KL}(q(\theta|\mathbf{y})\,||\,p(\theta))=\mathrm{KL}(q(\theta|\mathbf{y})\,||\,\mathrm{Uniform}(0,1)) \tag{S20}$$

$$\approx\mathrm{KL}(q(\theta|\mathbf{y})\,||\,\mathrm{Bern}(\mu_{\theta}=0.5)) \tag{S21}$$

$$=\frac{1}{N}\sum_{n=1}^{N}\left(\frac{1}{K}\sum_{k=1}^{K}\sum_{c\in\{0,1\}}\left(\mu^c_{\theta_{n,k}}(1-\mu_{\theta_{n,k}})^{1-c}\ln\frac{\mu^c_{\theta_{n,k}}(1-\mu_{\theta_{n,k}})^{1-c}}{0.5^c0.5^{1-c}}\right)\right), \tag{S22}$$

where $K=\sum_{i=1}^{\lambda} 2^i$ denotes the total number of cascaded filter coefficients.

Empirically, the following equation is implemented at the batch level:

$$J(\Phi_{\theta};\theta)=\psi_{\theta}\left(\left|\frac{1}{|B|}\sum_{b\in B}\left(\frac{1}{K|D|}\sum_{d\in D}\sum_{k=1}^{K}\sum_{c\in\{0,1\}}\left(\mu_{\theta_{b,d,k}}^{c}(1-\mu_{\theta_{b,d,k}})^{1-c}\ln\frac{\mu_{\theta_{b,d,k}}^{c}(1-\mu_{\theta_{b,d,k}})^{1-c}}{0.5^{c}0.5^{1-c}}\right)\right)-\xi_{\theta}\right|\right), \tag{S23}$$

where ψ_{θ} and ξ_{θ} represent a weight for this term and a capacity term [2], respectively.

III. Mutual Information-Based Structural Metrics

In this section, we present the derivations of the conditional mutual information (CMI)-based metrics, providing both analytical expressions and empirical estimations. For implementation, we recommend using the empirical formulation owing to its greater practical applicability.

A. Preliminary

1) Dimensional isolation in $\mathbf{z}$

We introduce a specialized approach for evaluating disentanglement within $\mathbf{z}$, designed to isolate a randomly selected dimension of the latent space. To this end, we define a set $\mathbf{z} = \{\mathbf{z}_j\}_{j=1}^{J}$, where $\mathbf{z}_j$ follows a normal distribution, $\mathbf{z}_j \sim \mathcal{N}(\mu_j = 0, \sigma_j^2)$. Given the set $\mathbf{z}$, we introduce a new set $\hat{\mathbf{z}} = \{\hat{\mathbf{z}}_j\}_{j=1}^{J}$, where each element $\hat{\mathbf{z}}_j$ is defined as

$$\hat{\mathbf{z}}_j = \begin{cases} \mathbf{z}_{j^*}, & \text{if } j = j^* \\ \mathbb{E}_q[\mathbf{z}_j|\mathbf{y}], & \text{if } j \neq j^*. \end{cases} \tag{S24}$$

Here, j is an index within the set $\{1, 2, \ldots, J\}$ and j^* is an index selected uniformly at random from the same range, with probability $p(j = j^*) = \frac{1}{J}$. Considering the expected value $\mathbb{E}_q[\mathbf{z}_j|\mathbf{y}]$, expressed as

$$\mathbb{E}_q[\mathbf{z}_j|\mathbf{y}] \approx \frac{1}{M} \sum_{m=1}^{M} \mathbf{z}_j^{(m)} \approx 0, \tag{S25}$$

in the set $\hat{\mathbf{z}}$, every element except for $\mathbf{z}_{j^*}$ is deterministically set to the Gaussian mean of zero for computational efficiency in this study. In contrast, the $\mathbf{z}_{j^*}$ retains its original value from the set $\mathbf{z}$, thereby achieving its isolation within the latent space.

2) Power Spectral Density Characterization

For each synthesized signal, spectral information was extracted from its frequency-domain representation. We define a method-specific spectral estimate $\mathcal{P}_{n,m}(v)$ at frequency bin v for the m-th realization of sample n, and normalize it as

$$q_{n,m}(v) = \frac{\mathcal{P}_{n,m}(v)}{\sum_{v' \in \mathcal{V}} \mathcal{P}_{n,m}(v')}, \tag{S26}$$

where $\mathcal{V} = \{v_{\min}, \ldots, v_{\max}\}$ specifies the analysis band of interest, with $v_{\min} = 1$ and $v_{\max} = 100$ in this study. The distribution $q_{n,m}(v)$ was estimated using three complementary methods, each capturing distinct aspects of the waveform's spectral composition.

a) Fast Fourier Transform (FFT).

When the classical FFT was applied, the spectral estimate was taken as

$$\mathcal{P}_{n,m}^{\mathrm{FFT}}(v) = \tfrac{1}{T} \left|X_{n,m}(v)\right|^2, \tag{S27}$$

where T is the signal length in samples and $X_{n,m}(v)$ denotes the discrete Fourier transform coefficient [5].

b) Discrete Cosine Transform Type-II (DCT).

For the DCT, coefficients $C_{n,m}(v)$ were obtained by applying the transform to the raw time-domain waveform, thereby mapping temporal samples onto cosine basis coefficients [6]. To promote sparsity, only the k largest coefficients were retained using a hard-thresholding operator $\mathcal{H}_k$, yielding

$$\mathcal{P}_{n,m}^{\mathrm{DCT}}(v) = \left|\mathcal{H}_k\{C_{n,m}\}(v)\right|^2. \tag{S28}$$

We set k to twice the bandwidth, constrained between 128 and the signal length T. This representation provides a computationally efficient means of emphasizing dominant frequency components while discarding minor contributions.

c) Welch-based Evolution Analysis.

In this approach, the signal is divided into overlapping segments, and the corresponding periodograms are averaged to reduce estimator variance [7]. For T_s segments, the estimate is written as

$$\mathcal{P}_{n,m}^{\mathrm{Welch\text{-}evo}}(v) = \tfrac{1}{T_s} \sum_{t=1}^{T_s} \left|\mathcal{Z}_{n,m}(v,t)\right|^2, \tag{S29}$$

with $\mathcal{Z}_{n,m}(v,t)$ the short-time Fourier transform coefficient at time index t. This variant highlights how spectral content evolves while suppressing noise-driven fluctuations.

3) Permutation Density in Power Spectral Density

We introduce a novel metric to evaluate the controllability of amplitude modulation. This metric quantifies the orderliness of synthesized signals by analyzing the permutation distribution of power spectral density (PD-PSD) values. It captures the degree of structured variation in spectral content induced by ordered conditioning inputs, thereby reflecting how effectively the model responds to amplitude-modulation control. We formalize this metric through the following procedure:

① *Defining PSD series:* We begin by recalling the definition of PSD as introduced in the main manuscript:

$$\text{PSD} = \{\text{PSD}_{n,v,1}, \text{PSD}_{n,v,2}, \ldots, \text{PSD}_{n,v,M}\},$$

where n, v, and M represent the sample index, the frequency index, and the generation size, respectively.

② *Constructing windowed vectors:* We proceed by constructing W-dimensional vectors for a selected window dimension $W \geq 2$ as:

$$\text{WPSD}_t = (\text{PSD}_{n,v,t}, \text{PSD}_{n,v,(t+1)}, \ldots, \text{PSD}_{n,v,(t+W-1)}),$$

where $t \in \{1, \ldots, M - W + 1\}$.

③ *Generating permutation sets:* We consider the set of all $W!$ unique ordinal permutations. Each permutation π_s represents the s-th element in this set, where $s \in \{1, \ldots, W!\}$. These permutations represent all possible orderings of the PSD values within WPSD_t.

④ *Tallying occurrences:* We tally occurrences of each permutation π_s as $\Pi_n(s, v)$ by associating every WPSD_t with its respective permutation.

⑤ *Defining permutation density:* Finally, we define the permutation density by summing $\Pi_n(s, v)$ over all frequency indices v:

$$q_n(s) = \frac{\sum_{v=1}^{|\mathcal{V}|} \Pi_n(s, v)}{\sum_{v=1}^{|\mathcal{V}|} \sum_{s=1}^{W!} \Pi_n(s, v)}. \tag{S30}$$

B. Assumptions

For estimating CMI-based metrics, we made some assumptions.

- **Uniform Distribution of Data Points:** Each test dataset point is uniquely indexed by an integer and associated with a uniformly distributed random variable [8]. Consequently, for a randomly selected instance $\mathbf{y}_n$, where $n \sim \mathcal{U}(1, N)$, its probability is defined as $q(\mathbf{y}_n) = q(n) = \frac{1}{N}$.
- **Uniform Distribution of Frequency Index:** Similarly, the probability of observing the random variable v, representing the frequency index, follows a uniform distribution defined as $q(v) = \frac{1}{|\mathcal{V}|}$.
- **Sampling via VABAM:** We sample the latent variables from the VABAM sampler according to their $\mathbf{y}$-conditioned posteriors, $\mathbf{z} \sim q(\mathbf{z} \mid \mathbf{y})$ and $\boldsymbol{\theta} \sim q(\boldsymbol{\theta} \mid \mathbf{y})$, with priors $p(\mathbf{z}) = \mathcal{N}(0, 1)$ and $p(\boldsymbol{\theta}) = \mathcal{U}(0, 1)$. This approach leverages the dependency structure of VABAM, where $\mathbf{y}$ affects the latent variables $\mathbf{z}$ and $\boldsymbol{\theta}$, and these latent variables in turn determine v and s. Critically, once $\mathbf{z}$ and $\boldsymbol{\theta}$ are sampled from their $\mathbf{y}$-conditioned distributions, the generation of v and s depends only on these latent variables and not directly on $\mathbf{y}$. As $\mathbf{z}$ and $\boldsymbol{\theta}$ already summarize all information from $\mathbf{y}$ that is relevant to v and s, this yields the conditional independence $v, s \perp \mathbf{y} \mid \mathbf{z}, \boldsymbol{\theta}$, expressed by the equivalences $q(v|\mathbf{z}, \boldsymbol{\theta}) = q(v|\mathbf{z}, \boldsymbol{\theta}, \mathbf{y})$ and $q(s|\mathbf{z}, \boldsymbol{\theta}) = q(s|\mathbf{z}, \boldsymbol{\theta}, \mathbf{y})$.

Ultimately, these assumptions simplify computation and enable efficient inference, consistent with foundational practices in deep learning and variational inference.

C. Waveform Shape Factorization $I(V;\hat{\mathbf{Z}}|\mathbf{Z})$

a) Basic Analytical Derivation

$$I(V;\hat{\mathbf{Z}}|\mathbf{Z}) = \mathbb{E}_{q(v,\hat{\mathbf{z}},\mathbf{z})}\left[\log \frac{q(v,\hat{\mathbf{z}}|\mathbf{z})}{q(v|\mathbf{z})q(\hat{\mathbf{z}}|\mathbf{z})}\right] \tag{S31}$$

$$= \mathbb{E}_{q(v,\hat{\mathbf{z}},\mathbf{z})}\left[\log \frac{(q(v,\hat{\mathbf{z}},\mathbf{z})/q(\mathbf{z}))}{q(v|\mathbf{z})q(\hat{\mathbf{z}}|\mathbf{z})}\right] \tag{S32}$$

$$= \mathbb{E}_{q(v,\hat{\mathbf{z}},\mathbf{z})}\left[\log \frac{(q(v|\hat{\mathbf{z}})q(\hat{\mathbf{z}}|\mathbf{z})q(\mathbf{z})/q(\mathbf{z}))}{q(v|\mathbf{z})q(\hat{\mathbf{z}}|\mathbf{z})}\right] \tag{S33}$$

$$= \mathbb{E}_{q(v|\hat{\mathbf{z}})q(\hat{\mathbf{z}},\mathbf{z})}\left[\log \frac{q(v|\hat{\mathbf{z}})}{q(v|\mathbf{z})}\right] \tag{S34}$$

$$= \mathbb{E}_{q(\hat{\mathbf{z}},\mathbf{z})}\left[\sum_{v\in\mathcal{V}} q(v|\hat{\mathbf{z}}) \log \frac{q(v|\hat{\mathbf{z}})}{q(v|\mathbf{z})}\right]. \tag{S35}$$

b) Approximation

$$I(V;\hat{\mathbf{Z}}|\mathbf{Z}) = \int_{\hat{\mathbf{z}}}\int_{\mathbf{z}} q(\hat{\mathbf{z}},\mathbf{z})\left(\sum_{v\in\mathcal{V}} q(v|\hat{\mathbf{z}}) \log \frac{q(v|\hat{\mathbf{z}})}{q(v|\mathbf{z})}\right) d\hat{\mathbf{z}}\, d\mathbf{z} \tag{S36}$$

$$\approx \frac{1}{L}\sum_{l=1}^{L} \mathrm{KL}\left(q(v|\hat{\mathbf{z}}^{(l)}) \parallel q(v|\mathbf{z}^{(l)})\right) \tag{S37}$$

$$\approx \frac{1}{N}\sum_{n=1}^{N} \mathrm{KL}\left(q(v|\hat{\mathbf{z}}^{(n)},\mathbf{y}_n) \parallel q(v|\mathbf{z}^{(n)},\mathbf{y}_n)\right) \geq 0, \tag{S38}$$

where $q(v|\hat{\mathbf{z}}^{(n)},\mathbf{y}_n)$ and $q(v|\mathbf{z}^{(n)},\mathbf{y}_n)$ are approximated as follows:

$$q(v|\hat{\mathbf{z}}^{(n)},\mathbf{y}_n) \approx \frac{1}{M}\sum_{m=1}^{M} q(v|\hat{\mathbf{z}}^{(n)},\theta^{(n,m)},\mathbf{y}_n). \tag{S39}$$

$$q(v|\mathbf{z}^{(n)},\mathbf{y}_n) \approx \frac{1}{M}\sum_{m=1}^{M} q(v|\mathbf{z}^{(n)},\theta^{(n,m)},\mathbf{y}_n). \tag{S40}$$

c) Final Expression

$$I(V;\hat{\mathbf{Z}}|\mathbf{Z}) = \frac{1}{N}\sum_{n=1}^{N} \mathrm{KL}\left(\frac{1}{M}\sum_{m=1}^{M} q(v|\hat{\mathbf{z}}^{(n)},\theta^{(n,m)},\mathbf{y}_n) \parallel \frac{1}{M}\sum_{m=1}^{M} q(v|\mathbf{z}^{(n)},\theta^{(n,m)},\mathbf{y}_n)\right) \geq 0. \tag{S41}$$

d) Empirical Formulation

$$I^*(V;\hat{\mathbf{Z}}|\mathbf{Z}) = \frac{1}{|B|}\sum_{b\in B}\left(\frac{1}{|D|}\sum_{d\in D} \mathrm{KL}\left(\frac{1}{M}\sum_{m=1}^{M} q(v|\hat{\mathbf{z}}^{(b,d)},\theta^{(b,d,m)},\mathbf{y}_{b,d}) \parallel \frac{1}{M}\sum_{m=1}^{M} q(v|\mathbf{z}^{(b,d)},\theta^{(b,d,m)},\mathbf{y}_{b,d})\right)\right). \tag{S42}$$

e) Modification for Benchmark Models without θ

$$I^z(V;\hat{\mathbf{Z}}|\mathbf{Z}) = \frac{1}{|B|}\sum_{b\in B}\left(\frac{1}{|D|}\sum_{d\in D} \mathrm{KL}\left(q(v|\hat{\mathbf{z}}^{(b,d)},\mathbf{y}_{b,d}) \parallel q(v|\mathbf{z}^{(b,d)},\mathbf{y}_{b,d})\right)\right). \tag{S43}$$

f) Clarification of Prior Derivations

- V indicates the normalized PSD treated as a random variable.
- L and M denote the outer and inner Monte Carlo sample sizes, respectively.
- $|B|$ is the total number of batches.
- $|D|$ is the number of samples in each batch, calculated as $\frac{N}{|B|}$.
- $\mathbf{z}^{(b,d)}$ is a 4D tensor represented as $\mathbf{z}^{(b,d)} \in \mathbb{R}^{|B|\times|D|\times M\times J}$, indexed by a batch index b, a sample index in the batch d, sub-repeat index m, and latent vector dimension index j. The values of $\mathbf{z}$ are randomly sampled at the dimensions b, d, and j, but remain constant across the dimension m, denoted as $\mathbf{z}_{b,d,m,j} = \mathbf{z}_{b,d,j}\ \forall m$.
- $\theta^{(b,d,m)}$ is a 4D tensor denoted as $\theta^{(b,d,m)} \in \mathbb{R}^{|B|\times|D|\times M\times K}$, with indexing based on a batch index b, a sample index in the batch d, sub-sampling index m, and latent vector dimension index k. The values of θ are sampled randomly at dimensions b, d, m, and k.
- $q(v)$ is a 4D tensor, expressed as $q(v) \in \mathbb{R}^{|B|\times|D|\times M\times|\mathcal{V}|}$, with indices comprising a batch index b, a sample index in the batch d, sub-repeat index m, and frequency indices v treated as random variables.
- $p(v)$ is a 1D distribution vector for the normalized PSD across the frequency indices, v, in the test dataset.
- $\hat{\mathbf{z}}^{(b,d)}$ is a 4D tensor represented as $\hat{\mathbf{z}} \in \mathbb{R}^{|B|\times|D|\times M\times J}$, indexed by the batch index b, a sample index within the batch d, sub-repeat index m, and latent dimension index j. It is constructed from $\mathbf{z}^{(b,d)}$ through dimensional isolation: Initially, the values of $\mathbf{z}$ are randomly sampled along the dimensions b, d, and j, but remain constant across m, such that $\mathbf{z}_{b,d,m,j} = \mathbf{z}_{b,d,j}\ \forall m$. Subsequently, all elements except the isolated component $\mathbf{z}_{j=j^*}$, which retains its original value from $\mathbf{z}_j$, are set to zero.

D. Waveform Shape Preservation $I(V;\hat{\mathbf{\Theta}}|\hat{\mathbf{Z}})$

a) Basic Analytical Derivation

$$I(V;\hat{\mathbf{\Theta}}|\hat{\mathbf{Z}}) = \mathbb{E}_{q(v,\hat{\theta},\hat{\mathbf{z}})}\left[\log\frac{q(v,\hat{\theta}|\hat{\mathbf{z}})}{q(v|\hat{\mathbf{z}})q(\hat{\theta}|\hat{\mathbf{z}})}\right] \tag{S44}$$

$$= \mathbb{E}_{q(v,\hat{\theta},\hat{\mathbf{z}})}\left[\log\frac{\left(q(v,\hat{\theta},\hat{\mathbf{z}})/q(\hat{\mathbf{z}})\right)}{q(v|\hat{\mathbf{z}})q(\hat{\theta}|\hat{\mathbf{z}})}\right] \tag{S45}$$

$$= \mathbb{E}_{q(v,\hat{\theta},\hat{\mathbf{z}})}\left[\log\frac{\left(q(v|\hat{\theta},\hat{\mathbf{z}})q(\hat{\theta}|\hat{\mathbf{z}})q(\hat{\mathbf{z}})/q(\hat{\mathbf{z}})\right)}{q(v|\hat{\mathbf{z}})q(\hat{\theta}|\hat{\mathbf{z}})}\right] \tag{S46}$$

$$= \mathbb{E}_{q(v|\hat{\theta},\hat{\mathbf{z}})q(\hat{\theta},\hat{\mathbf{z}})}\left[\log\frac{q(v|\hat{\theta},\hat{\mathbf{z}})}{q(v|\hat{\mathbf{z}})}\right] \tag{S47}$$

$$= \mathbb{E}_{q(\hat{\theta},\hat{\mathbf{z}})}\left[\sum_{v\in\mathcal{V}} q(v|\hat{\theta},\hat{\mathbf{z}})\log\frac{q(v|\hat{\theta},\hat{\mathbf{z}})}{q(v|\hat{\mathbf{z}})}\right]. \tag{S48}$$

b) Approximation

$$I(V;\hat{\mathbf{\Theta}}|\hat{\mathbf{Z}}) = \int_{\hat{\mathbf{z}}}\int_{\hat{\theta}} q(\hat{\theta},\hat{\mathbf{z}})\left(\sum_{v\in\mathcal{V}} q(v|\hat{\theta},\hat{\mathbf{z}})\log\frac{q(v|\hat{\theta},\hat{\mathbf{z}})}{q(v|\hat{\mathbf{z}})}\right)d\hat{\theta}\,d\hat{\mathbf{z}} \tag{S49}$$

$$\approx \frac{1}{N}\sum_{n=1}^{N}\mathrm{KL}\left(q(v|\hat{\theta}^{(n)},\hat{\mathbf{z}}^{(n)},\mathbf{y}_n)\parallel q(v|\hat{\mathbf{z}}^{(n)},\mathbf{y}_n)\right)\geq 0, \tag{S50}$$

where $q(v|\hat{\mathbf{z}}^{(n)},\mathbf{y}_n)$ is approximated as follows:

$$q(v|\hat{\mathbf{z}}^{(n)},\mathbf{y}_n) \approx \frac{1}{M}\sum_{m=1}^{M} q(v|\hat{\mathbf{z}}^{(n)},\theta^{(n,m)},\mathbf{y}_n). \tag{S51}$$

c) Final Expression

$$I(V;\hat{\boldsymbol{\Theta}}|\hat{\mathbf{Z}}) = \frac{1}{N}\sum_{n=1}^{N}\mathrm{KL}\left(q(v|\hat{\mathbf{z}}^{(n)},\hat{\boldsymbol{\theta}}^{(n)},\mathbf{y}_n)\parallel\frac{1}{M}\sum_{m=1}^{M}q(v|\hat{\mathbf{z}}^{(n)},\boldsymbol{\theta}^{(n,m)},\mathbf{y}_n)\right). \tag{S52}$$

d) Empirical Formulation

$$I^*(V;\hat{\boldsymbol{\Theta}}|\hat{\mathbf{Z}}) = \frac{1}{|B|}\sum_{b\in B}\left(\frac{1}{|D|}\sum_{d\in D}\mathrm{KL}\left(q(v|\hat{\mathbf{z}}^{(b,d)},\hat{\boldsymbol{\theta}}^{(b,d)},\mathbf{y}_{b,d})\parallel\frac{1}{M}\sum_{m=1}^{M}q(v|\hat{\mathbf{z}}^{(b,d)},\boldsymbol{\theta}^{(b,d,m)},\mathbf{y}_{b,d})\right)\right). \tag{S53}$$

e) Clarification of Prior Derivations

- $\hat{\boldsymbol{\theta}}^{(b,d)}$ is a 3D tensor denoted as $\hat{\boldsymbol{\theta}}^{(b,d)} \in \mathbb{R}^{|B|\times|D|\times K}$, with indexing based on a batch index b, a sample index in the batch d, and a latent vector dimension index k. It is constructed from $\boldsymbol{\theta}^{(b,d)}$ by sorting: Initially, the values of $\boldsymbol{\theta}$ are sampled randomly at the dimensions b, d, and k, and then $\boldsymbol{\theta}_{b,d,k}$ is sorted along the d-dimension to yield $\hat{\boldsymbol{\theta}}_{b,d,k}$.

E. Amplitude Modulation Controllability $I(S;\underline{\hat{\boldsymbol{\Theta}}} \mid \underline{\hat{\mathbf{Z}}})$

a) Basic Analytical Derivation

$$I(S;\underline{\hat{\boldsymbol{\Theta}}} \mid \underline{\hat{\mathbf{Z}}}) = \mathbb{E}_{q(s|\underline{\hat{\boldsymbol{\theta}}},\underline{\hat{\mathbf{z}}})q(\underline{\hat{\boldsymbol{\theta}}},\underline{\hat{\mathbf{z}}})}\left[\log\frac{q(s,\underline{\hat{\boldsymbol{\theta}}}|\underline{\hat{\mathbf{z}}})}{q(s|\underline{\hat{\mathbf{z}}})q(\underline{\hat{\boldsymbol{\theta}}}|\underline{\hat{\mathbf{z}}})}\right] \tag{S54}$$

$$= \mathbb{E}_{q(\underline{\hat{\boldsymbol{\theta}}},\underline{\hat{\mathbf{z}}})}\left[\sum_{s\in\mathcal{S}}q(s|\underline{\hat{\boldsymbol{\theta}}},\underline{\hat{\mathbf{z}}})\log\frac{q(s|\underline{\hat{\boldsymbol{\theta}}},\underline{\hat{\mathbf{z}}})q(\underline{\hat{\boldsymbol{\theta}}},\underline{\hat{\mathbf{z}}})/q(\underline{\hat{\mathbf{z}}})}{q(s|\underline{\hat{\mathbf{z}}})q(\underline{\hat{\boldsymbol{\theta}}}|\underline{\hat{\mathbf{z}}})}\right] \tag{S55}$$

$$= \mathbb{E}_{q(\underline{\hat{\boldsymbol{\theta}}},\underline{\hat{\mathbf{z}}})}\left[\sum_{s\in\mathcal{S}}q(s|\underline{\hat{\boldsymbol{\theta}}},\underline{\hat{\mathbf{z}}})\log\frac{q(s|\underline{\hat{\boldsymbol{\theta}}},\underline{\hat{\mathbf{z}}})}{q(s|\underline{\hat{\mathbf{z}}})}\right]. \tag{S56}$$

b) Approximation

$$I(S;\underline{\hat{\boldsymbol{\Theta}}} \mid \underline{\hat{\mathbf{Z}}}) = \int_{\underline{\hat{\mathbf{z}}}}\int_{\underline{\hat{\boldsymbol{\theta}}}}q(\underline{\hat{\boldsymbol{\theta}}},\underline{\hat{\mathbf{z}}})\left(\sum_{s\in\mathcal{S}}q(s|\underline{\hat{\boldsymbol{\theta}}},\underline{\hat{\mathbf{z}}})\log\frac{q(s|\underline{\hat{\boldsymbol{\theta}}},\underline{\hat{\mathbf{z}}})}{q(s|\underline{\hat{\mathbf{z}}})}\right)d\underline{\hat{\boldsymbol{\theta}}}\,d\underline{\hat{\mathbf{z}}} \tag{S57}$$

$$\approx \frac{1}{N}\sum_{n=1}^{N}\mathrm{KL}\left(q(s|\underline{\hat{\boldsymbol{\theta}}}^{(n)},\underline{\hat{\mathbf{z}}}^{(n)},\mathbf{y}_n)\parallel q(s|\underline{\hat{\mathbf{z}}}^{(n)},\mathbf{y}_n)\right) \geq 0, \tag{S58}$$

where $q(s|\underline{\hat{\mathbf{z}}}^{(n)},\mathbf{y}_n)$ is approximated as follows:

$$q(s|\underline{\hat{\mathbf{z}}}^{(n)},\mathbf{y}_n) \approx \frac{1}{R}\sum_{r=1}^{R}\sum_{v\in\mathcal{V}}q(s,v|\underline{\hat{\mathbf{z}}}^{(n)},\underline{\boldsymbol{\theta}}^{(n,r)},\mathbf{y}_n). \tag{S59}$$

c) Final Expression

$$I(S;\underline{\hat{\boldsymbol{\Theta}}} \mid \underline{\hat{\mathbf{Z}}}) = \frac{1}{N}\sum_{n=1}^{N}\mathrm{KL}\left(\sum_{v\in\mathcal{V}}q(s,v|\underline{\hat{\mathbf{z}}}^{(n)},\underline{\hat{\boldsymbol{\theta}}}^{(n)},\mathbf{y}_n)\parallel\frac{1}{R}\sum_{r=1}^{R}\sum_{v\in\mathcal{V}}q(s,v|\underline{\hat{\mathbf{z}}}^{(n)},\underline{\boldsymbol{\theta}}^{(n,r)},\mathbf{y}_n)\right). \tag{S60}$$

d) Empirical Formulation

$$I^*(S;\underline{\hat{\boldsymbol{\Theta}}} \mid \underline{\hat{\mathbf{Z}}}) = \frac{1}{|B|}\sum_{b\in B}\left(\frac{1}{|D|}\sum_{d\in D}\mathrm{KL}\left(\sum_{v\in\mathcal{V}}q(s,v|\underline{\hat{\mathbf{z}}}^{(b,d)},\underline{\hat{\boldsymbol{\theta}}}^{(b,d)},\mathbf{y}_{b,d})\parallel\frac{1}{R}\sum_{r=1}^{R}\sum_{v\in\mathcal{V}}q(s,v|\underline{\hat{\mathbf{z}}}^{(b,d)},\underline{\boldsymbol{\theta}}^{(b,d,r)},\mathbf{y}_{b,d})\right)\right). \tag{S61}$$

e) Clarification of Prior Derivations

- R denotes the number of Monte Carlo samples, distinct in level from N and M, and serves as the number of data sub-partitions.
- $\underline{\boldsymbol{\theta}}^{(b,d,r)}$ is defined as $\underline{\boldsymbol{\theta}}^{(b,d,r)} = \{\boldsymbol{\theta}_{(b,d,r,1)}, \boldsymbol{\theta}_{(b,d,r,2)}, \ldots, \boldsymbol{\theta}_{(b,d,r,M)}\}$ and operated as a 5D tensor, indexed based on a batch index b, a sample index in the batch d, a data-partition indicator r, a sub-sampling index m, and a latent vector dimension index k. The values of $\underline{\boldsymbol{\theta}}$ are randomly sampled along the dimensions b, d, r, m and k.
- $q(s, v)$ is defined as a 5D tensor, $q(s, v) \in \mathbb{R}^{|B| \times |D| \times R \times |\mathcal{V}| \times |\mathcal{S}|}$, with indices comprising a batch index b, a sample index in the batch d, a data-partition indicator r, frequency indices v, and permutation indices s.
- $\hat{\underline{\mathbf{z}}}^{(b,d)}$ is defined as $\hat{\underline{\mathbf{z}}}^{(b,d)} = \{\hat{\mathbf{z}}_{(b,d,1)}, \hat{\mathbf{z}}_{(b,d,2)}, \ldots, \hat{\mathbf{z}}_{(b,d,M)}\}$ and treated as a 4D tensor. It is indexed by a batch index b, a sample index within the batch d, a sub-repeat index m, and a latent dimension index j. Initially, the values of $\underline{\mathbf{z}}$ are randomly sampled along the dimensions b, d, and j, but remain constant across m, denoted as $\underline{\mathbf{z}}_{b,d,m,j} = \underline{\mathbf{z}}_{b,d,j}\ \forall m$. Subsequently, all elements except the isolated element $\underline{\mathbf{z}}_{j=j^*}$, which retains its original value from $\underline{\mathbf{z}}_j$, are set to zero.
- $\hat{\underline{\boldsymbol{\theta}}}^{(b,d)}$ is defined as $\hat{\underline{\boldsymbol{\theta}}}^{(b,d)} = \{\hat{\boldsymbol{\theta}}_{(b,d,1)}, \hat{\boldsymbol{\theta}}_{(b,d,2)}, \ldots, \hat{\boldsymbol{\theta}}_{(b,d,M)}\}$ and treated as a 4D tensor. It is indexed based on a batch index b, a sample index in the batch d, a sub-sampling index m, and a latent vector dimension index k. The values of $\hat{\underline{\boldsymbol{\theta}}}$ are randomly sampled along the dimensions b, d, m, and k, and then sorted in ascending order over the index m.

Algorithm 1 Selecting $\Omega, \mathbf{z}$, and θ

Input:

- $\Omega_i = \min_{d'} \mathrm{KL}_{d,m,d'}\big(Q(v) \parallel q(v)\big)$.
- $v_i^{max} = \mathrm{argmax}_v\, q(v|\mathbf{z}^{(d,m)}, \theta^{(d,m)})$.
- $\mathbf{z}_i^*$: realized $\mathbf{z}$ values at the current MCS.
- θ_i^*: realized θ values at the current MCS.
- τ: threshold of Ω for selecting $\mathbf{z}_i^*$ and θ_i^*.

Initialization: $\vec{\mathbf{z}}, \vec{\theta} \leftarrow \emptyset$

Procedure:

for each $l \in \{1, 2, \ldots, L\}$ (MCS Iteration) **do**
  $I \leftarrow \{(d,m) | d \in \{1, 2, \ldots, |D|\}, m \in \{1, 2, \ldots, M\}\}$
  for each v in the frequency domain **do**
    $I^v \leftarrow \{i \in I | v_i^{max} = v\}$
    $i^\Omega \leftarrow \mathrm{argmin}_{i \in I^v} \Omega_i$
    if $\Omega_{i=i^\Omega} < \tau$ **then**
      $\vec{\mathbf{z}} \leftarrow \mathrm{CONCAT}(\vec{\mathbf{z}}, \mathbf{z}^*_{i=i^\Omega})$
      $\vec{\theta} \leftarrow \mathrm{CONCAT}(\vec{\theta}, \theta^*_{i=i^\Omega})$
    end if
  end for
end for

Return $\vec{\mathbf{z}}$ and $\vec{\theta}$

In our experiments, τ was set to 1. MCS denotes Monte Carlo sampling.

IV. Fidelity Metrics

A. Operational Details of Filtering Quality Index

The Filtering Quality Index (FQI) quantifies spectral similarity between generated and real signals. FQI is computed over a filtered subset of generated samples whose spectral profiles closely resemble those of real signals, thereby emphasizing outputs that more accurately capture the intended waveform shape–amplitude fusion. To reduce computational overhead, the filtering process is performed in mini-batches.

To evaluate spectral similarity, the KL divergence is computed across frequencies between each synthesized output and all reference signals. Let $\mathbf{Y} = \{\mathbf{y}_1, \ldots, \mathbf{y}_N\}$ denote the full set of original signals, and let $D \subseteq \mathbf{Y}$ represent a reference batch. Let $q(v \mid \mathbf{z}^{(d,m)}, \theta^{(d,m)})$ denote the normalized PSD of the m-th synthesis conditioned on the d-th input, and let $Q(v \mid \mathbf{y}_{d'})$ denote that of the d'-th reference signal, where $\mathbf{y}_{d'} \in D$. The divergence is defined as:

$$\mathrm{KL}_{d,m,d'}\big(Q(v) \parallel q(v)\big) = \sum_{v \in \mathcal{V}} Q(v \mid \mathbf{y}_{d'}) \log \left(\frac{Q(v \mid \mathbf{y}_{d'})}{q(v \mid \mathbf{z}^{(d,m)}, \theta^{(d,m)})} \right). \tag{S62}$$

Here, the indices $d \in \{1, \ldots, |D|\}$, $m \in \{1, \ldots, M\}$, and $d' \in \{1, \ldots, |D|\}$ traverse their respective dimensions to compute all pairwise spectral divergences between the $|D| \times M$ synthesized outputs and the $|D|$ reference signals. For each output (d, m), its closest spectral match among the reference signals is identified by selecting the minimum KL divergence:

$$\Omega_i = \min_{d'} \mathrm{KL}_{d,m,d'}\big(Q(v) \parallel q(v)\big), \quad \text{where } i = (d, m). \tag{S63}$$

Subsequently, Algorithm 1 is applied to extract synthesized samples with high spectral fidelity. Conceptually, the algorithm searches the set of synthesized outputs based on their PSD values and selects $(\mathbf{z}, \theta)$ pairs that yield the lowest spectral divergence from real signals, subject to a predefined threshold τ. This process produces the filtered sets $\vec{\mathbf{z}}$ and $\vec{\theta}$, which represent the latent shape encodings and amplitude modulation parameters associated with high-quality signal synthesis.

Ultimately, we evaluate the overall quality of the filtered-synthesized signals using the following expression:

$$\mathrm{FQI} = \frac{1}{2}\left[\mathrm{KL}\big(P(v|\mathbf{y}) \parallel Q(v|\vec{\mathbf{z}}, \vec{\theta})\big) + \mathrm{KL}\big(Q(v|\vec{\mathbf{z}}, \vec{\theta}) \parallel P(v|\mathbf{y})\big)\right]. \tag{S64}$$

Here, $P(v|\mathbf{y}) = \frac{1}{N}\sum_{n=1}^{N} P(v|\mathbf{y}_n)$ represents the average PSD over the entire dataset $\mathbf{Y}$, while $Q(v|\vec{\mathbf{z}}, \vec{\theta}) = \frac{1}{|\vec{\mathbf{z}}|}\sum_{i=1}^{|\vec{\mathbf{z}}|} Q(v|\vec{\mathbf{z}}_i, \vec{\theta}_i)$ denotes that of the filtered-synthesized outputs.

V. Realization of the VABAM Architecture

A. Training Architecture

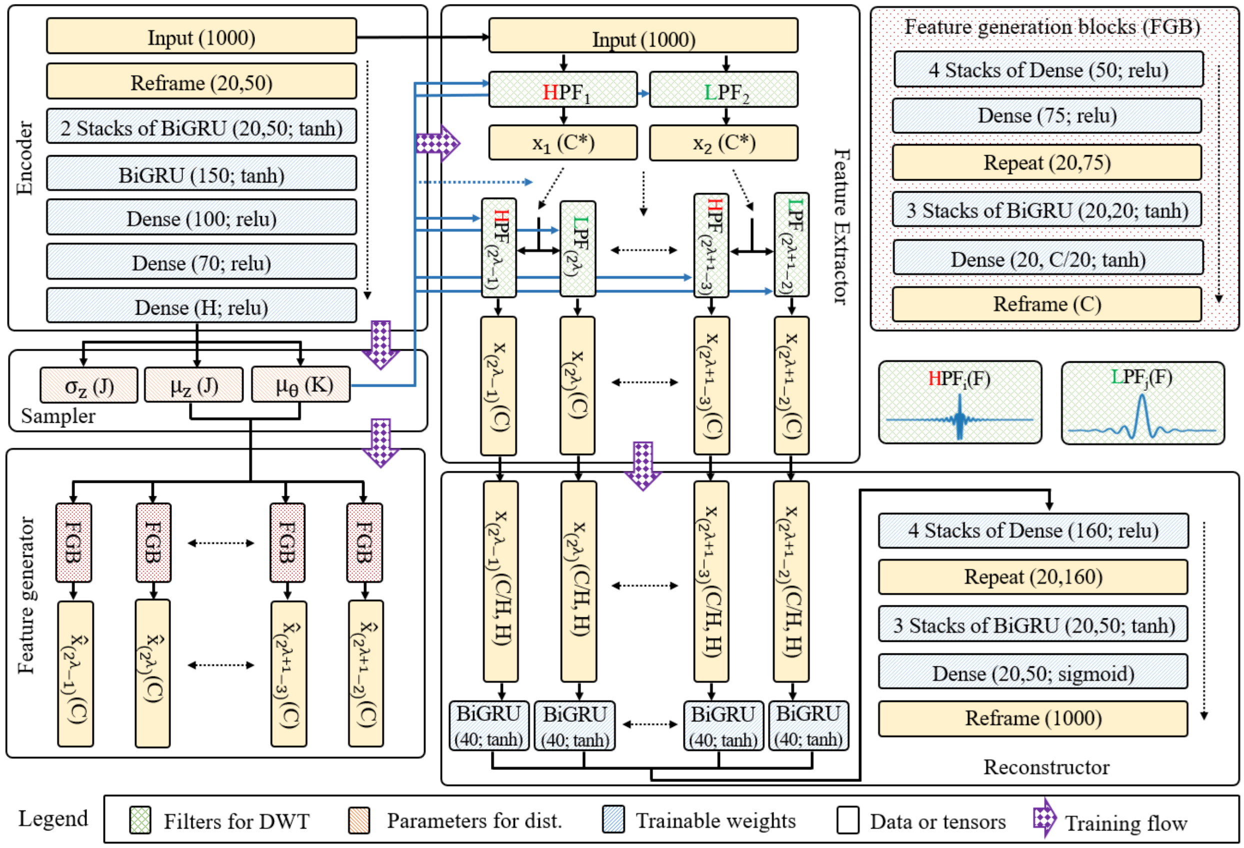

Fig. S1: Training architecture of VABAM for decoupling waveform shape and amplitude. The core design of VABAM lies in the integration of structured latent representations with a cascaded filtering mechanism, enabling effective decoupling of waveform shape and amplitude. While the framework is architecture-agnostic, in this study, it is instantiated using a bidirectional GRU-based encoder–decoder. Raw waveforms are first decomposed into high- and low-frequency components through a sequence of cascaded filters composed of high-pass filter (HPF) and low-pass filter (LPF) operations, implementing a discrete wavelet transform (DWT). This process produces a set of multi-resolution signals, each capturing distinct spectral characteristics across progressively narrower frequency bands: $\mathbf{x} \in \{\mathbf{x}_{2^{\lambda}-1}, \mathbf{x}_{2^{\lambda}}, \ldots, \mathbf{x}_{2^{\lambda+1}-3}, \mathbf{x}_{2^{\lambda+1}-2}\}$. These signals serve as supervision targets for the feature generator and are also used as inputs for waveform reconstruction by the reconstructor. The feature generator comprises feature generation blocks (FGB), whose architecture is detailed in the right panel. Simultaneously, the raw input is processed by the encoder to estimate latent parameters representing waveform shape ($\mu_{\mathbf{z}}$, $\sigma_{\mathbf{z}}$) and amplitude variation ($\mu_{\boldsymbol{\theta}}$). The feature generator then predicts corresponding estimates $\hat{\mathbf{x}}$, conditioned on the learned latent codes.
C denotes the compressed signal length at the terminal nodes, and C^* represents the signal length at intermediate nodes within the cascade. F, H, J, and K denote the filter length, the hop size between consecutive segments, the dimension of $\mathbf{z}$, and the dimension of $\boldsymbol{\theta}$, respectively, where dist. denotes distribution.

B. Synthesis Architecture

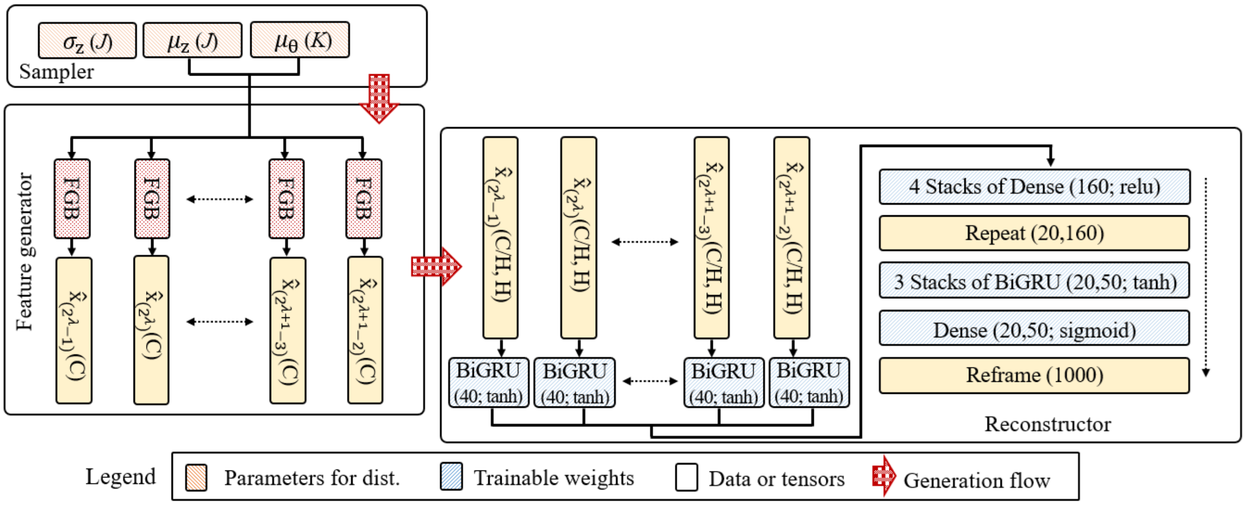

Fig. S2: Synthesis architecture of VABAM for waveform shape–amplitude fusion. Latent representations ($\mu_{\mathbf{z}}$, $\sigma_{\mathbf{z}}$, and μ_{θ}) are used by the feature generation blocks (FGB) to generate feature subsets that capture multi-scale signal characteristics. The generated features are then sequentially decoded through a series of stacked bidirectional GRU (BiGRU) layers followed by dense networks, leading to the synthesis of the output waveform.
C denotes the compressed signal size, and H denotes the hop size between consecutive segments. J and K denote the sizes of dimensions $\mathbf{z}$ and θ, respectively, where dist. denotes distribution.

VI. Comprehensive Benchmark Comparison Results

TABLE S1: Detailed comparison of ABP and ECG synthesis on MIMIC and VitalDB datasets using the FFT. Reported metrics include waveform shape factorization (SF ↑), shape preservation (SP ↑), amplitude modulation controllability (AC ↑), spectral similarity (SS ↑), and reconstruction accuracy (RA ↑), along with three aggregated ISCORE measures—AM, GM, and HM. For VABAM, parentheses in the ISCORE columns indicate scores computed without SF. † and ‡ denote the best and second-best results, respectively.

Dataset – Signal	Metrics					ISCORE		
Model	SF	SP	AC	SS	RA	HM	GM	AM
Mimic – ABP								
C-VAE	0.309	0.080	0.628‡	0.604	0.982	0.250	0.392	0.521
FAC-VAE	0.339	0.076	0.578	0.620	0.987‡	0.245	0.391	0.520
TC-VAE	0.479‡	0.020	0.615	0.596	0.984	0.089	0.322	0.539
VD-VAE	0.347	0.033	0.449	0.608	0.987†	0.132	0.315	0.485
DiffWave	-	0.940‡	0.032	0.689‡	0.961	0.116	0.376	0.655
VDWave	-	0.947†	0.029	0.691†	0.961	0.106	0.368	0.657‡
WaveNet	-	0.217	0.305	0.637	0.967	0.381‡	0.449‡	0.531
VABAM (no SF)	0.999†	0.691	0.892†	0.605	0.978	0.801† (0.763)	0.817† (0.777)	0.833† (0.792)
Mimic – ECG								
C-VAE	0.973‡	0.018	0.714	0.420	0.425	0.079	0.295	0.510
FAC-VAE	0.908	0.008	0.623	0.385	0.460	0.039	0.242	0.477
TC-VAE	0.832	0.143	0.744‡	0.345	0.088	0.210‡	0.306	0.430
VD-VAE	0.481	0.044	0.547	0.429	0.482	0.160	0.299	0.396
DiffWave	-	0.920‡	0.031	0.883‡	0.880‡	0.113	0.387‡	0.678‡
VDWave	-	0.927†	0.029	0.884†	0.882†	0.105	0.380	0.680†
WaveNet	-	0.095	0.200	0.564	0.068	0.125	0.164	0.232
VABAM (no SF)	0.998†	0.428	0.892†	0.403	0.395	0.528† (0.472)	0.571† (0.497)	0.623 (0.530)
VitalDB – ABP								
C-VAE	0.323	0.033	0.695‡	0.698	0.977†	0.135	0.348	0.545
FAC-VAE	0.357	0.046	0.578	0.711	0.976‡	0.174	0.366	0.534
TC-VAE	0.490‡	0.073	0.425	0.720	0.967	0.244	0.403‡	0.535
VD-VAE	0.311	0.034	0.536	0.719	0.975	0.137	0.332	0.515
DiffWave	-	0.929‡	0.031	0.795†	0.964	0.113	0.386	0.680‡
VDWave	-	0.932†	0.029	0.791‡	0.964	0.106	0.379	0.679
WaveNet	-	0.103	0.263	0.691	0.966	0.250‡	0.367	0.506
VABAM (no SF)	0.999†	0.737	0.917†	0.719	0.968	0.851† (0.821)	0.860† (0.828)	0.868† (0.835)
VitalDB – ECG								
C-VAE	0.837‡	0.028	0.695	0.557	0.871	0.123	0.381	0.598
FAC-VAE	0.836	0.020	0.724	0.637	0.861	0.092	0.368	0.616
TC-VAE	0.758	0.111	0.537	0.579	0.829	0.330	0.465‡	0.563
VD-VAE	0.623	0.026	0.730‡	0.612	0.877	0.113	0.364	0.574
DiffWave	-	0.721‡	0.028	0.852†	0.893†	0.103	0.353	0.623
VDWave	-	0.727†	0.028	0.852‡	0.892‡	0.102	0.353	0.625‡
WaveNet	-	0.217	0.295	0.627	0.695	0.363‡	0.409	0.459
VABAM (no SF)	0.981†	0.509	0.836†	0.426	0.806	0.644† (0.593)	0.678† (0.618)	0.712† (0.644)

TABLE S2: Detailed comparison of ABP and ECG synthesis on MIMIC and VitalDB datasets using the DCT. Reported metrics include waveform shape factorization (SF ↑), shape preservation (SP ↑), amplitude modulation controllability (AC ↑), spectral similarity (SS ↑), and reconstruction accuracy (RA ↑), along with three aggregated ISCORE measures—AM, GM, and HM. For VABAM, parentheses in the ISCORE columns indicate scores computed without SF. † and ‡ denote the best and second-best results, respectively.

Dataset – Signal<br>Model	SF	SP	Metrics<br>AC	SS	RA	HM	ISCORE<br>GM	AM
Mimic – ABP								
C-VAE	0.537	0.039	0.609‡	0.622	0.982	0.156	0.378	0.558
FAC-VAE	0.572	0.025	0.558	0.642	0.987‡	0.107	0.346	0.557
TC-VAE	0.643‡	0.007	0.595	0.609	0.984	0.034	0.277	0.567
VD-VAE	0.557	0.015	0.444	0.631	0.987†	0.070	0.298	0.527
DiffWave	-	0.935‡	0.045	0.769†	0.961	0.156	0.420‡	0.677
VDWave	-	0.943†	0.038	0.768‡	0.961	0.136	0.404	0.678‡
WaveNet	-	0.085	0.474	0.744	0.967	0.246‡	0.413	0.567
VABAM<br>(no SF)	1.000†	0.597	0.891†	0.615	0.978	0.776†<br>(0.734)	0.796†<br>(0.752)	0.816†<br>(0.770)
Mimic – ECG								
C-VAE	0.971‡	0.006	0.742	0.199	0.425	0.027	0.203	0.469
FAC-VAE	0.908	0.006	0.644	0.168	0.460	0.029	0.195	0.437
TC-VAE	0.874	0.077	0.745‡	0.172	0.088	0.153	0.237	0.391
VD-VAE	0.638	0.023	0.556	0.216	0.482	0.093	0.243	0.383
DiffWave	-	0.828‡	0.156	0.905‡	0.880‡	0.406†	0.566†	0.692‡
VDWave	-	0.847†	0.146	0.906†	0.882†	0.390	0.561‡	0.695†
WaveNet	-	0.006	0.372	0.514	0.068	0.022	0.095	0.240
VABAM<br>(no SF)	0.999†	0.338	0.869†	0.193	0.395	0.390‡<br>(0.338)	0.468<br>(0.387)	0.559<br>(0.449)
VitalDB – ABP								
C-VAE	0.599	0.016	0.700‡	0.690	0.977†	0.074	0.340	0.597
FAC-VAE	0.618	0.017	0.593	0.702	0.976‡	0.078	0.336	0.581
TC-VAE	0.736‡	0.036	0.435	0.716	0.967	0.148	0.381	0.578
VD-VAE	0.581	0.014	0.534	0.705	0.975	0.066	0.314	0.562
DiffWave	-	0.919‡	0.058	0.841‡	0.964	0.194‡	0.456‡	0.695
VDWave	-	0.927†	0.049	0.842†	0.964	0.169	0.438	0.695‡
WaveNet	-	0.030	0.361	0.803	0.966	0.105	0.304	0.540
VABAM<br>(no SF)	1.000†	0.674	0.912†	0.706	0.968	0.829†<br>(0.795)	0.841†<br>(0.805)	0.852†<br>(0.815)
VitalDB – ECG								
C-VAE	0.858	0.011	0.739‡	0.403	0.871	0.049	0.298	0.576
FAC-VAE	0.865‡	0.010	0.730	0.518	0.861	0.048	0.310	0.597
TC-VAE	0.839	0.049	0.607	0.476	0.829	0.189	0.397	0.560
VD-VAE	0.742	0.015	0.736	0.473	0.877	0.068	0.320	0.569
DiffWave	-	0.557‡	0.125	0.857‡	0.893†	0.332	0.481	0.608
VDWave	-	0.564†	0.126	0.858†	0.892‡	0.333‡	0.483‡	0.610‡
WaveNet	-	0.050	0.456	0.502	0.695	0.155	0.298	0.426
VABAM<br>(no SF)	0.998†	0.423	0.836†	0.247	0.806	0.508†<br>(0.453)	0.588†<br>(0.516)	0.662†<br>(0.578)

TABLE S3: Detailed comparison of ABP and ECG synthesis on MIMIC and VitalDB datasets using the WSE. Reported metrics include waveform shape factorization (SF ↑), shape preservation (SP ↑), amplitude modulation controllability (AC ↑), spectral similarity (SS ↑), and reconstruction accuracy (RA ↑), along with three aggregated ISCORE measures—AM, GM, and HM. For VABAM, parentheses in the ISCORE columns indicate scores computed without SF. † and ‡ denote the best and second-best results, respectively.

Dataset – Signal	Metrics					ISCORE		
Model	SF	SP	AC	SS	RA	HM	GM	AM
Mimic – ABP								
C-VAE	0.196	0.481	0.636	0.682	0.982	0.445‡	0.526	0.595
FAC-VAE	0.189	0.461	0.696‡	0.705†	0.987‡	0.442	0.531‡	0.608
TC-VAE	0.365‡	0.191	0.665	0.690	0.984	0.419	0.501	0.579
VD-VAE	0.213	0.361	0.529	0.700	0.987†	0.424	0.489	0.558
DiffWave	-	0.905‡	0.106	0.575	0.961	0.301	0.480	0.637
VDWave	-	0.938†	0.090	0.588	0.961	0.269	0.468	0.644‡
WaveNet	-	0.179	0.340	0.454	0.967	0.340	0.404	0.485
VABAM (no SF)	0.927†	0.863	0.905†	0.703‡	0.978	0.864† (0.850)	0.870† (0.856)	0.875† (0.862)
Mimic – ECG								
C-VAE	0.880‡	0.128	0.765	0.497	0.425	0.342	0.449	0.539
FAC-VAE	0.853	0.077	0.724	0.501	0.460	0.253	0.405	0.523
TC-VAE	0.525	0.486	0.823‡	0.075	0.088	0.167	0.268	0.399
VD-VAE	0.443	0.322	0.639	0.638	0.482	0.473‡	0.489‡	0.505
DiffWave	-	0.940‡	0.047	0.851‡	0.880‡	0.161	0.426	0.679‡
VDWave	-	0.954†	0.033	0.856†	0.882†	0.120	0.393	0.681†
WaveNet	-	0.111	0.233	0.536	0.068	0.134	0.175	0.237
VABAM (no SF)	0.942†	0.599	0.907†	0.494	0.395	0.596† (0.546)	0.631† (0.571)	0.667 (0.599)
VitalDB – ABP								
C-VAE	0.181	0.330	0.710‡	0.757	0.977†	0.406	0.500	0.591
FAC-VAE	0.190	0.360	0.624	0.795‡	0.976‡	0.419	0.506‡	0.589
TC-VAE	0.231‡	0.350	0.457	0.783	0.967	0.428‡	0.489	0.558
VD-VAE	0.168	0.352	0.596	0.779	0.975	0.391	0.485	0.574
DiffWave	-	0.857	0.081	0.572	0.964	0.245	0.442	0.618
VDWave	-	0.874‡	0.064	0.572	0.964	0.203	0.418	0.619‡
WaveNet	-	0.093	0.285	0.515	0.966	0.232	0.339	0.465
VABAM (no SF)	0.845†	0.887†	0.931†	0.846†	0.968	0.893† (0.906)	0.894† (0.907)	0.895† (0.908)
VitalDB – ECG								
C-VAE	0.665‡	0.257	0.731	0.702	0.871	0.536	0.598	0.645‡
FAC-VAE	0.602	0.170	0.756‡	0.764	0.861	0.441	0.551	0.631
TC-VAE	0.533	0.439	0.558	0.716	0.829	0.585‡	0.599‡	0.615
VD-VAE	0.470	0.118	0.731	0.787	0.877	0.347	0.489	0.597
DiffWave	-	0.745‡	0.036	0.846‡	0.893†	0.128	0.378	0.630
VDWave	-	0.781†	0.036	0.854†	0.892‡	0.126	0.381	0.641
WaveNet	-	0.382	0.299	0.623	0.695	0.444	0.471	0.500
VABAM (no SF)	0.740†	0.716	0.845†	0.705	0.806	0.759† (0.764)	0.761† (0.766)	0.762† (0.768)

VII. Comprehensive Ablation Study Results

TABLE S4: Ablation study of ABP and ECG synthesis on MIMIC and VitalDB datasets using the FFT. This table compares three model variants: only-$\boldsymbol{\theta}$-prior (frequency-domain prior only), only-$\mathbf{z}$-prior (latent-space prior only), and complete (full model with both priors). Reported metrics include waveform shape factorization (SF ↑), shape preservation (SP ↑), amplitude modulation controllability (AC ↑), spectral similarity (SS ↑), and reconstruction accuracy (RA ↑), along with three aggregated ISCORE measures—AM, GM, and HM. † and ‡ denote the best and second-best results, respectively.

Dataset – Signal	Metrics					ISCORE		
Model	SF	SP	AC	SS	RA	HM	GM	AM
Mimic – ABP								
only-$\boldsymbol{\theta}$-prior	0.999‡	0.071	0.716	0.628†	0.979‡	0.261‡	0.499‡	0.679
only-$\mathbf{z}$-prior	0.999	1.000†	0.911†	0.004	0.971	0.021	0.328	0.777‡
complete	1.000†	0.712‡	0.905‡	0.565‡	0.981†	0.794†	0.814†	0.833†
Mimic – ECG								
only-$\boldsymbol{\theta}$-prior	0.998‡	0.320	0.860	0.412†	0.368‡	0.479‡	0.530‡	0.592
only-$\mathbf{z}$-prior	0.999†	1.000†	0.905†	0.214	0.179	0.374	0.510	0.659†
complete	0.998	0.475‡	0.885‡	0.380‡	0.393†	0.531†	0.575†	0.626‡
VitalDB – ABP								
only-$\boldsymbol{\theta}$-prior	1.000‡	0.366	0.880	0.735†	0.966†	0.688	0.744	0.789
only-$\mathbf{z}$-prior	0.964	1.000†	0.906‡	0.364	0.956	0.721‡	0.788‡	0.838‡
complete	1.000†	0.801‡	0.930†	0.710‡	0.966‡	0.867†	0.874†	0.881†
VitalDB – ECG								
only-$\boldsymbol{\theta}$-prior	0.991†	0.368	0.871‡	0.450†	0.819†	0.601‡	0.651	0.700
only-$\mathbf{z}$-prior	0.988	1.000†	0.909†	0.254	0.747	0.597	0.702‡	0.780†
complete	0.991‡	0.572‡	0.865	0.431‡	0.810‡	0.670†	0.703†	0.734‡

TABLE S5: Ablation study of ABP and ECG synthesis on MIMIC and VitalDB datasets using the DCT. This table compares three model variants: only-$\boldsymbol{\theta}$-prior (frequency-domain prior only), only-$\mathbf{z}$-prior (latent-space prior only), and complete (full model with both priors). Reported metrics include waveform shape factorization (SF ↑), shape preservation (SP ↑), amplitude modulation controllability (AC ↑), spectral similarity (SS ↑), and reconstruction accuracy (RA ↑), along with three aggregated ISCORE measures—AM, GM, and HM. † and ‡ denote the best and second-best results, respectively.

Dataset – Signal	Metrics					ISCORE		
Model	SF	SP	AC	SS	RA	HM	GM	AM
Mimic – ABP								
only-$\boldsymbol{\theta}$-prior	1.000	0.051	0.703‡	0.627†	0.979‡	0.201‡	0.465‡	0.672
only-$\mathbf{z}$-prior	1.000†	0.999†	0.666	0.010	0.971	0.048	0.365	0.729‡
complete	1.000‡	0.647‡	0.900†	0.598‡	0.981†	0.788†	0.807†	0.825†
Mimic – ECG								
only-$\boldsymbol{\theta}$-prior	1.000‡	0.252	0.863†	0.199†	0.368‡	0.361‡	0.437	0.536
only-$\mathbf{z}$-prior	1.000†	1.000†	0.668	0.186	0.179	0.346	0.467‡	0.607†
complete	0.999	0.388‡	0.854‡	0.190‡	0.393†	0.398†	0.477†	0.565‡
VitalDB – ABP								
only-$\boldsymbol{\theta}$-prior	1.000‡	0.293	0.880‡	0.720†	0.966†	0.627	0.709	0.772
only-$\mathbf{z}$-prior	0.992	1.000†	0.653	0.331	0.956	0.657‡	0.728‡	0.786‡
complete	1.000†	0.757‡	0.924†	0.691‡	0.966‡	0.850†	0.859†	0.868†
VitalDB – ECG								
only-$\boldsymbol{\theta}$-prior	0.998	0.308	0.878†	0.387†	0.819†	0.544†	0.612†	0.678
only-$\mathbf{z}$-prior	1.000†	1.000†	0.670	0.133	0.747	0.404	0.581	0.710†
complete	0.999‡	0.477‡	0.863‡	0.252‡	0.810‡	0.529‡	0.610‡	0.680‡

TABLE S6: Ablation study of ABP and ECG synthesis on MIMIC and VitalDB datasets using the WSE. This table compares three model variants: only-$\boldsymbol{\theta}$-prior (frequency-domain prior only), only-$\mathbf{z}$-prior (latent-space prior only), and complete (full model with both priors). Reported metrics include waveform shape factorization (SF ↑), shape preservation (SP ↑), amplitude modulation controllability (AC ↑), spectral similarity (SS ↑), and reconstruction accuracy (RA ↑), along with three aggregated ISCORE measures—AM, GM, and HM. † and ‡ denote the best and second-best results, respectively.

Dataset – Signal	Metrics					ISCORE		
Model	SF	SP	AC	SS	RA	HM	GM	AM
Mimic – ABP								
only-$\boldsymbol{\theta}$-prior	0.965†	0.201	0.739	0.746†	0.979‡	0.514‡	0.637‡	0.726
only-$\mathbf{z}$-prior	0.942	1.000†	0.924†	0.045	0.971	0.188	0.519	0.776‡
complete	0.948‡	0.894‡	0.920‡	0.666‡	0.981†	0.865†	0.874†	0.882†
Mimic – ECG								
only-$\boldsymbol{\theta}$-prior	0.904‡	0.492	0.875	0.481†	0.368‡	0.551‡	0.586‡	0.624‡
only-$\mathbf{z}$-prior	0.892	1.000†	0.916†	0.093	0.179	0.255	0.423	0.616
complete	0.954†	0.661‡	0.906‡	0.441‡	0.393†	0.590†	0.630†	0.671†
VitalDB – ABP								
only-$\boldsymbol{\theta}$-prior	0.963†	0.617	0.891	0.825‡	0.966†	0.829	0.841	0.852
only-$\mathbf{z}$-prior	0.814	1.000†	0.915‡	0.762	0.956	0.880‡	0.885‡	0.889‡
complete	0.920‡	0.908‡	0.950†	0.836†	0.966‡	0.914†	0.915†	0.916†
VitalDB – ECG								
only-$\boldsymbol{\theta}$-prior	0.855†	0.638	0.870‡	0.621‡	0.819†	0.744‡	0.753‡	0.761
only-$\mathbf{z}$-prior	0.783	1.000†	0.909†	0.446	0.747	0.719	0.750	0.777‡
complete	0.836‡	0.740‡	0.870	0.673†	0.810‡	0.779†	0.783†	0.786†

VIII. Comprehensive Sensitivity Analysis Results

TABLE S7: Hyperparameter sensitivity analysis for the FFT. Reported metrics include waveform shape factorization (SF ↑), shape preservation (SP ↑), amplitude modulation controllability (AC ↑), spectral similarity (SS ↑), and reconstruction accuracy (RA ↑), along with three aggregated ISCORE measures—AM, GM, and HM. Results are presented as mean (s.d.) across independent experiments for each parameter setting. † indicates the best-performing setting within each parameter group.

				Metrics					ISCORE		
Dataset	Signal	Parameter	Setting	SF	SP	AC	SS	RA	HM	GM	AM
VitalDB	ABP	C	500	1.000† (0.000)	0.660 (0.082)	0.852 (0.062)	0.698 (0.008)	0.946 (0.002)	0.808 (0.034)	0.820 (0.031)	0.831 (0.027)
		C	800	1.000 (0.000)	0.748† (0.058)	0.853† (0.096)	0.723† (0.013)	0.969† (0.003)	0.842† (0.033)	0.850† (0.031)	0.858† (0.029)
		J	30	1.000 (0.000)	0.710 (0.077)	0.866† (0.049)	0.714 (0.021)	0.958 (0.014)	0.831† (0.033)	0.840† (0.030)	0.850† (0.026)
		J	50	1.000† (0.000)	0.736† (0.092)	0.825 (0.149)	0.715† (0.007)	0.969† (0.004)	0.830 (0.052)	0.839 (0.049)	0.849 (0.046)
		ζ	1	1.000 (0.000)	0.752† (0.043)	0.914† (0.017)	0.707 (0.013)	0.961 (0.011)	0.850† (0.017)	0.858† (0.016)	0.867† (0.015)
		ζ	2	1.000† (0.000)	0.685 (0.091)	0.791 (0.064)	0.722† (0.019)	0.963† (0.015)	0.811 (0.039)	0.822 (0.035)	0.832 (0.031)
VitalDB	ECG	C	500	0.990 (0.007)	0.485† (0.140)	0.787 (0.000)	0.453 (0.058)	0.799 (0.010)	0.633† (0.026)	0.669 (0.021)	0.703 (0.016)
		C	800	0.991† (0.007)	0.460 (0.096)	0.817† (0.041)	0.456† (0.035)	0.808† (0.004)	0.633 (0.029)	0.670† (0.025)	0.707† (0.021)
		J	30	0.989 (0.007)	0.463 (0.101)	0.797 (0.027)	0.458† (0.045)	0.802 (0.007)	0.629 (0.021)	0.666 (0.017)	0.702 (0.013)
		J	50	0.994† (0.004)	0.480† (0.131)	0.828† (0.053)	0.451 (0.028)	0.811† (0.002)	0.639† (0.043)	0.677† (0.037)	0.713† (0.030)
		ζ	1	0.989 (0.007)	0.555† (0.040)	0.830† (0.039)	0.423 (0.010)	0.802 (0.010)	0.655† (0.013)	0.688† (0.013)	0.720† (0.012)
		ζ	2	0.993† (0.007)	0.382 (0.007)	0.785 (0.007)	0.488† (0.015)	0.807† (0.005)	0.611 (0.003)	0.651 (0.002)	0.691 (0.001)
Mimic	ABP	C	500	1.000† (0.000)	0.594 (0.132)	0.851† (0.087)	0.639† (0.017)	0.960 (0.001)	0.769 (0.054)	0.790 (0.047)	0.809 (0.040)
		C	800	1.000 (0.000)	0.654† (0.065)	0.815 (0.099)	0.594 (0.033)	0.982† (0.002)	0.772† (0.030)	0.790† (0.030)	0.809† (0.028)
		J	30	1.000 (0.000)	0.632 (0.090)	0.837† (0.077)	0.613† (0.034)	0.970 (0.012)	0.773† (0.037)	0.792† (0.033)	0.811† (0.029)
		J	50	1.000† (0.000)	0.639† (0.103)	0.806 (0.140)	0.600 (0.050)	0.982† (0.001)	0.766 (0.039)	0.786 (0.040)	0.805 (0.038)
		ζ	1	1.000† (0.000)	0.697† (0.013)	0.903† (0.010)	0.599 (0.031)	0.973 (0.012)	0.801† (0.007)	0.818† (0.004)	0.834† (0.002)
		ζ	2	1.000 (0.000)	0.572 (0.074)	0.751 (0.042)	0.619† (0.043)	0.976† (0.013)	0.741 (0.011)	0.762 (0.009)	0.783 (0.007)
Mimic	ECG	C	500	0.981 (0.026)	0.688† (0.300)	0.836 (0.056)	0.269 (0.166)	0.361 (0.050)	0.453 (0.074)	0.542 (0.018)	0.627† (0.020)
		C	800	0.998† (0.001)	0.420 (0.043)	0.845† (0.059)	0.378† (0.020)	0.387† (0.010)	0.510† (0.023)	0.553† (0.023)	0.606 (0.022)
		J	30	0.990 (0.018)	0.552† (0.234)	0.832 (0.062)	0.330 (0.119)	0.373 (0.033)	0.482 (0.056)	0.547 (0.019)	0.615† (0.024)
		J	50	0.998† (0.001)	0.423 (0.073)	0.862† (0.032)	0.367† (0.018)	0.390† (0.005)	0.508† (0.032)	0.553† (0.030)	0.608 (0.026)
		ζ	1	0.998† (0.001)	0.460 (0.027)	0.884† (0.008)	0.390† (0.012)	0.371 (0.039)	0.521† (0.014)	0.567† (0.011)	0.621† (0.007)
		ζ	2	0.987 (0.021)	0.558† (0.296)	0.800 (0.038)	0.294 (0.124)	0.386† (0.012)	0.460 (0.052)	0.532 (0.002)	0.605 (0.032)

TABLE S8: Hyperparameter sensitivity analysis for the DCT. Reported metrics include waveform shape factorization (SF ↑), shape preservation (SP ↑), amplitude modulation controllability (AC ↑), spectral similarity (SS ↑), and reconstruction accuracy (RA ↑), along with three aggregated ISCORE measures—AM, GM, and HM. Results are presented as mean (s.d.) across independent experiments for each parameter setting. † indicates the best-performing setting within each parameter group.

				Metrics					ISCORE		
Dataset	Signal	Parameter	Setting	SF	SP	AC	SS	RA	HM	GM	AM
VitalDB	ABP	C	500	1.000† (0.000)	0.592 (0.140)	0.848† (0.061)	0.686 (0.016)	0.946 (0.002)	0.779 (0.056)	0.797 (0.046)	0.815 (0.038)
		C	800	1.000 (0.000)	0.671† (0.102)	0.845 (0.098)	0.702† (0.014)	0.969† (0.003)	0.813† (0.051)	0.825† (0.045)	0.837† (0.039)
		J	30	1.000 (0.000)	0.646† (0.104)	0.860† (0.050)	0.700† (0.019)	0.958 (0.014)	0.805† (0.044)	0.819† (0.037)	0.833† (0.030)
		J	50	1.000† (0.000)	0.642 (0.162)	0.818 (0.150)	0.691 (0.000)	0.969† (0.004)	0.794 (0.079)	0.809 (0.070)	0.824 (0.062)
		ζ	1	1.000† (0.000)	0.707† (0.044)	0.909† (0.017)	0.691 (0.016)	0.961 (0.011)	0.833† (0.016)	0.843† (0.014)	0.854† (0.013)
		ζ	2	1.000 (0.000)	0.582 (0.125)	0.783 (0.063)	0.703† (0.016)	0.963† (0.015)	0.770 (0.055)	0.788 (0.046)	0.806 (0.038)
VitalDB	ECG	C	500	0.998 (0.001)	0.405† (0.151)	0.802 (0.003)	0.330† (0.110)	0.799 (0.010)	0.534† (0.005)	0.604† (0.004)	0.667 (0.007)
		C	800	0.999† (0.001)	0.388 (0.076)	0.826† (0.029)	0.320 (0.103)	0.808† (0.004)	0.531 (0.025)	0.602 (0.018)	0.668† (0.013)
		J	30	0.998 (0.001)	0.387 (0.101)	0.811 (0.017)	0.344† (0.111)	0.802 (0.007)	0.536† (0.024)	0.605† (0.015)	0.668† (0.007)
		J	50	0.999† (0.001)	0.409† (0.097)	0.833† (0.043)	0.282 (0.042)	0.811† (0.002)	0.524 (0.007)	0.598 (0.017)	0.667 (0.019)
		ζ	1	0.999 (0.001)	0.471† (0.045)	0.835† (0.030)	0.251 (0.003)	0.802 (0.010)	0.522 (0.013)	0.601 (0.011)	0.671† (0.009)
		ζ	2	0.999† (0.001)	0.317 (0.021)	0.802 (0.001)	0.396† (0.078)	0.807† (0.005)	0.541† (0.023)	0.603† (0.019)	0.664 (0.012)
Mimic	ABP	C	500	1.000† (0.000)	0.545 (0.135)	0.843† (0.087)	0.661† (0.026)	0.960 (0.001)	0.755† (0.060)	0.779† (0.049)	0.802† (0.039)
		C	800	1.000 (0.000)	0.577† (0.070)	0.807 (0.104)	0.614 (0.040)	0.982† (0.002)	0.752 (0.036)	0.774 (0.033)	0.796 (0.029)
		J	30	1.000 (0.000)	0.568† (0.083)	0.827† (0.084)	0.628 (0.044)	0.970 (0.012)	0.754† (0.039)	0.777† (0.033)	0.799† (0.028)
		J	50	1.000† (0.000)	0.563 (0.118)	0.801 (0.140)	0.633† (0.049)	0.982† (0.001)	0.750 (0.053)	0.773 (0.047)	0.796 (0.041)
		ζ	1	1.000† (0.000)	0.628† (0.027)	0.899† (0.007)	0.618 (0.022)	0.973 (0.012)	0.787† (0.011)	0.806† (0.009)	0.824† (0.007)
		ζ	2	1.000 (0.000)	0.505 (0.071)	0.739 (0.040)	0.640† (0.057)	0.976† (0.013)	0.719 (0.011)	0.746 (0.007)	0.772 (0.005)
Mimic	ECG	C	500	1.000† (0.000)	0.627† (0.341)	0.816 (0.077)	0.158 (0.060)	0.361 (0.050)	0.365 (0.035)	0.481† (0.022)	0.592† (0.051)
		C	800	1.000 (0.000)	0.332 (0.049)	0.837† (0.036)	0.176† (0.021)	0.387† (0.010)	0.370† (0.032)	0.451 (0.027)	0.546 (0.018)
		J	30	1.000† (0.000)	0.482† (0.259)	0.822 (0.056)	0.171† (0.039)	0.373 (0.033)	0.372† (0.024)	0.468† (0.022)	0.569† (0.041)
		J	50	1.000 (0.000)	0.328 (0.084)	0.846† (0.011)	0.168 (0.031)	0.390† (0.005)	0.362 (0.051)	0.447 (0.042)	0.546 (0.026)
		ζ	1	1.000 (0.000)	0.371 (0.028)	0.865† (0.009)	0.194† (0.005)	0.371 (0.039)	0.393† (0.005)	0.470† (0.006)	0.560 (0.004)
		ζ	2	1.000† (0.000)	0.491† (0.329)	0.796 (0.039)	0.145 (0.030)	0.386† (0.012)	0.344 (0.020)	0.453 (0.040)	0.563† (0.056)

TABLE S9: Hyperparameter sensitivity analysis for the WSE. Reported metrics include waveform shape factorization (SF ↑), shape preservation (SP ↑), amplitude modulation controllability (AC ↑), spectral similarity (SS ↑), and reconstruction accuracy (RA ↑), along with three aggregated ISCORE measures—AM, GM, and HM. Results are presented as mean (s.d.) across independent experiments for each parameter setting. † indicates the best-performing setting within each parameter group.

				Metrics					ISCORE		
Dataset	Signal	Parameter	Setting	SF	SP	AC	SS	RA	HM	GM	AM
VitalDB	ABP	C	500	0.906† (0.005)	0.856 (0.036)	0.857 (0.075)	0.781 (0.007)	0.946 (0.002)	0.865 (0.008)	0.867 (0.008)	0.869 (0.008)
		C	800	0.905 (0.041)	0.877† (0.030)	0.891† (0.067)	0.836† (0.007)	0.969† (0.003)	0.893† (0.019)	0.894† (0.019)	0.896† (0.018)
		J	30	0.898 (0.038)	0.869 (0.026)	0.882† (0.056)	0.810 (0.035)	0.958 (0.014)	0.880 (0.018)	0.882 (0.018)	0.883 (0.017)
		J	50	0.920† (0.000)	0.873† (0.050)	0.874 (0.107)	0.832† (0.005)	0.969† (0.004)	0.890† (0.033)	0.892† (0.032)	0.894† (0.031)
		ζ	1	0.891 (0.041)	0.875† (0.040)	0.931† (0.020)	0.819† (0.038)	0.961 (0.011)	0.892† (0.022)	0.894† (0.021)	0.895† (0.021)
		ζ	2	0.919† (0.017)	0.865 (0.024)	0.828 (0.047)	0.816 (0.027)	0.963† (0.015)	0.874 (0.020)	0.876 (0.020)	0.878 (0.019)
VitalDB	ECG	C	500	0.847† (0.061)	0.658 (0.065)	0.801 (0.000)	0.622 (0.001)	0.799 (0.010)	0.734 (0.024)	0.740 (0.023)	0.745 (0.023)
		C	800	0.815 (0.050)	0.670† (0.068)	0.823† (0.041)	0.678† (0.027)	0.808† (0.004)	0.750† (0.024)	0.755† (0.022)	0.759† (0.021)
		J	30	0.819 (0.063)	0.659 (0.059)	0.810 (0.024)	0.648 (0.039)	0.802 (0.007)	0.737 (0.020)	0.743 (0.019)	0.747 (0.017)
		J	50	0.840† (0.005)	0.680† (0.085)	0.828† (0.060)	0.681† (0.012)	0.811† (0.002)	0.760† (0.028)	0.764† (0.026)	0.768† (0.025)
		ζ	1	0.822 (0.076)	0.720† (0.018)	0.839† (0.035)	0.667† (0.042)	0.802 (0.010)	0.763† (0.015)	0.766† (0.014)	0.770† (0.014)
		ζ	2	0.829† (0.021)	0.612 (0.007)	0.793 (0.008)	0.652 (0.034)	0.807† (0.005)	0.727 (0.012)	0.733 (0.011)	0.738 (0.011)
Mimic	ABP	C	500	0.925 (0.025)	0.758 (0.014)	0.862† (0.098)	0.711† (0.007)	0.960 (0.001)	0.831 (0.024)	0.837 (0.025)	0.843 (0.026)
		C	800	0.945† (0.012)	0.826† (0.062)	0.846 (0.084)	0.692 (0.018)	0.982† (0.002)	0.844† (0.024)	0.851† (0.024)	0.858† (0.024)
		J	30	0.931 (0.018)	0.787 (0.052)	0.862† (0.067)	0.705† (0.009)	0.970 (0.012)	0.839 (0.022)	0.845 (0.022)	0.851 (0.022)
		J	50	0.952† (0.006)	0.836† (0.082)	0.830 (0.128)	0.686 (0.029)	0.982† (0.001)	0.841† (0.034)	0.849† (0.035)	0.857† (0.035)
		ζ	1	0.939† (0.011)	0.842† (0.066)	0.919† (0.013)	0.692 (0.023)	0.973 (0.012)	0.859† (0.009)	0.866† (0.010)	0.873† (0.010)
		ζ	2	0.937 (0.026)	0.765 (0.015)	0.784 (0.041)	0.706† (0.011)	0.976† (0.013)	0.820 (0.008)	0.827 (0.008)	0.834 (0.009)
Mimic	ECG	C	500	0.902 (0.063)	0.809† (0.182)	0.858 (0.047)	0.268 (0.147)	0.361 (0.050)	0.473 (0.083)	0.560 (0.038)	0.640 (0.005)
		C	800	0.942† (0.010)	0.611 (0.035)	0.867† (0.056)	0.492† (0.063)	0.387† (0.010)	0.589† (0.019)	0.624† (0.019)	0.660† (0.020)
		J	30	0.919 (0.041)	0.705† (0.160)	0.853 (0.056)	0.371 (0.147)	0.373 (0.033)	0.526 (0.079)	0.586 (0.040)	0.644 (0.017)
		J	50	0.948† (0.007)	0.621 (0.056)	0.885† (0.029)	0.511† (0.098)	0.390† (0.005)	0.598† (0.011)	0.634† (0.006)	0.671† (0.000)
		ζ	1	0.947† (0.006)	0.647 (0.042)	0.901† (0.009)	0.436† (0.062)	0.371 (0.039)	0.573† (0.036)	0.616† (0.025)	0.660† (0.015)
		ζ	2	0.910 (0.046)	0.707† (0.200)	0.826 (0.038)	0.399 (0.213)	0.386† (0.012)	0.528 (0.100)	0.589 (0.053)	0.645 (0.022)

Supplementary References

[1] L. Pinheiro Cinelli, M. Araújo Marins, E. A. Barros da Silva, and S. Lima Netto, "Variational autoencoder," in *Variational Methods for Machine Learning with Applications to Deep Networks*, pp. 111–149, Springer, 2021.

[2] C. P. Burgess, I. Higgins, A. Pal, L. Matthey, N. Watters, G. Desjardins, and A. Lerchner, "Understanding disentangling in $beta$-vae," *arXiv preprint arXiv:1804.03599*, 2018.

[3] C. J. Maddison, A. Mnih, and Y. W. Teh, "The concrete distribution: A continuous relaxation of discrete random variables," *arXiv preprint arXiv:1611.00712*, 2016.

[4] E. Jang, S. Gu, and B. Poole, "Categorical reparameterization with gumbel-softmax," *arXiv preprint arXiv:1611.01144*, 2016.

[5] P. Stoica, R. L. Moses, *et al.*, *Spectral analysis of signals*, vol. 452. Upper Saddle River, NJ: Pearson Prentice Hall, 2005.

[6] K. R. Rao and P. Yip, *Discrete Cosine Transform: Algorithms, Advantages, Applications*. San Diego, CA: Academic Press, 1990.

[7] P. Welch, "The use of fast fourier transform for the estimation of power spectra: A method based on time averaging over short, modified periodograms," *IEEE Transactions on Audio and Electroacoustics*, vol. 15, no. 2, pp. 70–73, 1967.

[8] R. T. Chen, X. Li, R. B. Grosse, and D. K. Duvenaud, "Isolating sources of disentanglement in variational autoencoders," *Advances in neural information processing systems*, vol. 31, 2018.
