# Supplementary material for: Shape and amplitude decoupling in pulsatile physiological signal synthesis and its evaluation
Source: Nat Commun. 2026 Apr 29;17:5876. doi: 10.1038/s41467-026-72299-7 (PMC13333840; doi:10.1038/s41467-026-72299-7)
Supplement: Supplementary file 2 — Transparent Peer Review file [file 41467_2026_72299_MOESM2_ESM.pdf]

nature portfolio

Peer Review File

Shape and Amplitude Decoupling in Pulsatile Physiological Signal Synthesis and Its Evaluation

Corresponding Author: Professor Junetae Kim

This file contains all reviewer reports in order by version, followed by all author rebuttals in order by version.

Version 0:

Reviewer comments:

Reviewer #1

(Remarks to the Author)
I have read with real interest the MS entitled: Morphology–Amplitude Decoupling for Pulsatile Physiological Signal Synthesis: A Probabilistic Model and Evaluation Metrics

It is actually quite frequent, that the morphology of physiological signals may change independently to its amplitude.
The physical process of signal transduction, which constitutes the core part of each biosensor has many cofactors, that typically affect the amplitude of the observed signals. Some changes of amplitude are related to the underlying physical process, and represent the true features of the source of signal, whereas the other are related to the measurement process, sometimes so closely, that it is even hard to refer to them as artifacts or noise - it is just another source of information (unsolicited).
For example loss of amplitude of blood pressure may be related with acute haemorrhage, orthostatic intolerance, external acceleration, but also to external pressure applied to sensor or to measured object, or sudden change of geometry. In case of photoplethysmography it is sufficient to tilt the diode. In case of the ECG there are even more possibilities, as electrode contact, skin humidity (e.g. bridging ECG electrodes using conducting gel), galvanic skin response, electrode tension, electrode motion or water immersion of the extremity may affect the ECG, having nothing to do with the actual state of the source. Therefore it is important to distinguish the changes related to the source (substantial) from those that are caused by parameters of the measurement process itself (non substantial).
The assumption, that one can disentangle the changes of morphology and the changes of amplitude is true only in the first order of approximation. For instance, increase of systolic blood pressure increases the pressure gradient between the arterial and the venous component, which affects the AV outflow. This, in turn, affects the time decrement of the downward slope od the ABP curve, There are many more effects of this type, such as e.g. emergence of the second dichrotic notch and so on. Note also, that in the specific case of ABP, its amplitude actually rises along the vascular tree - this fact was used to build the models, that allow reconstruction of central pressure (e.g. https://patents.google.com/patent/WO2023152546A1/en, https://www.sciencedirect.com/science/article/abs/pii/S1746809422000350)

Methods that build a digital representation of signals emerge in this context as important research tools, that allow to distinguish between substantial and non-substantial changes. Error of reconstruction may be used as a valuable research tool, which quantifies the amount of our ability to reconstruct the process based on the a priori knowledge (c.f. https://bamsjournal.com/article/551381/en).
Parameters of the latent space constitute the language for interpretation of the data, which can be used to construct clinical indices.

It is rare to find a paper that may be accepted "as is", a few minor comments below.
1. p2 line 43 - the ECG does not convey the information on hemodynamic state, rather the information on the electric activity of the cardiac effector
2. Note, that if you use PSD calculation as one of steps of the process, you expose yourself to all kinds of errors related to PSD estimation. The PSD estimation method is not disclosed, or I was unable to find it. Note, that, if the FFT was used, it works in unfavourable conditions, as it has to represent the delta-spike signal, which is locally nonstationary. Note, that you did not mention the phase part of the spectrum. There exists methods that are alternative to the windowed FFT, e.g. Matching Pursuit - c.f. https://sparse-plex.readthedocs.io/en/latest/book/pursuit/mp/algorithm.html or evolutionary periodogram (Welsh). A comment on this would be favourable. Note, that building Fourier representation is also an

optimization problem.
3. p.5 line 122 When you mention segmentation, you don't mention the method for segment definition. Are they based on peak detection and segmented like in the ECG analysis? A comment would be helpful.
4. A choice of theta acute is questionable, as the comma is located on top of the greek letter which is poorly readable. This problem first appears in p. 6 line 186 and 189, but it is also related to different symbols - e.g. Z and z in p. 15 line 432 - note that Z with such a placement of an accent is a regular letter in some languages. If the accent was shifted past the symbol, the readability of text would be much improved.
5. Although you draw conclusions concerning the potential of VABAM in biomedical application, there is no example of application, that could sparkle the imagination - note e.g. the approach taken here:
https://www.sciencedirect.com/science/article/pii/S0022073622002138?via=ihub where an average ECG is displayed as a reference - the commercial tool that utilizes these results may also be of help to you concerning future commercialization path.
https://cineecg.com/

(Remarks on code availability)
I did not run the code, although I can see, that the instructions are very clear and the quality of the digital part of submission is outstanding, as compared to typical open source projects.

Reviewer #2

(Remarks to the Author)
Summary:
The manuscript introduces a novel method (called VABAM) for the generation of synthetic physiological signals that relies on cascaded filtering and that is meant to structurally separate morphology and amplitude in pulsatile physiological signals.

Strengths:
• The cascaded filtering approach and variational design are innovative and carefully described. The mathematical formulation is rigorous, and the architecture appears well thought out.
• The method is tested on two well-known datasets (MIMIC-III, VitalDB) with ABP and ECG signals, including ablations, sensitivity analysis, and qualitative visualizations.

Major Issues:
• The distinction between “morphological structure” and “amplitude variation” is not clearly defined (e.g. in the abstract as well as in line 46). In some contexts, waveform morphology is largely determined by amplitude dynamics, which risks making the terms redundant. These definitions should be used consistently throughout the manuscript (e.g. “waveform shape” instead of “morphological structure” in line 58).
• The authors introduce a new methodology as well as a new evaluation metric to evaluate the performance of the new methodology. For this newly defined evaluation metric, the new methodology significantly outperforms the state of the art. This becomes particularly problematic when considered together with the missing clear definitions of “morphological structure” and “amplitude variation” which highly influence the newly defined evaluation metric.
• The abstract lacks a statement on the practical relevance of the proposed approach (Why do we need this?).
• Fig. 1 is overly complex:
>> The figure would benefit from being broken down into smaller, more descriptive graphics with clearer structuring.
>> The color coding is unclear.
>> A proper introduction of many variables and abbreviations is missing (HPF, LPF, x, y, etc.) in the caption.
>> Suggestion: It would help to create a more abstract form of the proposed approach and then break it down step by step in the following.
• The authors state that “… was not directly applicable to these models, and a default score of zero was assigned.” (line 175). This strongly biases the evaluation, since assigning a default score of zero artificially favors the proposed model.
• Fig. 2: Radarplots/Spiderplots should not be used because of their potential misinterpretation. Overlapping areas decrease readability, and the visual impression can be misleading since different metrics may have very different importance (which is confirmed by the creation of the newly defined “ISCORE”).
• Fig. 2: The metrics shown are introduced differently in section 3.4: morphological specialization (MS) is defined as I(V;Z |Z) in section 3.4.5. This holds true for the remaining metrics.
• Fig. 3: The meaning of the colormaps is not defined.
• In the discussion the authors state (line 326): “Its [the newly proposed method’s] integration with CMI-based interpretability metrics strengthens structural evaluation while providing clinically actionable insights for real-time patient monitoring, risk stratification, and personalized treatment planning [4, 34, 35].” It is not clear from which characteristics these conclusions can be drawn. A more thorough explanation is needed.
• Section 3.2: The variable names (g_x, g_e etc.) are not indicated in Fig. 1c making it difficult to retrace the model’s components.
• The fidelity metric (FQI) is missing in Fig. 2.
• For the introduction of the ISCORE (line 516) the authors do not state why the ISCORE was computed in this (highly non-linear) way.
• Appendix B – Benchmark comparisons:
>> It seems that the newly developed approach is primarily better because of the MS and AC scores which should be discussed in more detail within the work.

>> Overall, it seems somewhat odd to use only one final metric within the work instead of building an average per base-metric per model. I.e. the computation of the ISCORE seems to be somewhat arbitrary (which is linked to the previous issue).

Minor Issues:
• In the abstract it is unclear whether the proposed method is focused on the generation of synthetic signals based on multiple input entities (e.g. ECG and ABP) or if the model is meant to synthesize from a single input entity that drives the generative process. The confusion is caused by the second sentence where the authors state: “Controlled synthesis of such signals enables …” which reads as the synthesis of ABP and ECG.
• The normalization process of the metrics (line 165) should be clarified.
• Table 1b) shows a set of hyperparameters but only one hyperparameter is displayed (i.e. C=5s instead of C=5s for J=XX and Zeta=YY).
• Fig. 3: There are two different ECG and ABP signals shown for 3 different methods. It seems that each of the ECG/ABP signals belongs to either the MIMIC3 or the VitalDB database but this indication is missing.

Summary:
In summary, while the proposed framework is innovative and technically sophisticated, the evaluation methodology and clarity of presentation require substantial improvement before the claims of superiority over the state of the art can be considered convincing.

(Remarks on code availability)

Reviewer #3

(Remarks to the Author)
I co-reviewed this manuscript with one of the reviewers who provided the listed reports. This is part of the Nature Communications initiative to facilitate training in peer review and to provide appropriate recognition for Early Career Researchers who co-review manuscripts.

(Remarks on code availability)

Version 1:

Reviewer comments:

Reviewer #1

(Remarks to the Author)
No further comments

(Remarks on code availability)

Open Access This Peer Review File is licensed under a Creative Commons Attribution 4.0 International License, which permits use, sharing, adaptation, distribution and reproduction in any medium or format, as long as you give appropriate credit to the original author(s) and the source, provide a link to the Creative Commons license, and indicate if changes were made.
In cases where reviewers are anonymous, credit should be given to 'Anonymous Referee' and the source.
The images or other third party material in this Peer Review File are included in the article's Creative Commons license, unless indicated otherwise in a credit line to the material. If material is not included in the article's Creative Commons license and your intended use is not permitted by statutory regulation or exceeds the permitted use, you will need to obtain permission directly from the copyright holder.
To view a copy of this license, visit https://creativecommons.org/licenses/by/4.0/

General Response to the Editor and All Reviewers (Summary of Revisions)

In response to the reviewers' constructive feedback, we have substantially revised the manuscript to improve clarity, methodological rigor, and overall coherence. Key revisions include updates to **terminology**, **integration of additional PSD estimation methods and averaging schemes for ISCORE computation**, and an expanded Discussion section that provides clearer analyses and **stronger application examples**.

1. Following **Reviewer 2'**s suggestion, the term ***morphology***, which was considered ambiguous and potentially misleading, has been **replaced** with ***waveform shape***, with corresponding updates in related terminology and metrics:
   A. *morphology specialization* → **shape factorization**,
   B. *morphology preservation* → **shape preservation**.

2. Building on **Reviewer 1**'s comment, we incorporated **three complementary** PSD estimation methods, *the Fast Fourier Transform (FFT), Welch-based Spectral Evolution (WSE),* and *Discrete Cosine Transform (DCT)*, to ensure a balanced evaluation across **diverse spectral representations** of the waveform.

3. In response to **Reviewer 2**'s suggestion, we made substantial revisions to establish a more rigorous and equitable evaluation framework (Metrics and ISCORE).
   A. All metrics were **normalized to a 0–1 scale** to ensure comparability across models, using Linfoot's informational correlation coefficient for the CMI-based metrics and the coefficient of determination (R^2) for reconstruction accuracy.
   B. The previous nonlinear **formulation of ISCORE** was replaced with a more **balanced and widely adopted** approach based on **arithmetic, geometric, and harmonic means.**
   C. For diffusion- and WaveNet-based models, the **shape factorization** metric (previously referred to as *morphological specialization*) was **excluded**, rather than being assigned a zero value, to enhance fairness and robustness in comparative evaluation.
   D. All reported figures and tables were substantially revised to reflect these updates.

4. In response to both reviewers' suggestions, we incorporated **a unified, scenario-driven application** that concretely demonstrates VABAM's translational potential across *uncertainty-aware decision support*, *amplitude-targeted augmentation*, and *real-time monitoring* using probabilistic reference bands.

5. The **Discussion section** was further refined to better highlight the model's strong performance in **waveform shape factorization, amplitude modulation controllability**, and **consistent results across the three PSD estimation methods**. The section also strengthens the **application examples**, with figures updated accordingly and the content reorganized and condensed without altering the original meaning.

We also refined the manuscript, including the Introduction, Results, Methods, and Appendix, in accordance with all reviewer comments to ensure clarity and coherence. In addition, the

manuscript underwent professional English polishing, during which several expressions were refined to enhance readability and overall consistency. To facilitate review of the substantial revisions, we provide both a **clean** version and a version with **highlighted changes** as separate files, with relevant manuscript excerpts included directly in the responses. Please **refer** to the reviewers' comments and our detailed **point-by-point responses** below for a comprehensive description of the revisions.

REVIEWER COMMENTS

Reviewer #1 (Remarks to the Author):

- ➔ We sincerely thank Reviewer 1 for the valuable and insightful comments, which have greatly helped improve the quality of the manuscript. Before reading the point-by-point responses, we kindly suggest that the reviewer **first** refer to the "**General Response to the Editor and All Reviewers (Summary of Revisions)**" section above, which provides an overview of the major revisions and will help contextualize the detailed changes presented below.

Comment A.1.1

I have read with real interest the MS entitled: Morphology–Amplitude Decoupling for Pulsatile Physiological Signal Synthesis: A Probabilistic Model and Evaluation Metrics.

It is actually quite frequent, that the morphology of physiological signals may change independently to its amplitude.
The physical process of signal transduction, which constitutes the core part of each biosensor has many cofactors, that typically affect the amplitude of the observed signals. Some changes of amplitude are related to the underlying physical process, and represent the true features of the source of signal, whereas the other are related to the measurement process, sometimes so closely, that it is even hard to refer to them as artifacts or noise - it is just another source of information (unsolicited).
For example loss of amplitude of blood pressure may be related with acute haemorrhage, orthostatic intolerance, external acceleration, but also to external pressure applied to sensor or to measured object, or sudden change of geometry. In case of photoplethysmography it is sufficient to tilt the diode. In case of the ECG there are even more possibilities, as electrode contact, skin humidity (e.g. bridging ECG electrodes using conducting gel), galvanic skin response, electrode tension, electrode motion or water immersion of the extremity may affect the ECG, having nothing to do with the actual state of the source. Therefore, it is important to distinguish the changes related to the source (substantial) from those that are caused by parameters of the measurement process itself (non substantial).

- ➔ Thank you for your thoughtful and detailed comment. We genuinely appreciate your insight into the distinct sources of variation in physiological signals—particularly the distinction between substantial (source-related) and non-substantial (measurement-related) changes. At the same time, we note that both waveform amplitude and shape can be influenced by multiple physiological and measurement-related factors. Therefore, while we greatly appreciate the substantial–non-substantial categorization you mentioned, we emphasize in our work that these two components carry distinct types of information.
- ➔ This perspective has provided valuable inspiration for strengthening our work's foundational motivation. In particular, the view that amplitude and shape convey different kinds of information reinforces the necessity of explicitly decoupling these components in our generative framework.
- ➔ Drawing upon this perspective, we have clarified this motivation in the Introduction by

adding the following text (**page 2, line 51**):

“Physiologically, waveform shape and amplitude represent distinct information sources: shape reflects the dynamic processes and system properties governing signal generation and propagation, while amplitude encodes the quantitative state of the underlying variable [guyton2011, rangayyan2024]. This distinction calls for generative frameworks specifically designed to capture domain-specific signal properties.”

Reference:

Guyton, A.C., Hall, J.E. Guyton and Hall Textbook of Medical Physiology. Elsevier, Philadelphia, PA (2011).

Rangayyan, R.M., Krishnan, S. Biomedical Signal Analysis. John Wiley & Sons, Hoboken, NJ (2024)

Comment A.1.2

The assumption, that one can disentangle the changes of morphology and the changes of amplitude is true only in the first order of approximation. For instance, increase of systolic blood pressure increases the pressure gradient between the arterial and the venous component, which affects the AV outflow. This, in turn, affects the time decrement of the downward slope od the ABP curve, There are many more effects of this type, such as e.g. emergence of the second dichrotic notch and so on. Note also, that in the specific case of ABP, its amplitude actually rises along the vascular tree - this fact was used to build the models, that allow reconstruction of central pressure (e.g. https://patents.google.com/patent/WO2023152546A1/en, https://www.sciencedirect.com/science/article/abs/pii/S1746809422000350)

- ➔ We appreciate the reviewer’s valuable comment regarding the assumption that “*waveform shape and amplitude variations can be fully disentangled*”. We agree that this assumption holds only as a first-order approximation. In response, we have **revised** the Limitations section to **explicitly acknowledge this point** (**page 15, line 387**) by adding the following sentence:

“Additionally, VABAM assumes that shape and amplitude variations are separable---a simplification that may not fully capture higher-order coupling effects, particularly physiological shape–amplitude coupling [xiao2022, MirotaPopiel2023]. This assumption may limit physiological realism in modeling complex waveform dynamics, underscoring the need for future architectures that better represent their interdependence.”

Reference:

Xiao, H., Liu, C. & Zhang, B. Reconstruction of central arterial pressure waveform based on CNN–BiLSTM. Biomedical Signal Processing and Control 74, 103513 (2022)

Mirota, K. & Popiel, I. Reconstruction of a patient-specific central arterial pressure waveform morphology from a distal non-invasive pressure measurement. WO2023152546A1. World Intellectual Property Organization (2023)

Comment A.1.3

Methods that build a digital representation of signals emerge in this context as important research

tools, that allow to distinguish between substantial and non-substantial changes. Error of reconstruction may be used as a valuable research tool, which quantifies the amount of our ability to reconstruct the process based on the a priori knowledge (c.f. https://bamsjournal.com/article/551381/en).
Parameters of the latent space constitute the language for interpretation of the data, which can be used to construct clinical indices.

- ➔ We appreciate this thoughtful comment and agree that digital signal representations and reconstruction error provide meaningful ways to capture substantial changes. We also agree that incorporating *a priori* domain knowledge could further enhance interpretability. This perspective has been explicitly reflected in **the Discussion** section (**page 15, line 385**):

"Future work could address this limitation by incorporating domain-adaptive priors or spectrum-aware regularization to improve generalization."

Comment A.2

It is rare to find a paper that may be accepted "as is", a few minor comments below.

p2 line 43 - the ECG does not convey the information on hemodynamic state, rather the information on the electric activity of the cardiac effector

- ➔ We thank the reviewer for this helpful comment. In accordance with the suggestion, we have revised the description to clarify that the ECG represents electrical, rather than hemodynamic, activity (**page 2, line 40**):

"These include arterial blood pressure (ABP) waveforms and photoplethysmogram (PPG), which convey rich information about underlying hemodynamic states, and electrocardiogram (ECG), which reflects the electrical activity of the cardiac effector."

Comment A.3.1

Note, that if you use PSD calculation as one of steps of the process, you expose yourself to all kinds of errors related to PSD estimation. The PSD estimation method is not disclosed, or I was unable to find it. Note, that, if the FFT was used, it works in unfavourable conditions, as it has to represent the delta-spike signal, which is locally nonstationary.

- ➔ We appreciate this important comment regarding potential biases introduced by PSD estimation. In the initial version of the manuscript, only the basic Fast Fourier Transform (FFT) method was used. In the revised version, and building on your valuable feedback, we have expanded the evaluation to include three complementary PSD estimation methods: **Fast Fourier Transform** (**FFT**), **Welch-based Spectral Evolution (WSE)**, and **Discrete Cosine Transform (DCT)**, to enhance methodological rigor and robustness. Detailed responses and corresponding revisions are provided under Comment A.3.3 below.

Comment A.3.2

Note, that you did not mention the phase part of the spectrum.

- ➔ We thank you for this valuable observation. The omission of phase information in the original manuscript reflects the focus of this study, which aimed to establish a probabilistic evaluation framework centered on amplitude modulation fidelity within a fixed waveform shape.
- ➔ Within this scope, the phase part was not considered, but we acknowledge its importance and plan to address it in future extensions of the framework. Accordingly, we have revised the ***Methods*** and ***Discussion*** sections to **acknowledge this point**:

"In this approach, phase information is not considered, as the objective, which is to quantify the fidelity of amplitude separation from waveform shape, requires only amplitude-wise random variables for probabilistic modeling." (**page 18, line 495**)

"Beyond addressing these limitations, several additional directions could further extend VABAM's capabilities. These include expanding the current amplitude-based modulation to incorporate phase-wise control, enabling finer temporal manipulation and improved modeling of physiological variability, along with the integration of phase-aware metrics into the assessment framework." (**page 15, line 395**)

Comment A.3.3

There exists methods that are alternative to the windowed FFT, e.g. Matching Pursuit - c.f. https://sparse-plex.readthedocs.io/en/latest/book/pursuit/mp/algorithm.html or evolutionary periodogram (Welsh). A comment on this would be favourable. Note, that building Fourier representation is also an optimization problem.

- ➔ We thank you for this valuable comment. In the initial version, only the Fast Fourier Transform (FFT) was used for spectral estimation. Building on your thoughtful suggestion, we extended our evaluation to include three complementary PSD estimation methods: **Fast Fourier Transform (FFT)**, **Discrete Cosine Transform (DCT),** and **Welch-based Spectral Evolution (WSE).**
- ➔ Regarding Matching Pursuit (MP), we initially implemented the algorithm in the revision for preliminary testing. However, in our large-scale evaluation involving over 10,000 observations, each requiring more than 100 repeated spectral samples, MP imposed excessive computational cost. As a practical solution, we adopted the **Discrete Cosine Transform (DCT)** as an efficient and stable alternative that preserves the key property of spectral sparsity while remaining fully compatible with our framework. Although there is no direct theoretical equivalence between DCT and MP, previous studies have proposed **DCT as a sparse spectral baseline** when computational constraints limit the applicability of adaptive decomposition methods such as MP (https://doi.org/10.1016/j.displa.2023.102396).
- ➔ Regarding **WSE**, we interpreted the reviewer's comment to an "evolutionary periodogram (Welch)" as combining the conventional Welch method (10.1109/TAU.1967.1161901) with a time-varying spectral representation. As no

standardized formulation or TensorFlow[1]-compatible implementation exists for this approach, we realized it using a **Short-Time Fourier Transform (STFT)** function in scipy.signal[2], computing overlapping segment-wise spectra and averaging their squared magnitudes to obtain the PSD. Although this implementation may not exactly match the "evolutionary periodogram (Welch)", it preserves the essential characteristics of Welch-based spectral evolution while remaining numerically stable and fully integrable with our framework. The resulting spectral patterns were consistent with FFT and DCT results, confirming the robustness of the implementation.

Comment A.4

p.5 line 122 (**now page 5, line 130**) When you mention segmentation, you don't mention the method for segment definition. Are they based on peak detection and segmented like in the ECG analysis? A comment would be helpful.

- ➔ We appreciate this helpful comment. The segments mentioned in our manuscript do not correspond to waveform cycles defined by peak–valley detection or start–end alignment, as in ECG analysis. Instead, VABAM operates on fixed-length 10-second windows sampled at 100 Hz (1,000 data points per sample), allowing the model to process continuous signals flexibly and efficiently without explicit cycle segmentation.
- ➔ To avoid confusion, we have **replaced** the term ***segments*** with ***samples*** throughout the manuscript, as this more accurately reflects our dataset composition (training, validation, and test sets) rather than morphological segmentation. We sincerely thank the reviewer for highlighting this important clarification.

Comment A.5

A choice of theta acute is questionable, as the comma is located on top of the greek letter which is poorly readable. This problem first appears in p. 6 line 186 and 189, but it is also related to different symbols - e.g. Z and z in p. 15 line 432 - note that Z with such a placement of an accent is a regular letter in some languages. If the accent was shifted past the symbol, the readability of text would be much improved.

- ➔ We thank the reviewer for this careful observation regarding the readability of accented symbols such as $\acute{\theta}$ and $\acute{z}$. We experimented with several alternatives, including shifting the accent after the symbol (θ' and z'), as suggested. Among the tested options, replacing **the acute accent with a hat symbol** (e.g., $\hat{\theta}$ and $\hat{z}$) provided noticeably better readability and visual consistency across all occurrences. We have therefore adopted this notation consistently throughout the revised manuscript. However, if you prefer the shifted-accent format, we will be pleased to implement that adjustment in the next revision.

---

[1] The deep learning development library used for VABAM.

[2] The signal processing module in the SciPy library.

Comment A.6

Although you draw conclusions concerning the potential of VABAM in biomedical application, there is no example of application, that could sparkle the imagination - note e.g. the approach taken
here: https://www.sciencedirect.com/science/article/pii/S0022073622002138?via=ihub wher e an average ECG is displayed as a reference - the commercial tool that utilizes these results may also be of help to you concerning future commercialization path.
https://cineecg.com/

- ➔ We sincerely thank the reviewer for this insightful suggestion. We fully agree that the previous version described the biomedical potential of VABAM only conceptually, without providing a concrete example that could inspire clinical imagination. Following your recommendation, and drawing inspiration from the references you kindly shared, we have now developed **a unified, scenario-driven application** that demonstrates how VABAM may operate within real-world clinical contexts.
- ➔ In the revised manuscript, we newly present a cohesive application framework. This illustration concretizes VABAM's translational potential through three representative scenarios.
  - **Uncertainty-aware decision support:** VABAM can provide uncertainty bounds, enabling clinicians to assess prediction confidence and refine decisions beyond single deterministic outputs.
  - **Amplitude-targeted augmentation:** VABAM augments physiologically plausible low-amplitude variants to better predict adverse physiological events such as intraoperative hypotension.
  - **Real-time monitoring with probabilistic reference bands:** By constructing augmented reference distributions from the preceding signal window, VABAM defines expected variability ranges and continuously tracks deviations from these probabilistic envelopes.
- ➔ This illustration transforms the previously abstract concept into a concrete workflow, demonstrating how VABAM can serve as a generative foundation for real-time, uncertainty-aware clinical monitoring.

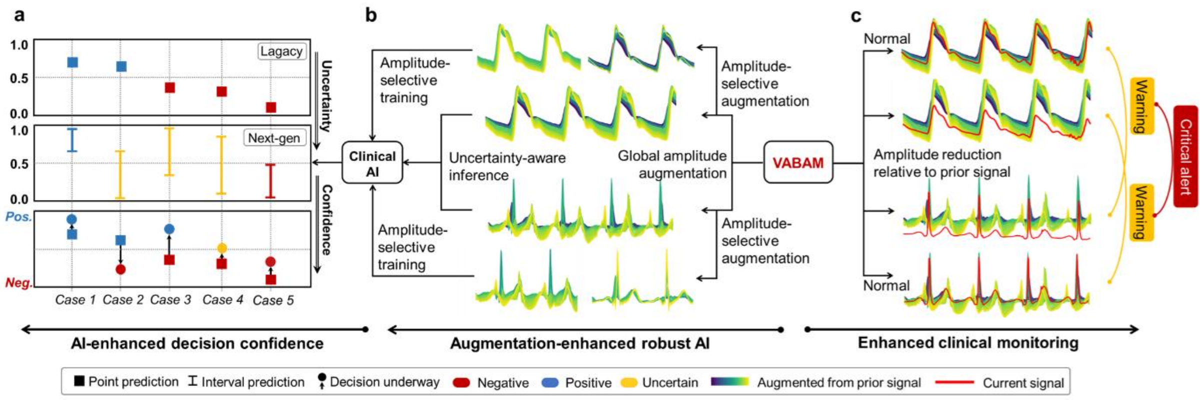

This figure has been newly added to illustrate a representative application of VABAM **(Fig. 8 on page 14)**.

➔ Accordingly, the corresponding content has been added to the ***Discussion*** section (**page 14, line 368**):

"In practice, VABAM has the potential to unify predictive modeling, uncertainty quantification, and clinical interpretation. It generates physiologically plausible waveform variants tailored to specific clinical scenarios, such as lower amplitude–targeted augmentations to more sensitively assess hypotensive risk during surgery (Figure 8b) [hwang2023]. These augmented ensembles capture both physiological variability and **predictive uncertainty**, providing a richer and more informative basis for precise and reliable clinical decision-making (Figure 8a) [kompa2021]. Within this **uncertainty-aware** framework, VABAM supports **real-time monitoring** by generating **reference bands** (Figure 8c), against which incoming waveforms are continuously compared. Departures in a single signal often reflect transient artifacts, while consistent deviations across multiple consecutive signals, rare under stable physiology, suggest genuine physiological disturbances [imhoff2006, koch2019, aboukhalil2008]. Unlike conventional methods that aggregate multiple waveform cycles to establish the reference bands [proniewska2023], VABAM constructs them from a single preceding observation by generating multiple amplitude-varied augmentations, thereby improving both computational efficiency and early-warning sensitivity."

Reference:

Hwang, E., Park, Y.-S., Kim, J.-Y., Park, S.-H., Kim, J., Kim, S.-H.: Intraoperative hypotension prediction based on features automatically generated within an interpretable deep learning model. IEEE Transactions on Neural Networks and Learning Systems (2023).

Kompa, B., Snoek, J., Beam, A. L.: Second opinion needed: communicating uncertainty in medical machine learning. NPJ Digital Medicine 4(1), 4 (2021).

Imhoff, M., Kuhls, S.: Alarm algorithms in critical care monitoring. Anesthesia & Analgesia 102(5), 1525–1537 (2006).

Koch, E., Lovett, S., Nghiem, T., Riggs, R. A., Rech, M. A.: Shock index in the emergency department: utility and limitations. Open Access Emergency Medicine, 179–199 (2019).

Aboukhalil, A., Nielsen, L., Saeed, M., Mark, R. G., Clifford, G. D.: Reducing false alarm rates for critical arrhythmias using the arterial blood pressure waveform. Journal of Biomedical Informatics 41(3), 442–451 (2008).

Proniewska, K. K., Abächerli, R., Dam, P. M.: The δwave ECG: the differences to the normal 12-lead ECG amplitudes. Journal of Electrocardiology 76, 45–54 (2023).

Comment A.7

Reviewer #1 (Remarks on code availability):
I did not run the code, although I can see, that the instructions are very clear and the quality of the digital part of submission is outstanding, as compared to typical open source projects.

➔ We sincerely thank for this generous and encouraging comment.

Reviewer #2 (Remarks to the Author):

Summary:
The manuscript introduces a novel method (called VABAM) for the generation of synthetic physiological signals that relies on cascaded filtering and that is meant to structurally separate morphology and amplitude in pulsatile physiological signals.

Strengths:
• The cascaded filtering approach and variational design are innovative and carefully described. The mathematical formulation is rigorous, and the architecture appears well thought out.
• The method is tested on two well-known datasets (MIMIC-III, VitalDB) with ABP and ECG signals, including ablations, sensitivity analysis, and qualitative visualizations.

- ➔ We sincerely thank **Reviewer 2** for the valuable and insightful comments, which have greatly helped us improve the quality of the manuscript. Before reading the point-by-point responses, we kindly suggest that the reviewer **first** refer to the "**General Response to the Editor and All Reviewers (Summary of Revisions)"** presented on page 1, which provides an overview of the major revisions and will help in understanding the overall context of our changes.

Major Issues:

Comment B.1

• The distinction between "morphological structure" and "amplitude variation" is not clearly defined (e.g. in the abstract as well as in line 46). In some contexts, waveform morphology is largely determined by amplitude dynamics, which risks making the terms redundant. These definitions should be used consistently throughout the manuscript (e.g. "waveform shape" instead of "morphological structure" in line 58).

- ➔ We appreciate this important comment. To avoid potential ambiguity and to fully reflect your suggestion, we have **replaced** all previously used **vague terms** such as *morphology or morphological structure* with *waveform shape* throughout the manuscript and abstract to ensure clarity and consistency.
- ➔ Additionally, we **added** a concise **definition in the *Introduction*** distinguishing *waveform shape* from *amplitude variation* (**page 2, line 48**):

"**Waveform shape** typically refers to the temporal arrangement of onset and offset points, peaks, and valleys within a cycle, while **amplitude variation** denotes changes in signal magnitude independent of shape [guyton2011, rangayyan2024]."

Reference:

Guyton, A.C., Hall, J.E. Guyton and Hall Textbook of Medical Physiology. Elsevier, Philadelphia, PA (2011).

Rangayyan, R.M., Krishnan, S. Biomedical Signal Analysis. John Wiley & Sons, Hoboken, NJ (2024)

Comment B.2

• The authors introduce a new methodology as well as a new evaluation metric to evaluate the performance of the new methodology. For this newly defined evaluation metric, the new methodology significantly outperforms the state of the art. This becomes particularly problematic when considered together with the missing clear definitions of “morphological structure” and “amplitude variation” which highly influence the newly defined evaluation metric.

- ➔ We appreciate this critical comment. As noted in our response to Comment B.1, we have carefully addressed this concern by **providing clear definitions of *waveform shape* and *amplitude variation*** in the ***Introduction*** (**page 2, line 48**) and replacing the previously ambiguous term *morphological structure* with *waveform shape* throughout the manuscript, thereby ensuring the conceptual basis of the newly defined evaluation metric is well grounded and unambiguous.

Comment B.3

• The abstract lacks a statement on the practical relevance of the proposed approach (Why do we need this?).

- ➔ We thank the reviewer for this constructive feedback. We have revised the abstract to explicitly highlight the practical relevance of VABAM. In the original abstract, we included generic downstream tasks such as "waveform generation, modality translation, and signal restoration," which, while technically accurate, did not effectively convey the practical value that VABAM can offer. Therefore, we have removed this list of generic tasks and replaced it with a **concrete statement describing the specific clinical applications** enabled by our approach.
- ➔ Based on the applications discussed in the Discussion section, we have added the following statement at the **end of the abstract**:

"This may enable amplitude-targeted augmentation, uncertainty-quantified prediction, and enhanced real-time anomaly monitoring, thereby advancing clinical decision-making in physiological signal analysis. "

- ➔ We believe this addition clarifies the impact of VABAM and aligns the abstract more closely with the practical motivations and implications emphasized throughout the manuscript.

Comment B.4

• Fig. 1 is overly complex:
>> The figure would benefit from being broken down into smaller, more descriptive graphics with clearer structuring.
>> The color coding is unclear.
>> A proper introduction of many variables and abbreviations is missing (HPF, LPF, x, y, etc.) in the caption.
>> Suggestion: It would help to create a more abstract form of the proposed approach and then break it down step by step in the following.

➔ We appreciate this constructive suggestion. Following your guidance, we have completely redesigned Figure 1. The original figure has been divided into two separate and more descriptive visuals:

- **Figure 1** now presents an abstract and intuitive overview of the VABAM components and structure. (**page 3**)
- **Figure 2** provides a step-by-step, visually simplified explanation of the evaluation metrics. (**page 4**)

➔ This restructuring removes unnecessary complexity and improves readability. We also added a color map to clarify the meaning of each heatmap color and provided clear abbreviations and definitions for all variables and terms (e.g., HPF, LPF, z, θ, π, etc.) in the corresponding captions (**page 3 and 4**).

➔ Overall, the revised figures provide a more intuitive and accessible representation of both the model and the evaluation process. The two newly revised figures have been added as:

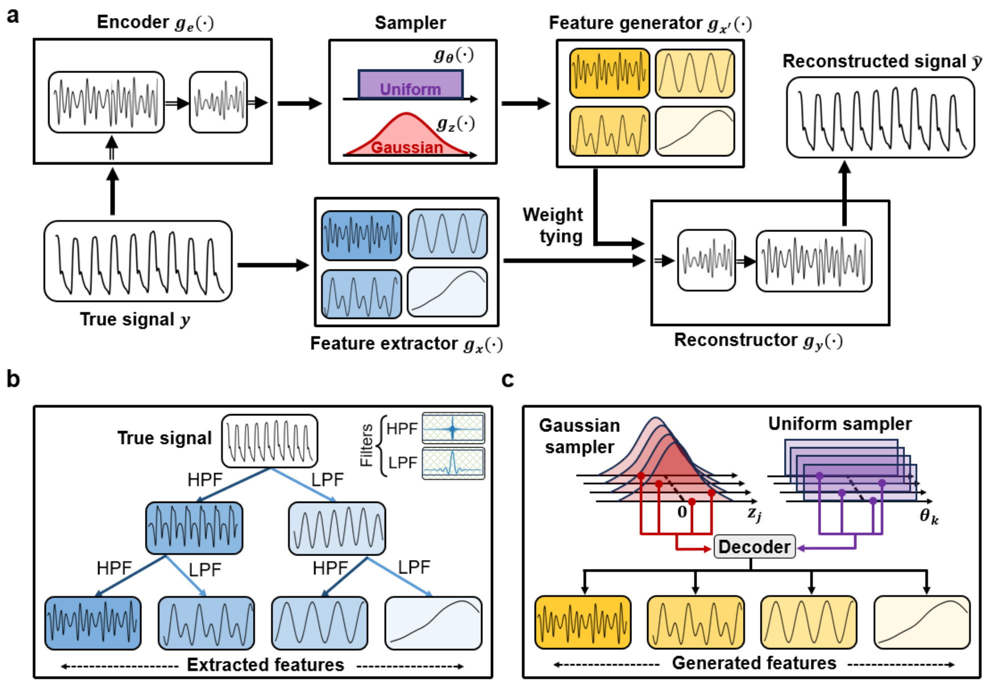

This figure has been newly added as **Figure 1**, providing an abstract overview of the VABAM framework.

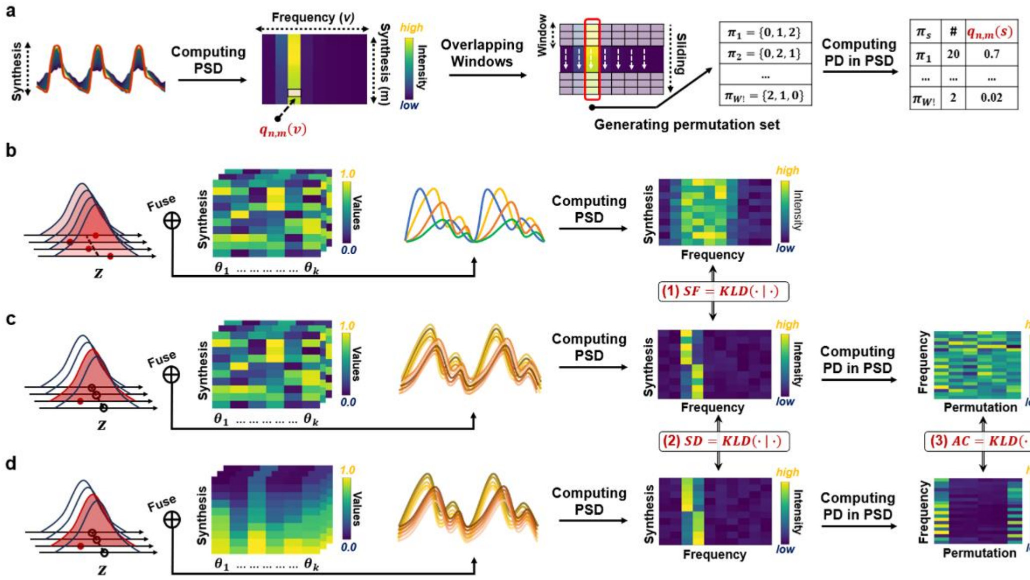

This figure has been newly added as **Figure 2**, offering a simplified and intuitive explanation of the evaluation metrics.

Comment B.5

• The authors state that "... was not directly applicable to these models, and a default score of zero was assigned." (line 175). This strongly biases the evaluation, since assigning a default score of zero artificially favors the proposed model.

- ➔ We appreciate this important observation and fully acknowledge your concern that assigning a default score of zero could introduce bias into the evaluation. In the revised version, we **no longer assign** a default score **of zero** to any model. Specifically, **VAE-based models and VABAM** are evaluated using **five metrics**, including *shape factorization* (previously referred to as *morphological specialization*), while **diffusion- and WaveNet-based models** are evaluated using **the remaining four metrics**, excluding *shape factorization*.
- ➔ This adjustment was made possible because the ***ISCORE*** **formulation** has been completely **revised**. We replaced the previous non-linear scheme with a balanced **averaging approach** (arithmetic, geometric, and harmonic means), enabling consistent and unbiased comparison regardless of whether four or five metrics are used. Accordingly, we also provide *ISCORE* tables based on **four metrics for VABAM** and the **diffusion/WaveNet models**, ensuring that their comparison is performed on an equivalent and unbiased basis (**page 24-26**).
- ➔ For clarity, in the main results figure, VABAM's ISCORE is computed as an aggregate over all five metrics (**page 8, Figure 3**), as the introduction of the waveform shape factorization metric constitutes an important part of this study. Moreover, since VABAM belongs to the VAE-based family, its most meaningful comparison is within that group. To complement this aggregate view and reinforce the robustness of our evaluation, we additionally present detailed per-metric results (**page 9, Figure 4**).

- ➔ Accordingly, the main text has been revised to reflect these changes and to ensure full consistency between the evaluation procedure, figures, and reported results. In particular, the section *"Strategies for quantitative evaluation"* in the ***Results*** has been revised as follows (**page 6, line 182**):

"Unlike VABAM and VAE-based architectures, diffusion-based and autoregressive models do not employ explicitly disentangled representations [oord2016, kong2021, kingma2021]. Their outputs are instead governed by holistic latent trajectories or global conditioning mechanisms. Because the waveform shape factorization metric depends on factorized latent manipulations, it was not directly applicable to these models and was therefore excluded from ISCORE computation. Accordingly, these models were evaluated using four metrics. To ensure fair comparison, our model---although primarily assessed using all five metrics---was additionally evaluated with the same four metrics as the diffusion-based and autoregressive models."

Reference:

Oord, A., Dieleman, S., Zen, H., Simonyan, K., Vinyals, O., Graves, A., Kalchbrenner, N., Senior, A., Kavukcuoglu, K.: Wavenet: A generative model for raw audio. In: Speech Synthesis Workshop (SSW) (2016)

Kong, Z., Ping, W.: Diffwave: A versatile diffusion model for audio synthesis. In: International Conference on Learning Representations (ICLR) (2021)

Kingma, D., Salimans, T., Poole, B., Ho, J.: Variational diffusion models. Advances in Neural Information Processing Systems 34, 21696–21707 (2021)

- ➔ Additionally, the section *"Quantitative results on benchmark tasks"* now reports both cases, using all five metrics and using four metrics, to provide a fair and transparent comparison across model families (**page 7, line 214**):

"VABAM achieved the highest ISCORE across datasets and signal types, substantially outperforming benchmark models under all averaging schemes, except for ECG under DCT, where diffusion-based models performed better. When all five metrics were included, VABAM ranked first in 11 of the 12 configurations based on the GM, with relative differences from the best-performing alternative ranging from -17.4% to +113.4% (Figure 3). When four metrics were considered---excluding waveform shape factorization---performance remained competitive, again ranking first in 11 of the 12 configurations (Tables B1–B3)"

Comment B.6

• Fig. 2: Radarplots/Spiderplots should not be used because of their potential misinterpretation. Overlapping areas reduce readability, and the visual impression can be misleading because different metrics may vary significantly in their relative importance, as also reflected in the introduction of the newly defined ISCORE.
• Fig. 2: The metrics shown are introduced differently in section 3.4: morphological specialization (MS) is defined as I(V;Z |Z) in section 3.4.5. This holds true for the remaining metrics.

- ➔ We appreciate this constructive comment. In the revised version, all radar/spider plots have been removed and **replaced** with **bar charts and line plots** to enhance readability

and prevent potential misinterpretation.

➔ For clarity, as mentioned in the "*General Response to the Editor and All Reviewers (Summary of Revisions)*", we expanded the evaluation to include **three PSD methods** (FFT, DCT, and WSE). In addition, **ISCORE** was computed using **three averaging schemes**—arithmetic (AM), geometric (GM), and harmonic (HM) means—to address *Comment B.11*. Accordingly, **four new figures** reflecting these updates have been added in this revision for reference:

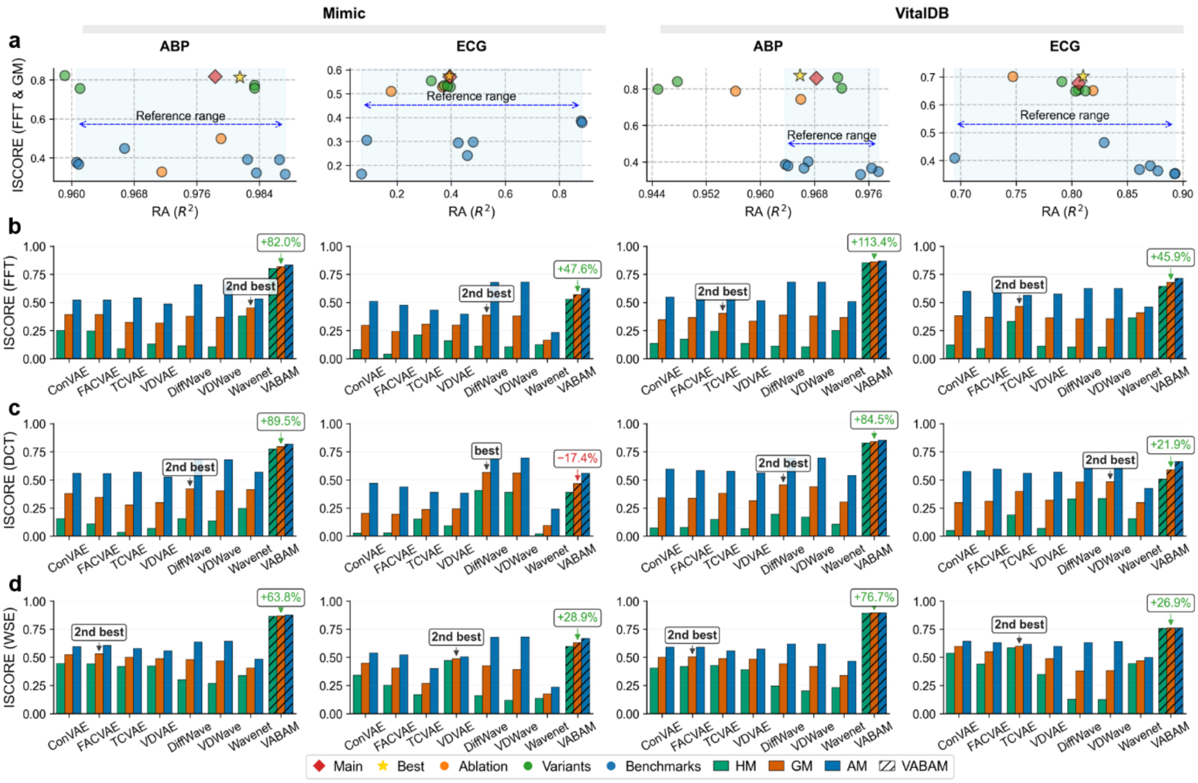

This figure has been newly added as **Figure 3**, offering an integration-level evaluation that compares generative performance across models and PSD methods, showing that our model consistently achieves excellent ISCORE values across datasets and signal types (**page 8, figure 3**).

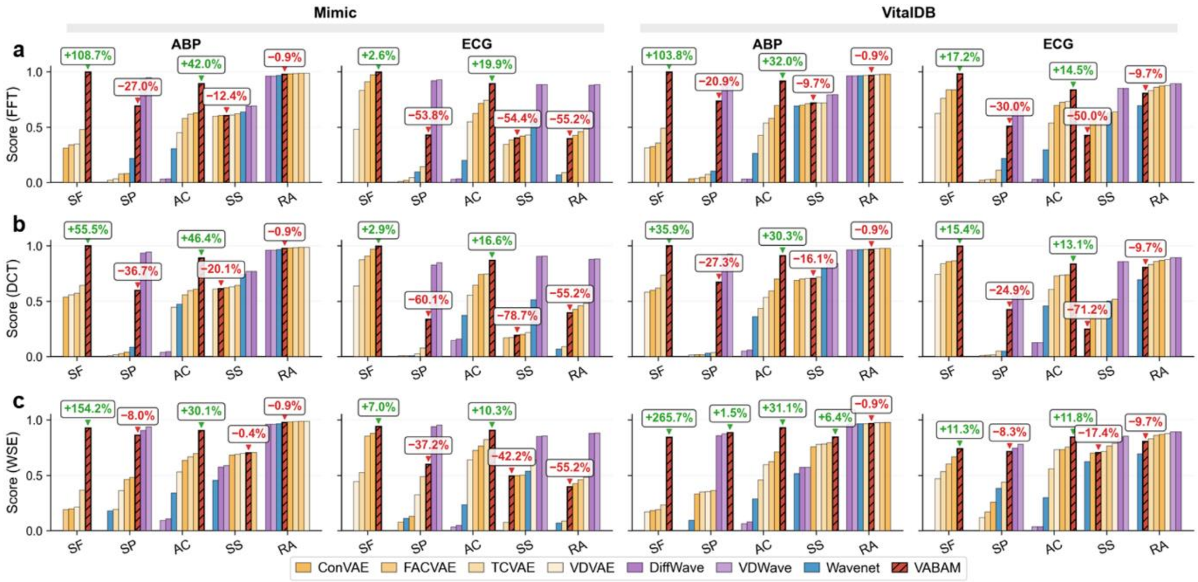

This figure has been newly added as **Figure 4**, presenting a metric-level evaluation that compares models across five quantitative metrics—*waveform shape factorization (SF), shape preservation (SP), amplitude modulation controllability (AC), spectral similarity (SS)*, and *reconstruction accuracy (RA)* (**page 9, figure 4**). The results demonstrate that our method achieves clearly superior performance in SF and AC, and maintains at least intermediate performance on the remaining three metrics, showing inferior results only in a few cases.

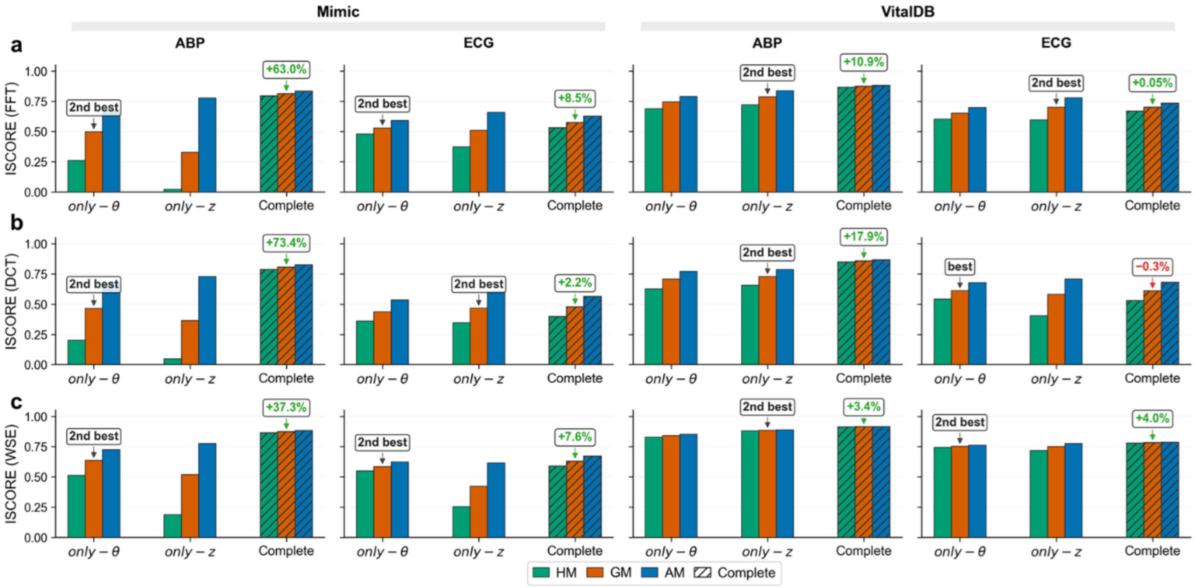

This figure has been newly added as **Figure 5**, presenting ablation studies that compare ablated and complete models. The results show that the complete model achieves the highest harmonic, geometric, and arithmetic mean scores across datasets and signal types (**page 10, figure 5**).

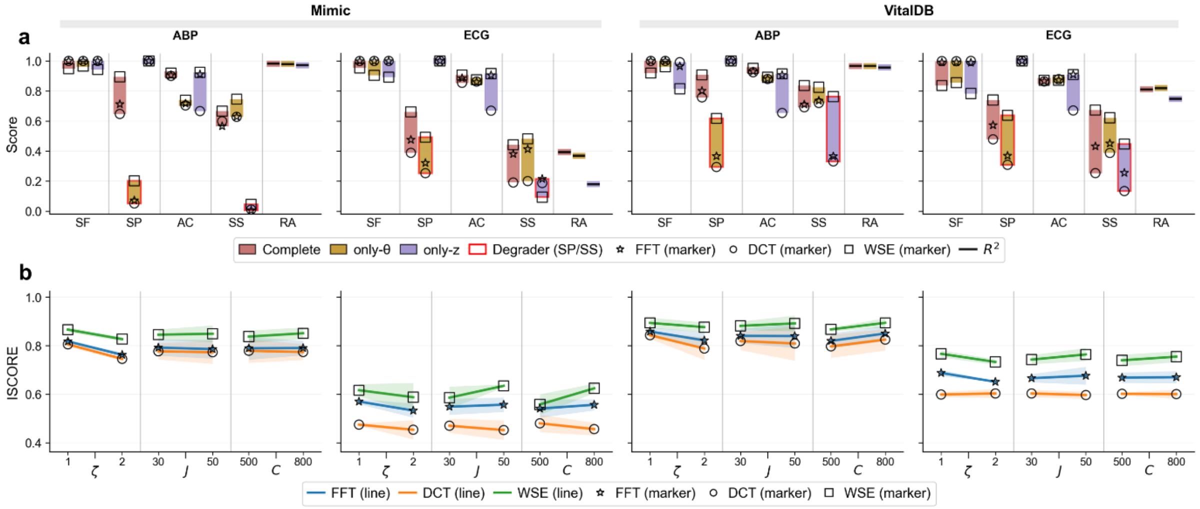

This figure has been newly added as **Figure 6**, presenting performance range and sensitivity analyses (**page 11, figure 6**). The results show that the complete model maintains stability across metrics, with no metric acting as a critical performance degrader (a). In panel (b), variations in filter depth ζ, latent dimension J, and feature length C reveal overall consistency across PSD estimation methods, with minor deviations.

Comment B.7

• Fig. 3: The meaning of the colormaps is not defined.

➔ We appreciate the reviewer's careful observation. The current **Figure 7** corresponds to the **previous Figure 3**. In the revised version, we have supplemented the explanation of the colormaps to clarify their meaning and improve interpretability. Specifically, we added labels and tick values (minimum and maximum) to the colormap axis within the figure, and we have briefly described in the caption that the colormaps represent the magnitudes of the conditioning variables (**page 12, figure 7**).

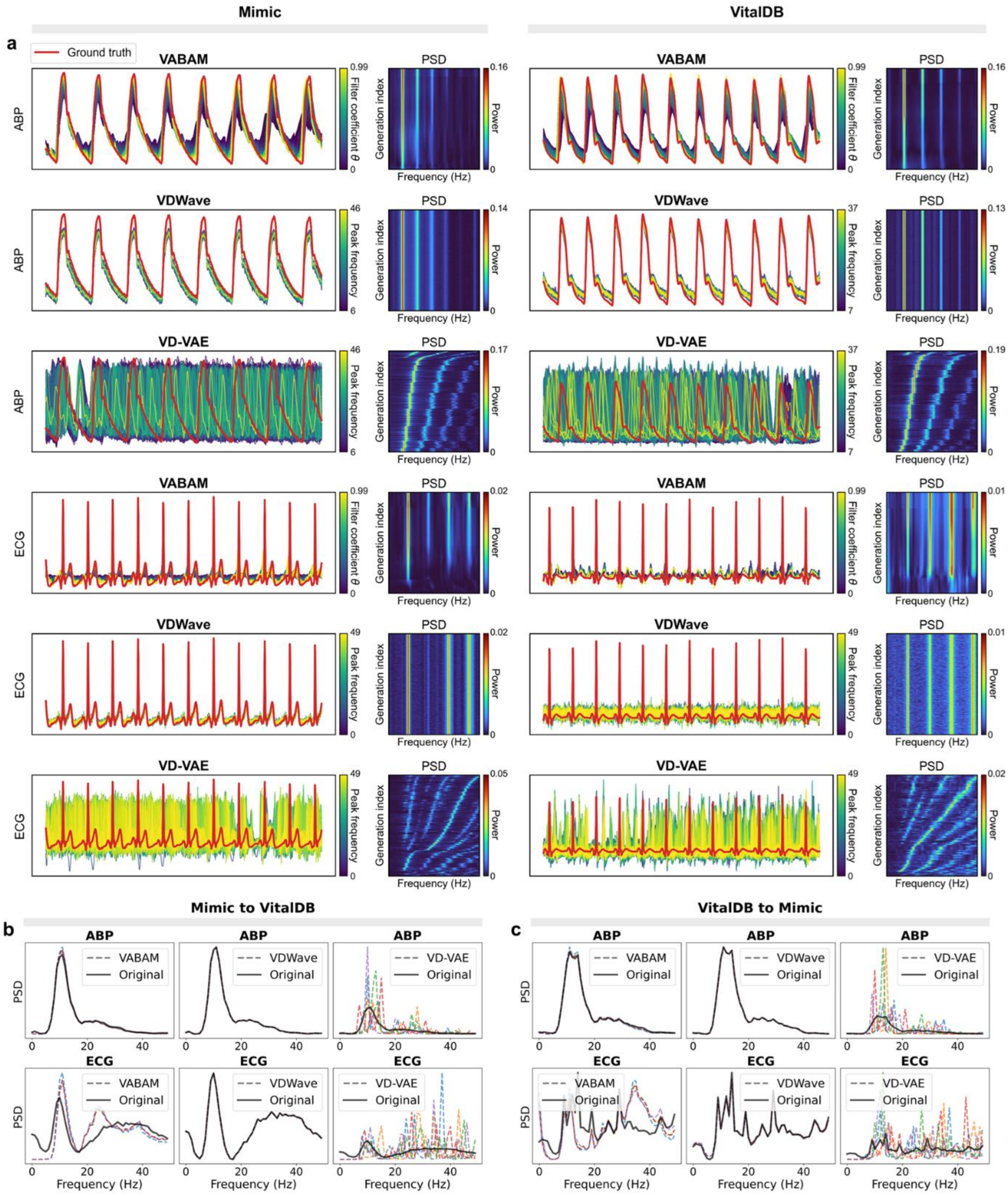

Comment B.8

• In the discussion the authors state (line 326): "Its [the newly proposed method's] integration with CMI-based interpretability metrics strengthens structural evaluation while providing clinically actionable insights for real-time patient monitoring, risk stratification, and personalized treatment planning [4, 34, 35]." It is not clear from which characteristics these conclusions can be drawn. A more thorough explanation is needed.

➔ We sincerely thank the reviewer for this insightful comment. We fully acknowledge that the original discussion was too general and did not sufficiently explain how the proposed model leads to the stated clinical implications. Reviewer 1 also kindly suggested relevant reference papers to strengthen the application perspective, which prompted a thorough revision of this section.

➔ Motivated by this feedback, we removed the earlier, general statements and completely rewrote the section. In the revised manuscript, we developed a **unified, scenario-driven application framework** that concretely demonstrates how **VABAM** can function in real-world clinical contexts. Accordingly, the section in the ***Discussion*** has been rewritten to clearly establish VABAM's clinical relevance through specific, scenario-based evidence (see **Fig. 8** and Discussion on **page 14, line 368**).

➔ The framework illustrates three representative use cases:

- **Uncertainty-aware decision support:** VABAM can provide uncertainty bounds, allowing clinicians to assess prediction confidence and refine their decisions beyond single deterministic outputs.
- **Amplitude-targeted augmentation:** VABAM augments physiologically plausible low-amplitude variants to better predict adverse physiological events such as intraoperative hypotension.
- **Real-time monitoring with probabilistic reference bands:** By constructing augmented reference distributions from the preceding signal window, VABAM defines expected variability ranges and continuously monitors deviations from these probabilistic envelopes.

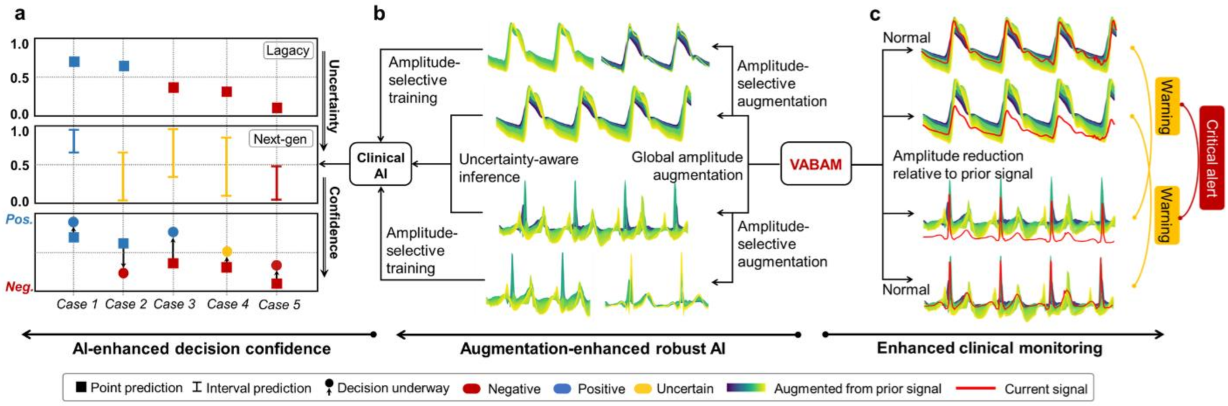

This figure has been newly added to illustrate a representative application of VABAM **(page 14, figure 8)**.

Comment B.9

• Section 3.2: The variable names (g_x, g_e etc.) are not indicated in Fig. 1c making it difficult to retrace the model's components.

- ➔ We thank the reviewer for pointing this out. In the revised version, all variable names (e.g., g_x, g_e, g_z, and g_θ) have been added to the schematic for clarity and to ensure correspondence with the text. Please refer to the newly redrawn **Figure 1**, introduced in **Comment B.4**, which provides a clearer mapping between the notations and the model components.

Comment B.10

• The fidelity metric (FQI) is missing in Fig. 2.

- ➔ We appreciate the reviewer's observation. The previously referenced *Figure 2* has been reorganized and now corresponds to the current *Figures 3, 4,* and *6*.
- ➔ As an additional clarification, in this revision, the **filtering quality index (FQI)** has been inverted (1 – correlation) to derive the **spectral similarity** metric. We focused our reporting on **spectral similarity** to ensure that all quantitative metrics are presented in a consistent direction, where values closer to 1 indicate superior generative performance. Therefore, FQI is not reported separately.

Comment B.11

• For the introduction of the ISCORE (line 516) the authors do not state why the ISCORE was computed in this (highly non-linear) way.

- ➔ We appreciate the reviewer's insightful comment. In the earlier version, we had indeed focused on developing a novel integrated metric, as not all evaluation metrics were applicable to every model and their numerical ranges varied substantially. This led us to design a custom, non-linear formulation intended to yield positive-valued scores across conditions. We now fully **acknowledge** this approach was **unnecessarily complex** and **insufficiently justified**.
- ➔ Following your valuable suggestion, we have **removed the previous non-linear computation** and now **define ISCORE** using three standard **averaging schemes**—arithmetic, geometric, and harmonic means, providing a transparent and fair integration across the five metrics. We are sincerely grateful for this important comment, which has significantly strengthened the rigor and clarity of our evaluation framework.
- ➔ Additionally, we have incorporated a brief explanation in the ***Introduction*** to **clarify the rationale behind ISCORE** and to emphasize its role in integrating multiple metrics into a unified and balanced evaluation framework (**page 5, line 111**):

"All the five metrics are integrated into a single composite score (ISCORE), which provides a unified evaluation of generative performance. ISCORE captures balanced performance across trade-offs that often arise among individual metrics, enabling consistent model comparisons and offering a holistic reflection of both structural quality and generative fidelity."

Comment B.12

• Appendix B – Benchmark comparisons:

>> It seems that the newly developed approach is primarily better because of the MS and AC scores which should be discussed in more detail within the work.

- ➔ We thank the reviewer for this valuable comment and agree that the observed performance gains are largely attributed to improvements in waveform shape factorization (SF; previously referred to as morphological specialization, MS) and amplitude modulation controllability (AC). To address this point, we have **added a paragraph in the *Discussion* section** elaborating on these aspects (**page 13, line 338**):

"Empirical evaluations on ABP and ECG signals from MIMIC-III and VitalDB demonstrate that VABAM consistently outperformed competing approaches in waveform shape factorization and amplitude controllability, achieving higher ISCORE values and smaller discrepancies among the AM, GM, and HM than other models. This advantage arises from conditioning on the cascaded filtering mechanism, which explicitly partitions waveform shape and amplitude into separate generative pathways. This separation allows each component to be modeled independently, yielding clearer shape representations and finer amplitude control. In contrast, the VAE-, diffusion-, and autoregressive-based models in our experimental setting rely on PSD conditioning, which provides only statistical summaries of frequency-domain power and does not encode phase or time-localized shape information, thereby limiting effective shape–amplitude decoupling. Furthermore, the stochastic or autoregressive nature of the diffusion and WaveNet architectures appears to reduce the controllability by attenuating the conditioning effect."

Comment B.13

>> Overall, it seems somewhat odd to use only one final metric within the work instead of building an average per base-metric per model. I.e. the computation of the ISCORE seems to be somewhat arbitrary (which is linked to the previous issue).

- ➔ We thank the reviewer for this important comment. As addressed in ***Comment B.11***, we have removed the previously complex non-linear computation and now adopt the averaging approach as suggested. Moreover, inspired by your feedback, we now report **three complementary averaging schemes**—arithmetic, geometric, and harmonic means—which not only provide a transparent method of integration but also enable examination of the **balance among individual metrics.**
- ➔ This addition proved highly informative. For instance, diffusion-based models demonstrate strong *reconstruction* and *spectral similarity* but perform poorly in *amplitude controllability*. Since the primary goal of this study is not solely high reconstruction fidelity but **effective decoupling** and **amplitude-specific modulation**, such imbalance cannot be considered indicative of strong overall performance. This imbalance can be explicitly captured by examining performance across the three averaging schemes. Accordingly, we have revised the ISCORE formulation to reflect this perspective.

Minor Issues:

Comment B.14

• In the abstract it is unclear whether the proposed method is focused on the generation of synthetic signals based on multiple input entities (e.g. ECG and ABP) or if the model is meant to synthesize from a single input entity that drives the generative process. The confusion is caused by the second sentence where the authors state: "Controlled synthesis of such signals enables …" which reads as the synthesis of ABP and ECG.

- ➔ We appreciate the reviewer's observation regarding the potential ambiguity in the abstract. The phrase *"Controlled synthesis of such signals enables …"* was intended as a general statement highlighting the broader significance of physiological signal synthesis, rather than describing the specific operation of our model. Accordingly, this sentence has been retained to preserve the general context.
- ➔ However, to prevent misunderstanding, we have clarified in the revised abstract that "*We present **VABAM**, a generative framework that* ***operates on a single physiological signal***", explicitly specifying that the proposed framework is designed for single-signal generation rather than multi-entity synthesis (**page 1, line 24**).

Comment B.15

• The normalization process of the metrics (line 165) should be clarified.

- ➔ We appreciate the reviewer's comment. The sentence originally referring to the normalization process (line 165) was included because the metrics were normalized for visualization in the former radar plot. Since **this figure has been removed** in the revised version, the **corresponding sentence has also been deleted** from the manuscript.
- ➔ As an additional clarification, in the current version, all evaluation metrics have been normalized to the range of 0–1 to enable direct comparison across heterogeneous evaluation criteria.

Comment B.16

• Table 1b) shows a set of hyperparameters but only one hyperparameter is displayed (i.e. C=5s instead of C=5s for J=XX and Zeta=YY).

- ➔ We appreciate the reviewer's careful observation. Following the major revision, the former ***Table 1b*** now corresponds to ***Tables D7–D9* in *Appendix D*** (**page 30-32**). We apologize for any confusion caused by the earlier version, which did not include sufficient explanation in the caption.
- ➔ In this study, sensitivity analysis was conducted by fixing one hyperparameter at a time and averaging performance across the remaining parameters. Although a specific configuration such as $C = 5\,\mathrm{s}$ for $J = XX$ and $\zeta = YY$ could be reported, a single result from one setting would not provide reliable insight. To address this limitation, we considered averaging the outcomes of multiple experimental runs under identical configurations; however, this approach was ultimately not pursued because of its high computational cost, spanning two data sources, two signal types, seven benchmark

models, eight VABAM variants, and two ablation versions. Instead, we adopted a computationally efficient strategy designed to **isolate the effect of each parameter while maintaining experimental consistency** across all conditions.

➔ In the revised manuscript, tables have been substantially revised to provide a clearer and more detailed presentation of the hyperparameter sensitivity analysis, including standard deviation values (**page 30-32, Tables D7-D9**). The corresponding table captions have also been clarified as follows:

"Results are presented as mean (s.d.) across independent experiments for each parameter setting."

Comment B.17

• Fig. 3: There are two different ECG and ABP signals shown for 3 different methods. It seems that each of the ECG/ABP signals belongs to either the MIMIC3 or the VitalDB database but this indication is missing.

➔ We thank the reviewer for this helpful observation. The figure previously referred to as *Figure 3* now corresponds to *Figure 7* (**page 12**) in the revised manuscript. In the updated version, the **dataset origins** (*MIMIC-III* and *VitalDB*) **are clearly indicated** as panel headers at the top of the figure, eliminating any ambiguity regarding the source of each ABP and ECG signal.

Summary:

Comment B.18

In summary, while the proposed framework is innovative and technically sophisticated, the evaluation methodology and clarity of presentation require substantial improvement before the claims of superiority over the state of the art can be considered convincing.

➔ We sincerely appreciate the reviewer's thoughtful and constructive feedback. In response, we have **substantially revised** the *Methods*, *Results*, and *Discussion* sections to **enhance methodological transparency, clarify experimental procedures, and provide more comprehensive comparisons**. We have also refined the figures, tables, and captions to improve readability and consistency, ensuring that the revised manuscript presents our contributions in a clearer and more balanced manner.

Reviewer #3 (Remarks to the Author):

I co-reviewed this manuscript with one of the reviewers who provided the listed reports. This is part of the Nature Communications initiative to facilitate training in peer review and to provide appropriate recognition for Early Career Researchers who co-review manuscripts.

- ➔ We sincerely thank **Reviewer 3** for the valuable and insightful comments, which have greatly helped us improve the quality of the manuscript. Before reading the point-by-point responses, we kindly suggest that the reviewer **first** refer to the "**General Response to the Editor and All Reviewers (Summary of Revisions)"** presented on page 1, which provides an overview of the major revisions and will assist in understanding the overall context of our changes.
